# Plant expression of NifD protein variants resistant to mitochondrial degradation

**DOI:** 10.1073/pnas.2002365117

**Published:** 2020-08-31

**Authors:** Robert S. Allen, Christina M. Gregg, Shoko Okada, Amratha Menon, Dawar Hussain, Vanessa Gillespie, Ema Johnston, Rosangela Devilla, Andrew C. Warden, Matthew Taylor, Keren Byrne, Michelle Colgrave, Craig C. Wood

**Affiliations:** ^a^Agriculture and Food, Commonwealth Scientific and Industrial Research Organisation, Acton, Canberra, ACT 2600, Australia;; ^b^Land and Water, Commonwealth Scientific and Industrial Research Organisation, Acton, Canberra, ACT 2600, Australia;; ^c^Agriculture and Food, Commonwealth Scientific and Industrial Research Organisation, Queensland Biosciences Precinct, St. Lucia, Brisbane, QLD 4067, Australia;; ^d^Australian Research Council Centre of Excellence for Innovations in Peptide and Protein Science, Queensland Biosciences Precinct, St. Lucia, QLD 4067, Australia

**Keywords:** nitrogenase, metabolic engineering, synthetic biology, mitochondria, protein engineering

## Abstract

To engineer Mo-dependent nitrogenase function in plants, expression of the structural proteins NifD and NifK will be an absolute requirement. Although mitochondria have been established as a suitable eukaryotic environment for biosynthesis of oxygen-sensitive enzymes such as NifH, expression of NifD in this organelle has proven difficult due to cryptic NifD degradation. Here, we describe a solution to this problem. Using molecular and proteomic methods, we found NifD degradation to be a consequence of mitochondrial endoprotease activity at a specific motif within NifD. Focusing on this functionally sensitive region, we designed NifD variants comprising between one and three amino acid substitutions and distinguished several that were resistant to degradation when expressed in both plant and yeast mitochondria. Nitrogenase activity assays of these resistant variants in *Escherichia coli* identified a subset that retained function, including a single amino acid variant (Y100Q). We found that other naturally occurring NifD proteins containing alternate amino acids at the Y100 position were also less susceptible to degradation. The Y100Q variant also enabled expression of a NifD(Y100Q)–linker–NifK translational polyprotein in plant mitochondria, confirmed by identification of the polyprotein in the soluble fraction of plant extracts. The NifD(Y100Q)–linker–NifK retained function in bacterial nitrogenase assays, demonstrating that this polyprotein permits expression of NifD and NifK in a defined stoichiometry supportive of activity. Our results exemplify how protein design can overcome impediments encountered when expressing synthetic proteins in novel environments. Specifically, these findings outline our progress toward the assembly of the catalytic unit of nitrogenase within mitochondria.

## Significance

Engineering nitrogenase in plants may help alleviate economic and environmental issues due to the use of nitrogen fertilizer. Mitochondria have shown promise in supporting the function of nitrogenase, including electron donation and metallocluster assembly. Despite these successes, formation of the catalytic unit, NifDK, has proven difficult. Here, we find that when relocated to plant mitochondria, NifD is subject to errant peptidase-based cleavage and is insoluble. Guided by NifD sequence variation amongst bacteria and structural modeling, we designed NifD variants that avoided cleavage and retained function in bacterial assays. Fusion of NifK to degradation-resistant NifD also improved solubility, and the polyprotein retained function in bacterial assays. This work advances efforts to produce crops less reliant on nitrogen fertilizer.

Industrial nitrogen fixation is the largest contributor to the world’s population expansion over the past century (1). It is remarkable that this chemical process requiring high temperature (>400 °C) and pressure (150 atm) can be accomplished at room temperature by the bacterial enzyme nitrogenase. Harnessing this enzyme for ammonia production in plants has been a longstanding goal of biotechnology (2). More recently, increasing environmental pollution associated with fertilizer production and usage, as well as food-security concerns, has lent urgency to this aspiration (3).

Nitrogenase is one of the most complex metalloproteins known in nature (4). The mature enzyme contains an FeMo cofactor (FeMoco) at the active site within an α_2_β_2_ heterotetramer comprising NifD and NifK subunits. The Fe protein, also denoted here as NifH, is required for electron transfer to NifDK. Nitrogenase biosynthesis and function also require a range of Nif proteins for assembly of the metal clusters and electron transport to NifH (5). In addition to this biosynthetic complexity, both NifDK and NifH are irreversibly destroyed by oxygen (6).

Recently, several significant advances in engineering nitrogenase componentry in eukaryotes have been made. First, expression and ex vivo function of NifH in yeast mitochondria and plant chloroplasts have validated these organelles as suitable for reconstitution of oxygen-sensitive nitrogenase components (7–9). However, activity of plant chloroplast-expressed NifH is low and requires either substantially reducing ambient oxygen concentrations or harvesting material during the nonphotosynthetic dark period (8). NifB, another oxygen-sensitive enzyme and an essential component of metal-cluster biogenesis, has also been successfully produced in yeast mitochondria and been shown to accumulate NifB-co, a precursor for FeMoco (10, 11). Finally, individual expression of 16 Nif proteins from *Klebsiella oxytoca* in plant mitochondria has demonstrated the feasibility of targeting all of the biosynthetic and structural Nif proteins to this organelle, although mitochondrial processing and expression was suboptimal for certain Nif proteins (12).

Despite advances in producing functional NifH and NifB within mitochondria, expression of NifD remains a problem in both yeast and plants (12, 13). In yeast mitochondria, Burén and colleagues (13) were able to produce NifDK tetramers, but it was observed that the NifD protein was susceptible to degradation, producing a truncated protein of ∼48 kDa. This degradation product also copurified with the NifDK tetramer, possibly accounting for the lack of NifDK function. Our previous work targeting *K. oxytoca* NifD to plant mitochondria has also identified the same degradation phenomenon, with the occurrence of an ∼48-kDa NifD degradation band as a dominant product. In addition to degradation, we were also unable to express NifD in *Nicotiana benthamiana* at a similar abundance as NifK. This imbalance presented a further problem, as these catalytic partners are required to be expressed in a 1:1 stoichiometric ratio for optimal nitrogenase activity. To address this problem, we designed a translational NifD–linker–NifK polyprotein, allowing expression of the polyprotein in a 1:1 stoichiometric ratio. Although function of our polyprotein was untested, similar translational fusion strategies between NifD and NifK have retained a degree of function (14, 15). These encouraging results indicated that translational fusions of Nif proteins can both simplify the genetic componentry and retain nitrogenase function.

Here, we address the issue of NifD degradation within plant mitochondria. Using molecular and proteomic analyses, we discovered that a specific motif within NifD is cleaved by the mitochondrial processing peptidase (MPP) to produce a smaller product. We found that this site of degradation lies within one of the most functionally sensitive regions of the protein. To design variants that were both resistant to degradation and functional, we considered NifD protein diversity, structure, and the substrate specificity of the MPP. Finally, we show that a translational fusion of NifD and NifK that is resistant to degradation enables expression of a soluble polyprotein in mitochondria, which when assayed in *Escherichia coli* retains nitrogenase function.

## Results

### NifD Is Subject to Secondary Cleavage within Plant Mitochondria.

**Fig. 1.**
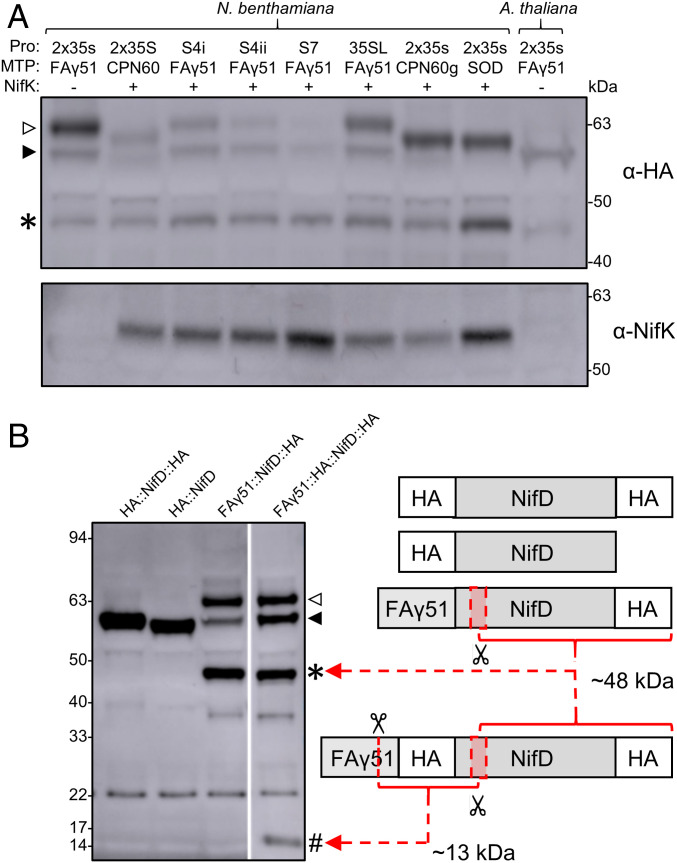
A mitochondrial endoprotease degrades NifD. (*A*) Western blots (α-HA and α-NifK) of protein extracts after introduction of *MTP::NifD::HA* and *MTP::NifK* constructs into *N. benthamiana* and *A. thaliana*. Coinfiltration with MTP:NifK is indicated by + or −. A series of different promoters and MTPs were used to target each construct to mitochondria (*SI Appendix*, Fig. S1 and Tables S1 and S2 and Dataset S1). Open and filled triangles indicate unprocessed and processed forms of FAγ51::NifD::HA, respectively. An asterisk indicates the ∼48-kDa NifD secondary product. (*B*) Western blots (α-HA) of protein extracts for cytosolic (HA:NifD and HA::NifD::HA) or mitochondria (FAγ51::NifD::HA and FAγ51::HA::NifD::HA) targeted NifD constructs. A schematic of the constructs is shown (not to scale). Red dashed arrows indicate C- and N-terminal cleavage products found for FAγ51::HA::NifD::HA. Red shaded boxes represent an approximate area (not to scale) where cleavage was predicted to occur based on the sizes of the degradation products. MTP::NifD::HA products are designated as follows: open triangle, unprocessed; filled triangle, correctly processed; asterisk, secondary C-terminal ∼48-kDa cleavage product; and hash, secondary N-terminal cleavage product at ∼13 kDa. The band at ∼21 kDa is an unspecific background band.

Previous attempts to express NifD in mitochondria of *N. benthamiana* produced an ∼59-kDa NifD protein of low abundance and a distinct lower-molecular-mass product of ∼48 kDa (12). This ∼48-kDa product could be undesirable for several reasons, including interference with formation of the NifDK catalytic unit. We first wanted to test whether differing mitochondrial targeting peptides (MTPs) or constitutive promoter combinations may influence the accumulation of this secondary product. For this purpose, we reconfigured our cloning protocols to the GoldenGate system to allow easier interchangeability of these components (16, 17) (*SI Appendix*, Fig. S1 and Tables S1 and S2 and Dataset S1). We then coinfiltrated *N. benthamiana* leaves with various *MTP::NifD::HA* constructs together with *MTP::NifK*. Western blot analysis of protein extracts from the infiltrated leaf cells revealed that all constructs produced some correctly processed NifD, although for most constructs, we observed another upper band that corresponded to an unprocessed form of NifD that was not imported into the mitochondria or processed by the MPP. Most importantly, however, the ∼48-kDa product previously observed was still present for all NifD-expressing constructs, regardless of promoter or MTP (Fig. 1*A*). The accumulation of this ∼48-kDa degradation product was also independent of whether *MTP::NifD* was infiltrated as a single gene vector, coinfiltrated with *MTP::NifK* (as independent vectors), or infiltrated together with *MTP::NifK* on a multigene vector (*SI Appendix*, Fig. S2). The degradation product was also observed in leaves in stably transformed *Arabidopsis thaliana* (Fig. 1*A*).

To determine the origin of the ∼48-kDa product, we considered two possibilities, downstream translation or protein degradation. The *nifD* gene contains several downstream *ATG* codons which could lead to an ∼48-kDa translation product. Therefore, the second to fifth *ATG* codons downstream of the *NifD* start codon were replaced with alternate codons, and this protein was expressed as an MTP fusion construct in *N*. *benthamiana* (pRA30; *SI Appendix*, Fig. S1 and Table S1). However, the ∼48-kDa product was still present in *N. benthamiana* protein (*SI Appendix*, Fig. S3), refuting the hypothesis that it arose from downstream *ATG* translation. Therefore, we concluded that the ∼48-kDa product was degraded NifD protein.

We assessed whether NifD degradation was a consequence of targeting the protein to the mitochondria. To test this, we made two cytosolic NifD constructs, HA::NifD and HA::NifD::HA, with the latter allowing the detection of any potential C-terminal degradation products (Fig. 1*B*). We found that *N. benthamiana* protein extracts from both constructs produced a discrete band of the size expected for the full-length NifD protein (Fig. 1*B*). Importantly, there was no ∼48-kDa C-terminal degradation product observed for HA::NifD::HA, in contrast to the mitochondria-targeted NifD, MTP::NifD::HA. Additionally, there was no N-terminal degradation product observed for HA::NifD or HA::NifD::HA. Together, these results demonstrated that the degradation of MTP::NifD was a consequence of mitochondrial targeting.

### Cleavage of NifD within Plant Mitochondria Occurs via Site-Specific Endoprotease Activity.

We next determined whether NifD degradation was a consequence of exoprotease or endoprotease activity. Given that we could detect an NifD C-terminal HA-tagged degradation product, detection of a corresponding N-terminal degradation product could confirm endoprotease activity. Accordingly, a construct was introduced into *N. benthamiana*, where an HA tag was also included directly after the MTP, *MTP::HA::NifD::HA* (Fig. 1*B*). Thus, endo-specific cleavage could be expected to produce two HA-tagged products—the longer, ∼48-kDa C-terminal product seen previously in MTP::NifD::HA extracts and a shorter, ∼13-kDa N-terminal product. Western blot analysis of protein extracts revealed a specific shorter, ∼13-kDa N-terminal product and the longer, ∼48-kDa C-terminal product (Fig. 1*B*). This result demonstrated that the cleavage of MTP::NifD was site-specific, and not a result of exoprotease degradation from the N terminus. We therefore describe this ∼48-kDa product as a “secondary cleavage product,” arising from endopeptidase activity at a region downstream of the MTP processing site.

### The Secondary Cleavage Region within NifD Is Characteristic of an MPP Processing Site.

**Fig. 2.**
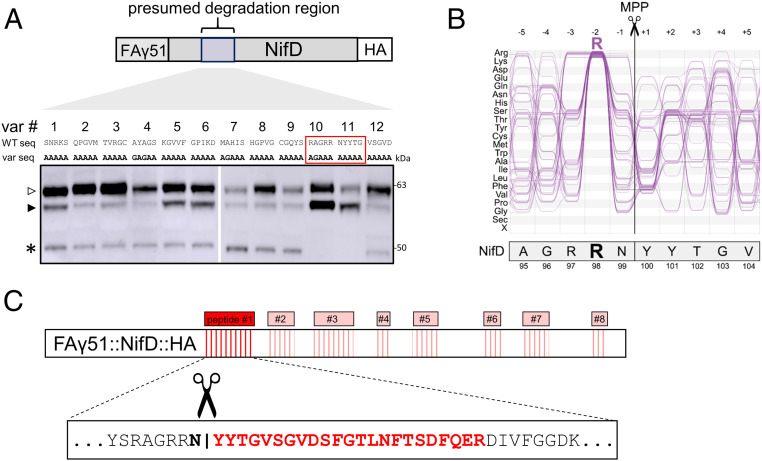
Discovery of the NifD degradation region reveals a characteristic MPP site. (*A*) Western blots (α-HA) of protein extracts after introduction of *MTP::NifD::HA* genetic constructs coinfiltrated with MTP::NifK into *N. benthamiana* leaf cells. Constructs were based on FAγ51::NifD::HA (wild-type NifD), except wild-type amino acids were substituted in five amino acid alanine/glycine blocks (variants [var] 1 to 12). The initial region for broad coverage with alanine/glycine scanning is shown schematically at the top (not to scale), with the wild-type NifD sequence, and underneath the corresponding five amino-acid changes for each individual construct. MTP::NifD::HA bands are designated by open and filled triangles for unprocessed and correctly processed forms, respectively; the asterisk indicates the secondary cleavage product. (*B*) Comparison of amino acids 95 to 104 of NifD with a typical −2R MPP cleavage motif. The motif was visualized by using selected sequences from Δ*icp55* and wild-type *A. thaliana*, previously identified by Carrie et al. (27) and Huang et al. (26). For a complete set of mitochondrial cleavage sites, refer to *SI Appendix*, Fig. S4. Numbers at the top refer to the amino-acid position relative to the cleavage site. The sequences shown here contain a conserved Arg residue in position −2, characteristic of a −2R MPP cleavage site. NifD also contains an Arg in position −2 relative to the experimentally determined cleavage site. (*C*) A schematic of the MS identification of peptides from the degradation product. Eight peptides (red boxes; not to scale) were identified. The most N-terminal peptide is shown in detail in red, identifying the precise cleavage site shown by scissors.

The results of the experiments described above indicated that secondary cleavage of MTP::NifD was a consequence of mitochondrial targeting and occurred at a specific location within the NifD sequence. As we could not precisely distinguish this location based on the sizes of the degradation products, we first designed a series of MTP::NifD variants, each with a block of five consecutive amino-acid substitutions (alanine/glycine scanning) within the approximate region (amino acids 49 to 108) of secondary cleavage within NifD (Fig. 2*A*). We wanted to test if any of these variants (NifD variants 1 to 12) would disrupt secondary cleavage.

Of the 12 variants tested, protein extracts for 10 still produced the ∼48-kDa secondary cleavage product (Fig. 2*A*). However, variants 10 and 11 were conspicuous in showing no ∼48-kDa cleavage product and a higher abundance of the correctly processed protein at ∼56 kDa. Based on the substitutions in NifD variants 10 and 11, we concluded that a specific region of the NifD protein within the amino acid sequence RAGRRNYYTG was required for the secondary cleavage of NifD in mitochondria. Using the MitoFates prediction program (18), we found that NifD contained a predicted MPP cleavage site in this region, after the asparagine residue, RAGRRN↓YYTG. This motif has similarities to the typical “-2R” motif found in ∼55% of mitochondrial targeted proteins (Fig. 2*B* and *SI Appendix*, Fig. S4 *A*–*D*). Therefore, this secondary cleavage site within NifD appeared similar to an MPP cleavage motif.

### Isolation of the NifD Degradation Product Identifies the Specific Site of Endoprotease Cleavage.

To determine the N-terminal sequence of the degradation product, the ∼48-kDa fragment was gel-excised and subjected to proteomic analysis (*SI Appendix*). In total, eight specific NifD peptides were identified downstream of the region identified from the alanine/glycine scanning approach described above (Fig. 2*C*). No peptide was found for the complete tryptic peptide RNYYTGVSGVDSFGTLNFTSDFQER; however, the expected mass of the shorter peptide YYTGVSGVDSFGTLNFTSDFQER was positively identified. Therefore, this shorter peptide must have arisen from cleavage of the NifD sequence within the RRNYY sequence—between the asparagine (N) and tyrosine (Y) residues, followed by the tryptic digestion in the analysis. This analysis showed that the actual size of the degradation product is 44.5 kDa, and this degradation band runs slightly slower at ∼48 kDa on the sodium dodecyl sulfate (SDS)/polyacrylamide gel. The identification of the cleavage site by this proteomic analysis concurred with the alanine/glycine scanning approach described above and precisely matched the in silico prediction as an MPP cleavage site.

### Design and Functional Validation of NifD Variants Resistant to Mitochondrial Degradation.

Although we could abolish secondary cleavage of NifD via five amino-acid modifications at the site of MPP activity, it was likely that such changes would disrupt function. We aimed to introduce discrete changes within NifD to potentially reduce or eliminate cleavage while maintaining function. Our design was guided by structural modeling and examining the sequence diversity of other naturally occurring NifD sequences at positions corresponding to RAGRRNYYTG of *K. oxytoca*. Our focus was on replacing amino acids in position −3 to +3 relative to the cleavage site (RRN↓YYT). We modeled modifications that would potentially disrupt cleavage while minimizing introduction of adverse interactions, such as steric clashes, or disruptions to existing hydrogen bonds or electrostatic interactions.

**Fig. 3.**
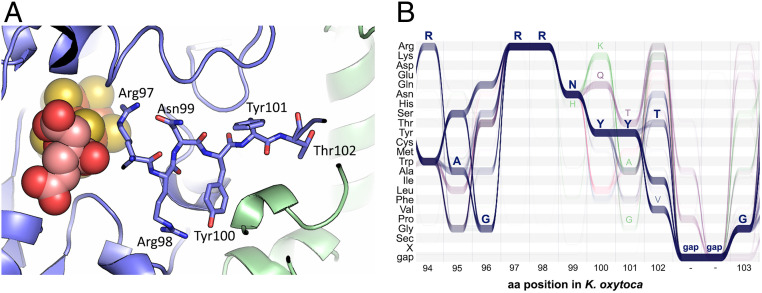
(*A*) Location of the proposed secondary cleavage site RRN↓YYT shown in the crystal structure of the MoFe protein from *K. pneumoniae* (Protein Data Bank ID code1QGU). NifD is shown in blue, and NifK is shown in green. FeMoco is shown as spheres. The residues of the proposed cleavage site are represented by sticks. The cleavage site is in proximity to FeMoco and close to the interface of NifD and NifK. The numbering of NifD amino acids within the crystal structure was adjusted to correspond to the numbering of the full-length sequence, which contains two methionine residues at the start of the sequence. (*B*) ALVIS analysis of the amino acid (aa) distribution based on 1,476 putative NifD proteins around the proposed secondary cleavage site. The *y* axis shows the amino acid residue, and the *x* axis shows the position of the corresponding residues in *K. oxytoca*. The residues of the *K. oxytoca* NifD sequence “RAGRRNYYTG” are shown above in blue. Sequences that contain a Gln and Lys in position 100 are shown in purple and green, respectively. Amino acid residues that were present in variants are shown above (H99, K100, Q100, T101, A101, G101, and V102). *SI Appendix*, Table S3 lists the frequency distribution of the equivalent residues of *K. oxytoca* RRNYYT in detail.

The crystal structure of the MoFe protein from *Klebsiella pneumonia* revealed that the MPP secondary cleavage site within NifD is in close proximity to FeMoco and NifK (Fig. 3*A*). Specifically, Arg97 interacts with the bridging S5 sulfido ligand of FeMoco and is thought to stabilize the more reduced edge of the cluster (19). Arg98 is involved in a network of hydrogen-bonding and electrostatic interactions within NifD and also interacts with Asp516 of the NifK protein. Asn99 accepts a hydrogen bond from Arg97, and Tyr100 interacts with both Arg98 of NifD and Asp516 of NifK, thus contributing to the thermostability of NifDK. We therefore created variants which contained substitutions in the less functionally sensitive residues Tyr101 and Thr102 (i.e., variants Y101F, T102A, and Y101F/T102A). As we were not confident that these variants described would prevent cleavage by MPP, we also included variants that substituted amino acids Arg98 and other sites (R98K, R98K/Y101F, R98K/T102A, R98K/Y101F/T102A, and R98K/Y101A/T102A), recognizing that these may not be functional.

A second approach involved the analysis of the sequence diversity among naturally occurring NifD sequences. For this analysis, a set of 1,476 naturally occurring putative NifD sequences from a range of bacterial and archaeal sources was extracted from the Interpro database (20) (*SI Appendix*, Table S3). The sequences were aligned and visualized by using ALVIS (21) (Fig. 3*B*). The alignment revealed that both Arg97 and Arg98 were completely conserved, which is not surprising, given their proximity to FeMoco. Overall, the alignment also showed that sequence conservation decreased from residue Asn99 to Thr102 (*SI Appendix*, Table S3). Accordingly, we also designed variants where these residues were replaced with the second-most-common residues from the set of NifD sequences, i.e., N99H, Y100Q, Y101A, and T102V, respectively. Additionally, amino acids 100 and 101 were replaced with the third- and fourth-most-common residues, i.e., Y100K and Y101T, respectively. Furthermore, we noticed some amino acid dependencies and created variants in which two or three amino acids were replaced, i.e., Y100Q/Y101T, N99H/Y100K/Y101G, and Y100K/Y101A.

**Fig. 4.**
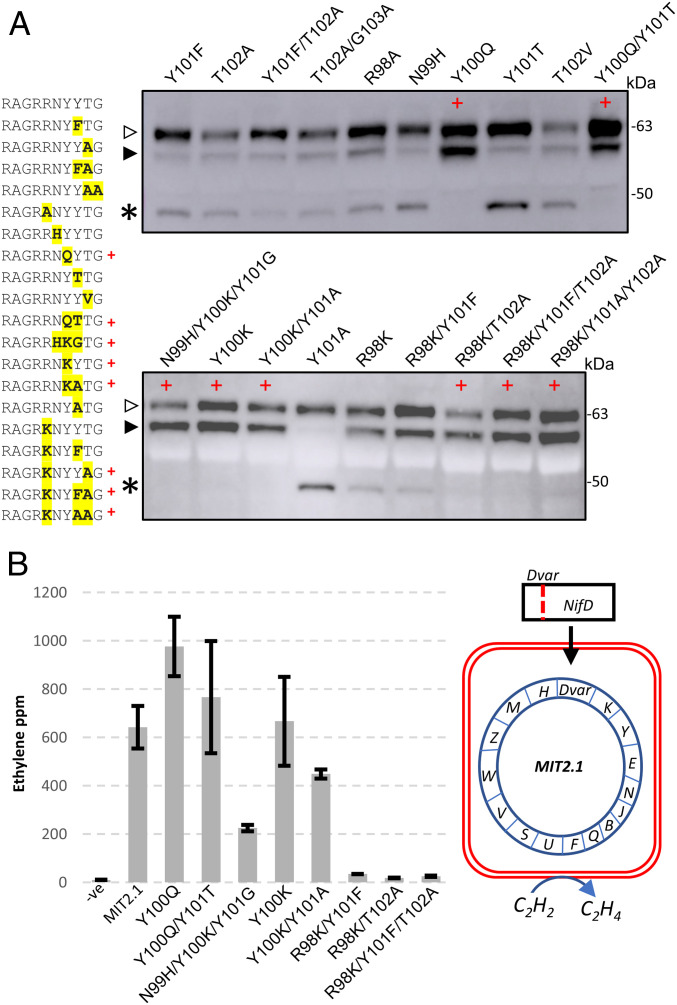
Identification and functional analysis of cleavage-resistant NifD variants. (*A*) Western blot (α-HA) of protein extracts after introduction of *MTP::NifD::HA* variant constructs into *N. benthamiana* leaf cells coinfiltrated with *MTP::NifK*. Discrete amino acid changes compared to wild type are shown in bold/yellow, and variants found to be degradation resistant are identified by a red +. Open and filled triangles designate unprocessed and correctly processed forms of MTP::NifD, respectively; the asterisk indicates the secondary cleavage product. (*B*) ARAs of nitrogenase activity with variant NifD proteins in *E. coli*. A subset of the same modifications tested above were individually introduced into MIT v2.1, and ethylene was measured. Error bars represent the SEM from at least two biological replicates. A depiction of the MIT v2.1 assay is shown, where substitutions to NifD are incorporated into the nitrogenase cluster (for further details, see *SI Appendix*, Fig. S7). Negative controls contained no MIT v2.1 cluster, and positive controls included the cluster with the wild-type NifD sequence. Ppm, parts per million.

In total, 19 substitution variants were designed (Fig. 4), and these were coinfiltrated as *MTP::NifD::HA* variant constructs into *N. benthamiana* with *MTP:NifK*, and leaf protein was analyzed by Western blotting. We identified several variants (Fig. 4*A*) that did not produce the secondary cleavage product (Y100Q, Y100Q/Y101T, N99H/Y100K/Y101G, Y100K, Y100K/Y101A, R98K/T102A, R98K/Y101F/T102A, and R98K/Y101A/T102A). Most remarkably, two variants of this degradation-resistant set had single-amino-acid substitutions at the Y100 residue, Y100Q and Y100K. We also assessed NifD–Y100Q in the absence of NifK and found that resistance to degradation was unaffected (*SI Appendix*, Fig. S5). Interestingly, some variants showed degradation, despite prediction software indicating otherwise (e.g., R98A) (18).

To confirm that the proteins were indeed located in mitochondria of *N. benthamiana*, we performed a mitochondrial purification using an affinity-tag-based technique similar to those reported (22, 23). We designed a fusion protein with a Twin-streptag consisting of mTurquoise fluorescent protein and metaxin, a mitochondrial outer membrane protein (Twin-strep::mTurquoise::metaxin). The expression of this construct allowed the rapid purification of mitochondria by using streptavidin magnetic beads (*SI Appendix*, Fig. S6 *A* and *B*). When we coexpressed *MTP::NifD* or *MTP::NifD-Y100Q* with NifK and purified plant mitochondria using the metaxin fusion protein, Western blot analysis showed that for MTP::NifD, the degradation product was accumulating in mitochondria, while for MTP::NifD-Y100Q, only correctly processed NifD was detected (*SI Appendix*, Fig. S6*C*). These results confirm that MTP::NifD and the degradation-resistant variant MTP::NifD(Y100Q) are imported into the plant mitochondria.

We next wanted to assess the functional consequences of these mutations that provided resistance to NifD degradation. For this purpose, we utilized a nitrogen-fixing *E. coli* strain harboring a modified *K. oxytoca* nitrogenase gene cluster (MIT v2.1) (24) (Fig. 4*B* and *SI Appendix*, Fig. S7). Within MIT v2.1, we introduced mutations in NifD corresponding to a selection of the plant-expression variants tested above. We then measured nitrogenase activity of these variants via an acetylene reduction assay (ARA). From our analysis, we identified three variants that retained function similar to wild-type levels (Y100Q, Y100Q/Y101T, and Y100K) and others that either had diminished or negligible activity levels (Fig. 4*B*).

### The Y100Q Substitution Protects a NifD–Linker–NifK Polyprotein from Mitochondrial Degradation at the RRNYYT Motif.

**Fig. 5.**
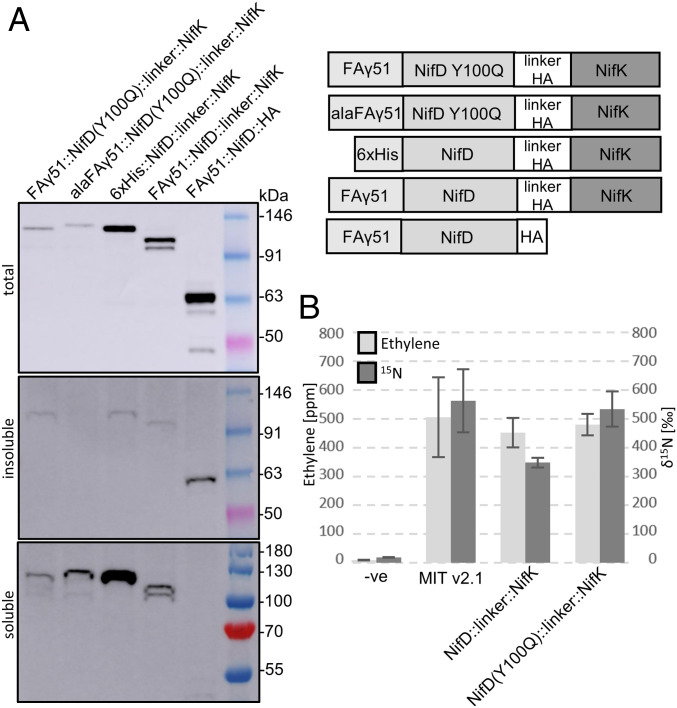
MTP::NifD(Y100Q)–linker–NifK polyprotein is degradation-resistant, soluble, correctly processed in plant mitochondria, and functional when expressed in *E. coli*. (*A*) Western blots (α-HA) of protein extracts after introduction of NifD genetic constructs into *N. benthamiana* leaf cells. The alaFAγ5::MTP is a modified version of FAγ51 that has been alanine-scanned to disrupt mitochondrial processing, thus producing a larger unprocessed protein. A schematic of each construct is described. Proteins were separated into soluble, insoluble, and total fractions. Insoluble and soluble fractions run slightly faster than total fractions due to different buffer conditions. (*B*) ARAs and ^15^N incorporation assays compare NifD–linker–NifK function to wild type in *E. coli*; also see *SI Appendix*, Fig. S7. For each modification, three biological replicates were measured in duplicate; error bars represent SEM.

Our observations that MTP::NifD is subject to secondary cleavage led us to evaluate if degradation is also occurring in a MTP::NifD–linker–K polyprotein (12). For this approach, we designed an NifD–linker–NifK GoldenGate-based construct by placing the HA epitope within the linker peptide, avoiding any extension to the C-terminal of NifK. We then introduced the Y100Q variation into MTP::NifD–linker–NifK. To distinguish processing at the canonical MTP cleavage site, a third construct was made that contained a modified MTP with alanine substitutions at the canonical cleavage site to avoid cleavage by the MPP (*alaMTP::NifD*–*linker*–*K*). Finally, a cytosolic control construct was designed that contained a His-tag instead of the MTP (*6*×*His::NifD*–*linker*–*K*) (Fig. 5*B* and *SI Appendix*, Tables S1 and S2). Protein produced from this construct approximated the size of correctly processed MTP::NifD–linker–K protein.

These constructs were infiltrated separately into *N. benthamiana* leaves, and protein fractions were analyzed by Western blot. Total protein extracts of MTP::NifD(Y100Q)–linker–K produced a single band that aligned with the cytosolic NifD–linker–K and migrated faster than the control for the unprocessed protein (Fig. 5*A*). This migration speed corresponded to correctly processed and undegraded MTP::NifD(Y100Q)–linker–K polyprotein. By contrast, the *MTP::NifD*–*linker*–*K* construct produced two lower bands indicative of degradation. Interestingly, while MTP::NifD::HA was incompletely processed, the MTP::NifD(Y100Q)–linker–K polyprotein was completely processed at the canonical MPP processing site within the MTP (Fig. 5*A*).

### The NifD–Linker–NifK Polyprotein Is Soluble in Plant Mitochondria and Functional in *E. coli*.

Solubility of nitrogenase proteins within mitochondria is considered a prerequisite for function. However, a recent report has indicated that targeting *Azotobacter vinelandii* NifB to the mitochondria produced an insoluble protein (11). Therefore, we assessed the solubility of our mitochondria-targeted NifD proteins. After preparing soluble and insoluble NifD protein fractions from the same leaf spots used for total protein (*Materials and Methods*), we visualized the proportions of proteins in these fractions by Western blot. Whereas cytosolic NifD (HA::NifD) was mostly soluble and mitochondrial MTP::NifK coexpressed with MTP::NifD accumulated partially in the soluble fraction (*SI Appendix*, Fig. S8 *A* and *B*), we were unable to detect MTP::NifD in the soluble fraction (Fig. 5*A*). In contrast, we were surprised to discover that more of the NifD(Y100Q)–linker–NifK polyprotein was observed in the soluble fraction than in the insoluble fraction. Therefore, fortuitously, the NifD(Y100Q)–linker–NifK polyprotein was both resistant to the secondary cleavage and predominantly soluble in plant mitochondria.

We assessed the function of the NifD–linker–NifK polyprotein by introducing the same linker as used for plant expression between the native NifD and NifK proteins from MIT v2.1 and tested for nitrogenase function via ARA. We also introduced the Y100Q variation into this NifD–linker–NifK polyprotein in MIT v2.1. We found that both the NifD–linker–NifK and degradation-resistant NifD(Y100Q)–linker–NifK proteins retained wild-type levels of activity (Fig. 5*C*). Finally, although the ARA serves as a proxy for nitrogenase activity, we wanted to test directly for nitrogen-fixing ability by use of a ^15^N_2_ assay. For both NifD–linker–NifK and NifD(Y100Q)–linker–NifK, ^15^N_2_ reduction was lower, but not significantly (*P* = 0.1238 and *P* = 0.8304, respectively). These results demonstrate that this mutation allows N_2_ fixation for the NifDK polyprotein.

### The Y100Q Variation within NifD Prevents Secondary Cleavage in Yeast Mitochondria.

**Fig. 6.**
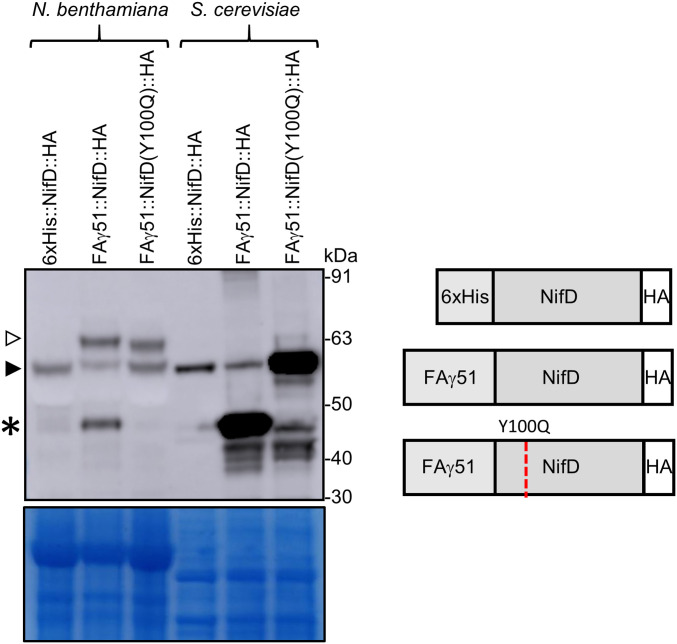
The Y100Q mutation in NifD prevents secondary cleavage in yeast mitochondria. Western blot (α-HA) of protein extracts after introduction of *NifD::HA* genetic constructs into *N. benthamiana* leaf cells coinfiltrated with *MTP::NifK* or *S. cerevisiae* is shown. A schematic of the constructs is shown. The *6*×*His::NifD::HA* construct produces a cytosolic targeted protein of a similar size to correctly processed MTP::NifD::HA. Open and filled triangles indicate unprocessed and processed forms of NifD, respectively. The asterisk indicates the secondary cleavage product. Coomassie-stained gel post transfer is shown underneath.

Yeast have proven a useful model for testing expression and assembly of nitrogenase components in mitochondria (7, 13, 25); however, the problem of NifD degradation has also been reported when expressing *A. vinelandii* NifD in this organelle. Notably, *A. vinelandii* NifD contains the same RRNYYT motif that enables MPP cleavage of *K. oxytoca* NifD in plant mitochondria. We wanted to determine whether *K. oxytoca* NifD also was similarly degraded when expressed in yeast mitochondria and, secondly, whether the Y100Q variation could protect NifD from any possible degradation. For this, we made yeast-expression vectors targeting *K. oxytoca* NifD to either the cytosol, 6×His::NifD:HA, or the mitochondrial matrix, *MTP::NifD::HA*. We also made a yeast-expressed version of the NifD Y100Q variation, *MTP::NifD(Y100Q)::HA* (Fig. 6 and *SI Appendix*, Fig. S9). Besides being expressed via the *GAL4* promoter/terminator, these NifD proteins were otherwise identical to their plant equivalents.

The yeast constructs were expressed in *Saccharomyces cerevisiae*, and total protein was analyzed via Western blot. We found that both plant- and yeast-expressing *K. oxytoca* NifD cytosolic constructs produced a band of the size expected for cytosolic expression of full-length NifD, and no C-terminal degradation products were observed (Fig. 6). However, MTP::NifD produced an intense, smaller band of the same size as the plant-degradation product, whereas much less full-length NifD was produced by this construct. This result indicated that *K. oxytoca* NifD degradation was occurring in yeast mitochondria, possibly at the same RRNYYT motif as occurs in plant mitochondria. Notably, the NifD Y100Q mutation within NifD targeted to yeast mitochondria enabled a much higher level of correctly processed NifD to accumulate (Fig. 6), although smaller products were also observed for all proteins targeted to yeast mitochondria. Together, these results indicated that the wild-type NifD protein from *K. oxytoca* is degraded in the same region when targeted to yeast or plant mitochondria, and secondary cleavage is greatly reduced by the Y100Q variation, resulting in a higher abundance of the full-length NifD protein.

### Natural Variants of the RRNYY Motif Are Less Susceptible to MPP Cleavage in Plant Mitochondria.

Since the *K. oxytoca* NifD variants Y100Q and Y100K were resistant to mitochondrial cleavage in plant mitochondria, we investigated whether NifD proteins from other diazotrophs containing a glutamine or lysine residue in this position were also resistant. To choose representative sequences, we created a sequence-similarity network that grouped similar NifD sequences into different subgroups and analyzed which amino acid was present in position 100, where position 100 corresponds to the numbering in *K. oxytoca* NifD (*SI Appendix*, Fig. S10 *A* and *B*). We then chose three representative sequences from subgroups that contained a glutamine or lysine residue in position 100—i.e., NifD from *Chlorobium tepidum*, *Desulfotomaculum ferrireducens*, and *Desulfovibrio vulgaris*—and assessed their susceptibility to mitochondrial degradation. We also chose three NifD proteins that contained tyrosine in position 100—i.e., NifD from *A. vinelandii*, *Azospirillum brasilense*, and *Sinorhizobium fredii*. The latter three proteins were expected to be cleaved by the MPP, as they contain the same RRNYY motif as NifD from *K. oxytoca*. For each NifD sequence, a construct was prepared that contained MTP-FAγ51 at the N terminus and an HA tag at the C terminus (*MTP::NifD::HA*). These constructs were coexpressed in *N. benthamiana* leaves with their corresponding NifK protein, which contained an HA tag at its N terminus (*MTP::HA::NifK*).

**Fig. 7.**
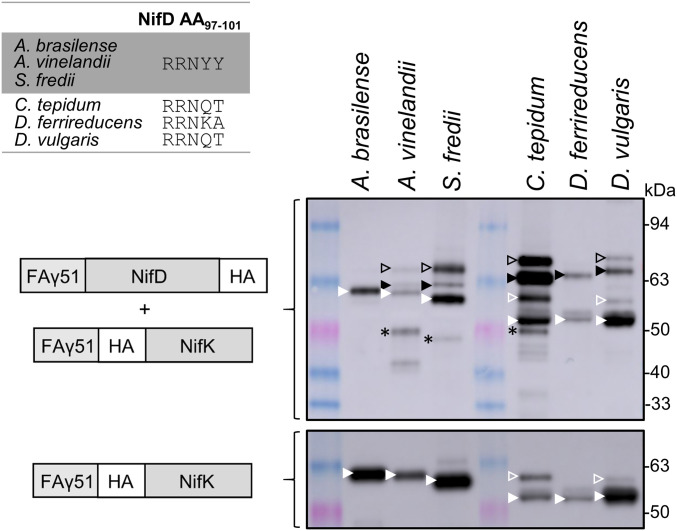
Comparison of the cleavage susceptibility of NifD proteins from other diazotrophs in plant mitochondria. Three NifD proteins tested contained the same RRNYY motif as *K. oxytoca* NifD, while the other three contained either glutamine or lysine in position 100. The upper Western blot (α-HA) shows the coexpression of NifD with each corresponding NifK protein. For comparison, the lower blot (α-HA) shows the expression of NifK only. The NifD and NifK proteins were targeted to mitochondria by using FAγ51. NifD proteins had a C-terminal HA tag, FAγ51::NifD::HA, and NifK proteins had an N-terminal HA tag, FAγ51::HA::NifK. The HA tag was added to NifK, as we were not able to use the NifK antibody for the detection of certain NifK proteins due to a reduced affinity. The bands for unprocessed and processed proteins are marked by an open and filled triangle, respectively. Triangles are black for NifD and white for NifK. The NifD degradation product is marked by an asterisk.

Western blot analysis showed that the ratio of correctly processed NifD and the C-terminal degradation product varied for all six NifD proteins (Fig. 7). The expression level of *A. brasilense* NifD was extremely low, and bands corresponding to *A. brasilense* NifD were hardly detectable. However, further blots revealed that *A. brasilense* NifD was degraded as expected (Fig. 7 and *SI Appendix*, Fig. S11). For both *A. vinelandii* NifD and *S. fredii* NifD, a degradation product of the expected size was observed, indicating MPP cleavage at the proposed site. Interestingly, our analysis in plant mitochondria found a second C-terminal product for *A. vinelandii* NifD, which was not observed in yeast mitochondria (13). No ∼48-kDa degradation product was detected for *D. ferrireducens* NifD or *D. vulgaris* NifD. For *C. tepidum* NifD, another product was detected; however, a product of the same size was also found for cytosolic *C. tepidum* NifD (*SI Appendix*, Fig. S11). This indicates that this product does not originate from cleavage by the MPP. Overall, NifD sequences that contained a glutamate or lysine residue in position 100 were less susceptible to degradation by the MPP.

## Discussion

Mitochondria have potential as an organelle capable of supporting nitrogenase function (7); however, both NifD degradation and insolubility within plant mitochondria present bottlenecks. Our modifications to NifDK have produced a soluble protein that is resistant to secondary cleavage within plant mitochondria and retains nitrogenase function. This study demonstrates that impediments to the transfer of bacterial proteins to novel environments can be overcome by protein design guided by natural variation.

Several strands of evidence imply that the secondary cleavage of NifD is due to the activity of the MPP. First, the identified secondary cleavage site (RRNYY) contains the hallmark of a canonical MPP processing site, namely, the presence of an arginine at position −2 in relation to the scissile bond, characteristic of cleavage motifs found in the majority of matrix-targeted proteins (26). Second, disruption of this site by alanine/glycine scanning, such that the region no longer resembles a canonical MPP cleavage site, prevents the secondary cleavage of NifD. Finally, targeting of NifD to the cytosol, which does not contain an MPP, either in yeast or plants, prevents secondary cleavage.

The fact that the cryptic MPP site resides in one of the most functionally sensitive regions within NifD presented a challenge to engineer or find natural variants that simultaneously avoided cleavage, yet retained function. Guided by structural analysis and natural variation between NifD sequences, we designed several variants of *K. oxytoca* NifD at the secondary cleavage site that prevented NifD degradation in plant mitochondria and retained function [e.g., NifD(Y100Q)]. Interestingly, some of these NifD variants were predicted to be cleaved by the MPP based on in silico modeling (18). Conversely, other variants that were predicted to be resistant to MPP cleavage were degraded. Our results show that, although in silico predictions are a useful guide, these are ultimately constrained by the fact there is no absolute consensus for an MPP cleavage site (27).

We have also identified several naturally occurring NifD proteins that were much less susceptible to degradation than the NifD proteins from the classic diazotrophic model organisms *K. oxytoca* and *A. vinelandii*. This result accords with our findings using synthetic *K. oxytoca* variants, where replacement of the tyrosine at position 100 provided resistance to MPP activity. More broadly, this result demonstrates that it is also possible to exploit the natural diversity of NifD proteins to reduce degradation within plant mitochondria. This is conceptually similar to approaches taken by Burén et al. (10, 11), where the natural diversity of NifB sequences was explored to identify soluble versions for expression within mitochondria.

We identified that degradation of *K. oxytoca* NifD also occurs in yeast mitochondria and that MPP cleavage was reduced by incorporating the Y100Q variation. As *A. vinelandii* NifD also contains an identical internal RRNYY site, it is likely that the degradation seen for *A. vinelandii* NifD, when expressed in yeast observed previously (13), is a consequence of secondary cleavage at this site. Therefore, we expect that an *A. vinelandii* NifD containing the Y100Q mutation would improve the abundance of full-length NifD. As the degradation of *A. vinelandii* NifD has been suggested to present a possible bottleneck to the formation of the correct NifDK heterotetramer, it will be of interest to see if a degradation-resistant variant of *A. vinelandii* NifD may help overcome this problem.

It has been suggested that the degradation product of NifD, when targeted to yeast mitochondria, could arise from the incorrect maturation of the NifDK tetramer (13). For both plant and yeast mitochondria-targeted NifD, we can now discount this possibility, as, even in the absence of NifK, the NifD(Y100Q) variant was not degraded. This implies that, although an NifD variant may be misfolded, it is not necessarily cleaved by the MPP. Since the RRNYY site is buried within NifD in the correctly assembled NifDK tetramer, NifD would likely need to be unfolded or misfolded for cleavage to occur. While the cleavage site could be exposed to the MPP in a misfolded protein that has accumulated in mitochondria, another possibility seems more likely: During mitochondrial import, proteins unfold to be transported through the outer- and inner-membrane transport complexes (28). Therefore, we favor a model where the MPP cleaves the RRNYY motif within the NifD protein when it enters the mitochondrial matrix in an unfolded state.

We have previously observed that, despite use of the same MTP, certain MTP::Nif proteins are processed less efficiently than others (12), and NifD clearly falls into this category. *K. oxytoca* NifD and NifD(Y100Q) were only partially processed at the canonical MPP cleavage site within the MTP. Here, we consider two possible explanations for this phenomenon. Either MTP::NifD is imported into the mitochondrial matrix, but is not well recognized by the MPP; or a proportion of MTP::NifD is not imported and, thus, not accessible to the MPP. Our results from the mitochondrial purification indicate that the latter case is more likely, as we did not observe a band for unprocessed NifD in purified mitochondria. However, the translational fusion of NifD to NifK enabled complete processing of the polyprotein, for reasons that remain unclear. That processing efficiency was improved by fusing the NifK protein to NifD is remarkable, particularly as this synthetic polyprotein (120 kDa) is at the upper size limit of endogenous proteins transported to plant mitochondria (26). Even larger Nif polyproteins have been shown to function in *E. coli* and may provide a means to simplify expression and provide correct stoichiometry (15). It is, therefore, encouraging that mitochondria appear capable of importing and processing larger synthetic proteins, and it will be interesting to test the upper size limits for other nitrogenase polyproteins.

We found that *K. oxytoca* NifD is insoluble when targeted to plant mitochondria, both in the presence and absence of NifK. However, NifD was soluble in the cytosol, even in the absence of NifK. These observations suggest that the mitochondrial environment itself or the mitochondrial targeting of NifD leads to misfolding of the protein. Several other Nif proteins have been shown to be insoluble in yeast or plant mitochondria (11, 29). Remarkably, the translational fusion of NifD to NifK led to a soluble polyprotein within the mitochondria. It is possible that linking these proteins has allowed rapid association of the NifD and NifK components into their native heterotetrametric structure within the mitochondrial matrix. Given that the function of our *K. oxytoca* NifDK polyprotein was not significantly affected in *E. coli*, this further suggests that our rationally designed linker allows for correct folding and assembly of the structure required for function.

Although significant progress has been made toward reconstructing nitrogenase components within mitochondria, many challenges lie ahead, such as the assembly of the nitrogenase metalloclusters, the modulation of *nif* gene-expression levels, and the availability of molybdenum (30). Furthermore, advances in engineering nitrogenase in yeast mitochondria, such as the formation of active NifH and NifB (7, 10), are yet to be realized in plants. In this study, we identified the reason for NifD degradation in mitochondria and have presented solutions using either synthetic or natural variants of NifD. We have also resolved both inefficient mitochondrial processing and solubility of NifD by fusing it to NifK using a rationally designed linker. This is a major advance in the field of nitrogenase engineering, as it allows the stable expression of the key structural protein within plant mitochondria. Further work can now focus on the assembly of the P cluster and FeMoco required for nitrogenase function. To our knowledge, the MPP-dependent protein instability described here has not been previously observed in plant metabolic engineering. For future organelle engineering, and especially when engineering prokaryotic proteins into eukaryotic hosts, it will be important to investigate whether these proteins contain motifs that can interfere with successful targeting and protein stability in the organelle of interest. Special attention should be given to potential cleavage sites that could lead to protease-mediated degradation.

## Materials and Methods

### Construction of Vectors.

Vectors for *N. benthamiana* and *A. thaliana* expression were based on the GoldenGate parts and tool kit (17) and assembled by using the GoldenGate assembly protocol (16). Nonstandard components and NifD variant sequences for plant expression vectors were generated by DNA synthesis. Full parts descriptions are described in Dataset S1. For yeast-expression constructs, NifD flanking sequences were amplified from SN10, SN114, and SN196 by using primers containing KpnI (forward) and SacI (reverse) restriction sites for cloning into the yeast-expression vector pYES2. For cloning of NifD variant sequences within MIT v2.1, silent mutations that created AgeI and SalI sites were introduced into the NifD coding sequence to create MIT v2.1mod. Then, corresponding AgeI/Sal fragments were excised from variant NifD sequences and ligated into the AgeI/SalI sites within MIT v2.1mod. For generation of the NifD–linker–NifK within MIT v2.1, ligase cycling reaction was used to fuse the same linker as used in SN68 between the NifD and NifK subunits. Mutagenesis was used to introduce the Y100Q mutation within this vector.

### Plant Growth and Transient Transformation of *N. benthamiana*.

*N. benthamiana* plants were grown in a Conviron growth chamber at 23 °C under a 16:8-h light:dark cycle with 90 µmol/min light intensity provided by cool white fluorescent lamps. *A. tumefaciens* strain GV3101 (SN vectors) or AGLI (P19 vector) cells were grown to stationary phase at 28 °C in Luria-Bertani (LB) broth supplemented with 50 mg/mL carbenicillin or 50 mg/L kanamycin, according to the selectable marker gene on the vector, and 50 mg/L rifampicin. Acetosyringone was added to the culture to a final concentration of 100 μM, and the culture was then incubated at 28 °C with shaking for another 2.5 h. The bacteria were then pelleted by centrifugation at 5,000 × *g* for 10 min at room temperature. The supernatant was discarded, and the pellet was resuspended in a solution containing 10 mM 2-(*N*-morpholino)ethanesulfonic acid (pH 5.7), 10 mM MgCl_2_, and 100 μM acetosyringone, after which the optical density at a wavelength of 600 nm (OD_600_) was measured. A volume of each culture, including the culture containing the viral-suppressor construct 35S:P19, required to reach a final concentration of OD_600_ = 0.10, was added to a fresh tube. The final volume was made up with the infiltration buffer. Leaves of 5-wk-old plants were then infiltrated with the culture mixture, and the plants were grown for 5 d after infiltration before leaf discs were recovered for analysis.

### Transformation of *A. thaliana*.

SN and SL plasmids were electroporated into *A. tumafaciens* GV3101 cells and plated on 50 µg/mL rifamycin, 25 µg/mL gentamicin, and 50 µg/mL kanamycin. Plates were incubated for 48 h at 28 °C in the dark. Afterward, a single clone was plated out on the same selection for 48 h. A streak of bacteria was then inoculated in a 10-mL liquid LB starter culture containing the same selection regime. After growth at 28 °C overnight with shaking at 200 rpm, 250 µL of this culture was used to inoculate a 250-mL culture of liquid LB. The 250-mL *Agrobacterium* culture was spun down and resuspended in 250 mL of milliQ water with 5% sucrose and 0.03% Silwet reagent (31). Pots containing 50 to 100 *Arabidopsis* plants at flowering were dipped in the medium and wrapped in plastic for 2 d. Plants were then grown to seed set, and seeds were harvested. Seeds were sterilized and placed on LB plates with 50 µg/mL kanamycin to identify primary transformants.

### Yeast Transformation.

Transformation of yeast strain INVSc1 (Thermo Fisher Scientific) was performed by using the Yeast Transformation Kit (Sigma-Aldrich) according to the manufacturer’s protocol. Transformed colonies were selected by plating the transformation mixture onto minimal medium without uracil (SCMM-U) agar plates, which contained 6.7 g/L yeast nitrogen base, 1.92 g/L synthetic dropout medium without uracil (Sigma-Aldrich), 20 g/L glucose, and 20 g/L agar. After 2 to 3 d of incubation at 30 °C, single colonies were restreaked onto fresh SCMM-U agar plates. A single colony that contained the genetic construct was inoculated into SCMM-U liquid medium (containing the same components as SCMM-U agar, but without the agar), grown at 30 °C with shaking for 2 d. Glycerol was added to a final concentration of 20%, and aliquots were stored at −80 °C until further use.

For expression of the genes contained in the genetic construct, an inoculant from the glycerol stock was grown in SCMMM-U liquid medium at 30 °C with shaking for 2 d. The cells were collected from the culture by centrifugation and resuspended in SCMM-U induction medium, which was identical to SCMM-U liquid medium, except that the glucose was replaced with 20 g of galactose, to a final OD_600_ of 0.4. The culture for induction was grown at 30 °C with shaking for 2 d, and the yeast cells were collected by centrifugation for protein extraction and Western blot analysis.

### Plant and Yeast Protein Extractions and Western Blot Analysis.

For analysis of total protein, extractions were performed as described in ref. 12. Yeast protein was extracted by using the same buffer as for *N. benthamiana*, except that ∼100 mg of cells was used with an equivalent volume of extraction buffer. For preparation of soluble and insoluble fractions, from the same infiltrated leaf disk used for total protein, ∼180 mm^2^ of leaf disk was ground under liquid nitrogen by using a mortar and pestle, and 300 µL of cold solubility buffer was added. The solubility buffer contained 50 mM Tris⋅HCl (pH 8.0), 75 mM NaCl, 100 mM mannitol, 2 mM dithiothreitol (DTT), 0.5% (wt/vol) polyvinylpyrrolidone (average molecular weight 40,000), 5% (vol/vol) glycerol, 0.2 mM phenylmethylsuflonyl fluoride, 10 μM leupeptin, and 0.5% (vol/vol) Tween 20. The samples were centrifuged for 5 min at 16,000 × *g* at 4 °C. The supernatant was transferred to a fresh tube, and the pellet was resuspended in 300 µL of cold solubility buffer. Both the supernatant (sample 1) and the resuspended pellet (sample 2) were centrifuged again for 5 min at 16,000 × *g* at 4 °C. From sample 1, a sample was taken from the supernatant, which is referred to as the soluble fraction. This sample was mixed with an equivalent amount of 2× SDS buffer. The 2× SDS buffer contained 250 mM Tris⋅HCl (pH 6.8), 8% (wt/vol) SDS, 40% (vol/vol) glycerol, 120 mM DTT, and 0.004% (wt/vol) bromophenol blue. After the second centrifugation step, the supernatant of sample 2 was discarded. The pellet is referred to as the insoluble fraction. The pellet was resuspended in 300 µL of 4× SDS buffer, and 300 µL of solubility buffer was added. Samples for the total, insoluble, and soluble fractions were heated at 95 °C for 3 min and then centrifuged at 12,000 × *g* for 2 min. A total of 20 µL of the supernatant containing the extracted polypeptides was loaded on a NuPAGE Bis-Tris 4 to 12% gels (Thermo Fisher Scientific) for gel electrophoresis and Western blot analysis, as described by ref. 12. The NifK antibody used in these experiments was donated by Yuichi Fujita, Nagoya University, Nagoya, Japan. It is a polyclonal antibody, which was raised in rabbit against 6×His-NifK recombinant proteins from *Leptolyngbya boryana* and was used at 1:5,000 dilution. The isocitrate dehydrogenase antibody (Agrisera) was used at 1:2,000 dilution.

### Sequence Alignment and Visualization of NifD Sequences.

A total of 1,751 NifD sequences (family IPR005972, nitrogenase molybdenum-iron protein alpha chain) were extracted on 12 December 2018 from the InterPro database (20). Removal of duplicate sequences resulted in a set of 1,476 unique sequences. The sequences were aligned by using the multiple-sequence alignment program MAFFT (version 7) (32). The FFT-NS-2 (fast but rough) strategy was used with default parameters. The aligned sequences were visualized by using ALVIS (interactive nonaggregative visualization and explorative analysis of multiple sequence alignments) (21).

### Acetylene Reduction and ^15^N Assays Using the pMITv2.1 System in *E. coli*.

Cells of *E. coli* strain JM109 were transformed with the plasmids pMIT v2.1 (or one of its derivatives that was being tested) and pN249, which conferred resistance to the antibiotics chloramphenicol and spectinomycin, respectively, as described in ref. 33. The transformed cells were selected by growth on LB medium (10 g/L tryptone, 5 g/L yeast extract, and 10 g/L NaCl) containing chloramphenicol (34 mg/L) and spectinomycin (80 mg/L). Transformed cells were grown aerobically overnight at 37 °C in LB medium with antibiotics to an optical density of 1.0. The cultures were centrifuged at 10,000 × *g* for 1 min, and the supernatant was discarded. The cells were resuspended in one volume of an induction medium which was free of N sources, containing 25 g/L Na_2_HPO_4_, 3 g/L KH_2_PO_4_, 0.25 g/L MgSO_4_0.7H_2_O, 1 g/L NaCl, 0.1 g/L CaCl_2_0.2H_2_O, 2.9 mg/L FeCl_3_, 0.25 mg/L Na_2_MoO_4_0.2H_2_O, and 20 g/L sucrose (minimal medium) supplemented with 1.5 mL/L 10% serine, 600 µl/L 0.5% Casamino acids, 5 mg/L biotin, and 10 mg/L para-aminobenzoic acid (15). For ARAs, the medium was sparged with argon gas for 20 min prior to mixture with the bacteria and antibiotics. Stock solutions were filter-sterilized. For induction of *nif* gene expression, the medium was supplemented with isopropyl-β-d-1-thiogalactopyranoside at a final concentration of 0.1, 0.5, or 1.0 mM, unless otherwise stated, generally 1.0 mM. The cell suspensions were transferred to 3.5-cm^3^ culture flasks and capped with gas-tight rubber seals using a crimp-lock system, and the headspace was sparged with pure argon gas for 20 min. The suspensions were then incubated at 30 °C with shaking at 200 rpm for 5 h. After this, ARAs were started by the injection of 0.5 cm^3^ of pure C_2_H_2_ (BOC gases, instrument grade; final concentration 10% C_2_H_2_ in argon) and further incubation for 18 h. Production of ethylene at the final time was measured by gas chromatography with flame ionization detection (GC-FID) using an Agilent 6890N GC instrument. Headspace samples (0.5 cm^3^) were removed and manually injected into a split/splitless inlet on a 10:1 split mode. The instrument was operated under the following parameters: inlet and FID temperatures of 200 °C, average velocity for the carrier He of 35 cm/s, and isothermal oven temperature at 120 °C. A RT-Alumina Bond/MAPD column (30 m × 0.32 mm × 5 µm) was used with a 5-m particle trap column coupled to the detector end. Analytical performance of the instrument was assessed by running suitable blanks and standards. Under these conditions, ethylene emitted from the column at about 2.3 min and acetylene at about 3.1 min. For ^15^N measurements, the cells were grown and resuspended in the induction medium as described for the ARAs. The tubes were sparged with nitrogen gas for 20 min and induced at 30 °C with shaking at 200 rpm for 5 h. After induction, 0.5 mL of ^15^N gas (Sigma-Aldrich; 98% atom) was injected into the 3.5-cm^3^ vial and incubated at 30 °C with shaking at 200 rpm for 48 h. After incubation, 100 μL of cells from each tube was freeze-dried in a tin capsule with inert chromosorb for 48 h in a desiccator at room temperature, weighed, and analyzed by using a SerCon hydra 20-20 isotope ratio mass spectrometer. δ^15^N values were calculated as per the method of Montoya et al. (34).

## Supplementary Material

Supplementary File

Supplementary File

## Data Availability

All study data are included in the article and *SI Appendix*.
