# Supplementary material for: Plant expression of NifD protein variants resistant to mitochondrial degradation
Source: Proc Natl Acad Sci U S A. 2020 Aug 31;117(37):23165–73. doi: 10.1073/pnas.2002365117 (PMC7502725; doi:10.1073/pnas.2002365117)
Supplement: Supplementary File [file pnas.2002365117.sapp.pdf]

Supplementary Information for

Plant expression of NifD protein variants resistant to mitochondrial degradation

Robert S. Allen (1, #), Christina M. Gregg (1, #) , Shoko Okada (2), Amratha Menon (1), Dawar Hussain, (1), Vanessa Gillespie (1), Ema Johnston (1), Andrew C. Warden (2), Rosangela Devilla (1), Matthew Taylor, (2), Michelle Colgrave (3,4), Keren Byrne (3), Craig C. Wood (1,*)

#These authors made equal contributions.*Corresponding author

Affiliations:

1) Agriculture and Food, Commonwealth Scientific and Industrial Research Organisation, Acton, Canberra, ACT 2600, Australia

2) Land and Water, Commonwealth Scientific and Industrial Research Organisation, Acton, Canberra, ACT 2600, Australia

3) Agriculture and Food, Commonwealth Scientific and Industrial Research Organisation, Queensland Biosciences Precinct, St Lucia, Brisbane, QLD 4067, Australia

4) Australian Research Council Centre of Excellence for Innovations in Peptide and Protein Science, Queensland Biosciences Precinct, St Lucia, Brisbane, QLD 4067, Australia

www.pnas.org/cgi/doi/10.1073/pnas.2002365117

Contents

Supplementary Materials and Methods

Supplementary Figures

Figure S1: Representative genetic maps for plant expression constructs.

Figure S2: The NifD degradation product is present in both the presence and absence of NifK.

Figure S3: Removal of downstream NifD *ATG* codons does not prevent accumulation of a degradation product.

Figure S4: Visualisation of mitochondrial cleavage sites

Figure S5: MTP::NifD(Y100Q) is resistant to degradation in the absence of NifK

Figure S6: Mitochondrial pull-down confirming the mitochondrial location of MTP::NifD proteins.

Figure S7: Details of the modifications made to MIT v2.1 for assessing NifD variants.

Figure S8: Solubility of NifD in different environments

Figure S9: Genetic maps of yeast expression constructs 6xHis::NifD::HA, FAγ51::NiD::HA and alaFAγ51::NifD::HA.

Figure S10: Sequence diversity of NifD proteins.

Figure S11: Mitochondrial processing of NifD proteins from different organisms.

Supplementary Tables

Table S1: List of all constructs for expression of genes in plants.

Table S2: Protein sequences for Nif constructs used in this study

Table S3: Frequency distribution of amino acid residues in 1476 putative NifD sequences around the secondary cleavage site.

Table S4: List of sequences that were used for the visualisation of mitochondrial cleavage sites.

Table S5: Sizes for unprocessed and processed NifD and the predicted size of the C-terminal degradation product arising from secondary cleavage by the MPP.

Supplementary Dataset 1

Supplementary References

Supplementary Materials and Methods

Identification of the NifD cleavage product by mass spectrometry

Protein extracts from N. benthamiana leaves infiltrated with SN14 (MTP-Su9::NifD::HA) were run on SDS-PAGE using a gel having a polyacrylamide concentration of 4-20 % (Invitrogen). The gel was stained with Aqua stain (Bulldog Bio). After destaining in water, 5 slices were cut from the gel for the region spanning the molecular weights 37-50 kDa. The slices were numbered 1 to 5 from the smaller molecular weights to the larger. Each gel slice was cut into approximately 1 mm3 cubes and soaked in 150 µl 30% methanol for 15 minutes. To reduce proteins that may have oxidised, the buffer was removed and replaced with 100 µl of fresh 25 mM ammonium bicarbonate (ABC) buffer with 5 µl of 15% dithiothreitol and incubated at room temperature for an hour. Cysteine residues were inactivated by the addition of 5 µl of 40% acrylamide and incubation at room temperature for 1 hour, after which the buffers were carefully removed. Three wash steps were carried out, each of 50 µl of ABC buffer and 50 µl acetonitrile and incubation at room temperature. The gel pieces were dried by the addition of 100 µl of 100% acetonitrile for 2 min, which was then discarded. The proteins in the dried gel pieces were then digested with 0.1 µg trypsin (Promega) in 20 µl ABC with incubation overnight at 37°C. The tryptic digest was stopped with 1 µl of a 50% (v/v) formic acid solution and sonication for 15 min. The samples were filtered after the addition of 10 µl of water before transfer into LCMS vials.

The resulting tryptic digest from each gel slice was injected onto a Dionex Nanomate 3000 (ThermoFisher) nano liquid chromatography (LC) system directly coupled to an Orbitrap Fusion Tribrid Mass Spectrometer. The peptides were desalted for 5 min on an Acclaim PepMap C18 (300 Å, 5 mm x 300 µm) trap column at a flow rate of 10 µL/min with loading solvent, and separated on an Acclaim PepMap C18 (100 Å, 150 mm × 0.075 mm) column at a flow rate of 0.3 µL/min at 35°C. A linear gradient from 5% to 40% solvent B over 60 min was employed followed by a wash and re-equilibration with 40−99% B over 5 min, a 5 min hold at 99% B, return to 5% B over 6 min, and held for 7 min. The solvents used were: (A) 0.1% formic acid, 99.9% water; (B) 0.08% formic acid, 80% acetonitrile, 19.92% water. The nano-LC was directly coupled to the Nanospray Flex Ion source of the Orbitrap Fusion MS. The ion spray voltage was set to 2400 V, the sweep gas was set to 1 Arb and the ion transfer tube temperature was set to 300°C. Data were acquired in data-dependent acquisition mode consisting of a Orbitap-MS survey scan followed by parallel acquisition of a high resolution

Orbitrap scan at 120,000 resolution and multiple MS/MS events in the linear ion trap, over a 3 second period. First stage MS analysis was performed in positive ion mode over the mass range of m/z 400−1500 with an AGC target of 4 x 105 and a maximum injection time of 50 ms. Tandem mass spectra were acquired in the ion trap on precursor ions that exceeded an intensity threshold of 1000 counts with charge state 2−7. Spectra were acquired using quadrupole isolation with a 1.6 m/z isolation window and (Higher energy Collisional Dissociation) HCD set at 28% based on the size and charge of the precursor ion for optimum peptide fragmentation. Ion trap scan rate was set to rapid with an AGC target of 4 x 103 and a maximum injection time of 300 ms, the instrument was set to utilise the maximum parallelizable time for injecting ions into the trap during a 3 second window whilst the orbitrap was collecting high resolution MS spectra. Dynamic exclusion was set to exclude precursor ions after one occurrence with a 15 second interval and a mass tolerance of 10 ppm.

Analysis of the data for protein identification was conducted using the Sequest algorithm in Proteome Discoverer v2.2 (ThermoFisher). Carbamidomethyl was selected as the alkylating agent and trypsin was selected as the digestion enzyme. Dynamic modifications were selected for oxidation on NifD with a maximum of three modifications. Tandem mass spectrometry data were searched against a database of tryptic peptides for NifD derived from the polyprotein amino acid sequence encoded by SN14 and the N. benthamiana proteome, common contaminants and organism specific databases annotated from UniProt. The database search results were curated to yield the protein identifications using a 1% global false discovery rate (FDR) determined by the in-built FDR tool within Proteome Discoverer software.

Data extraction and visualisation of mitochondrial cleavage sites

The data for the visualisation of the mitochondrial cleavage sites was extracted from studies by Carrie et al. (2015) and Huang et al. (2009) (1, 2). From the set of N-terminal peptides identified by Carrie et al. sequences that did not contain a pre-sequence, potential substrates of OCT1 and potential dually targeted proteins were excluded. If several peptides were identified for the same protein, the sequence with the cleavage site closest to the N-terminal was used. Sequences were aligned relative to their cleavage site and the alignment was visualised using ALVIS (3). The list of proteins used can be found in Table S4.

Distance matrix calculation and network visualisation

The PHYLIP/protdist program was used to calculate the Kimura distance matrix for the NifD sequences (4). To create subgroups, all values greater than 0.25 were removed. The distance matrix was then imported into Cytoscape using the aMATReader app as an undirected network (5, 6). At this stage the network contained 1474 nodes and 86043 edges. The network was visualized using the prefuse force directed layout (unweighted). Additional information was extracted from the UniProt knowledgebase (entry name, status, protein names, gene names, organism, length and taxonomic lineage) (7) and imported into Cytoscape. Nodes were coloured by phylum.

Metaxin-based pull-down of mitochondria from *N. benthamiana*

Leaves of *N. benthamiana* were co-infiltrated with Twin-strep::mTurquoise::metaxin, MTP::NifK and a NifD construct. The mitochondrial purification was performed in a cold room at 4°C and suspensions containing the organelles were handled with cut tips. Approximately 5 cm^2 of leaf was ground in 1 mL cold KPBS buffer (136 mM KCl, 10 mM KH_2PO_4, pH 7.25) with a mortar and pestle. From this suspension, a sample for the total extract was taken for western blot analysis. The suspension was centrifuged at 1000 x g for 10 min at 4°C. A sample of the supernatant was taken for western blot analysis (input). The remaining supernatant was transferred to 100 µL magnetic beads coated with streptavidin (Dynabeads™ MyOne™ Streptavidin C1), which had been washed two times with 1 mL KPBS buffer. The supernatant and the magnetic beads were incubated for 5 min while rotating. The magnetic beads were pulled down, washed with 1 mL KPBS buffer and resuspended. This washing procedure was carried out three times. The magnetic beads were pulled down again, resuspended in 80 µl Laemmli buffer and heated at 95°C for 5 min to release the proteins. 15 µl of each sample were loaded on the gel for western blot analysis.

Supplementary Figures

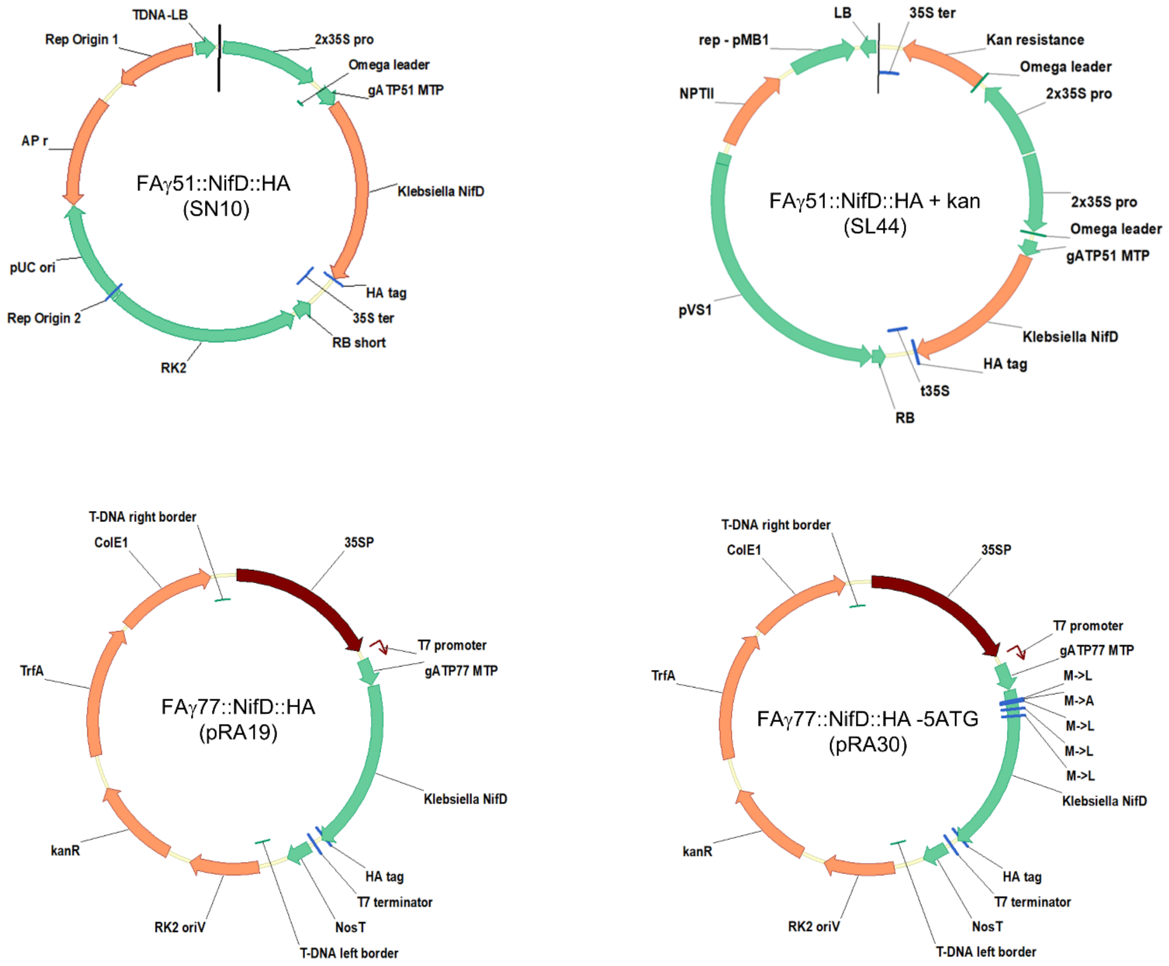

Figure S1. Representative genetic maps for plant expression constructs. GoldenGate single gene vector (SN10), GoldenGate multigene vector (SL44), pRA19 and pRA30. For a full list of all constructs refer to Table S1.

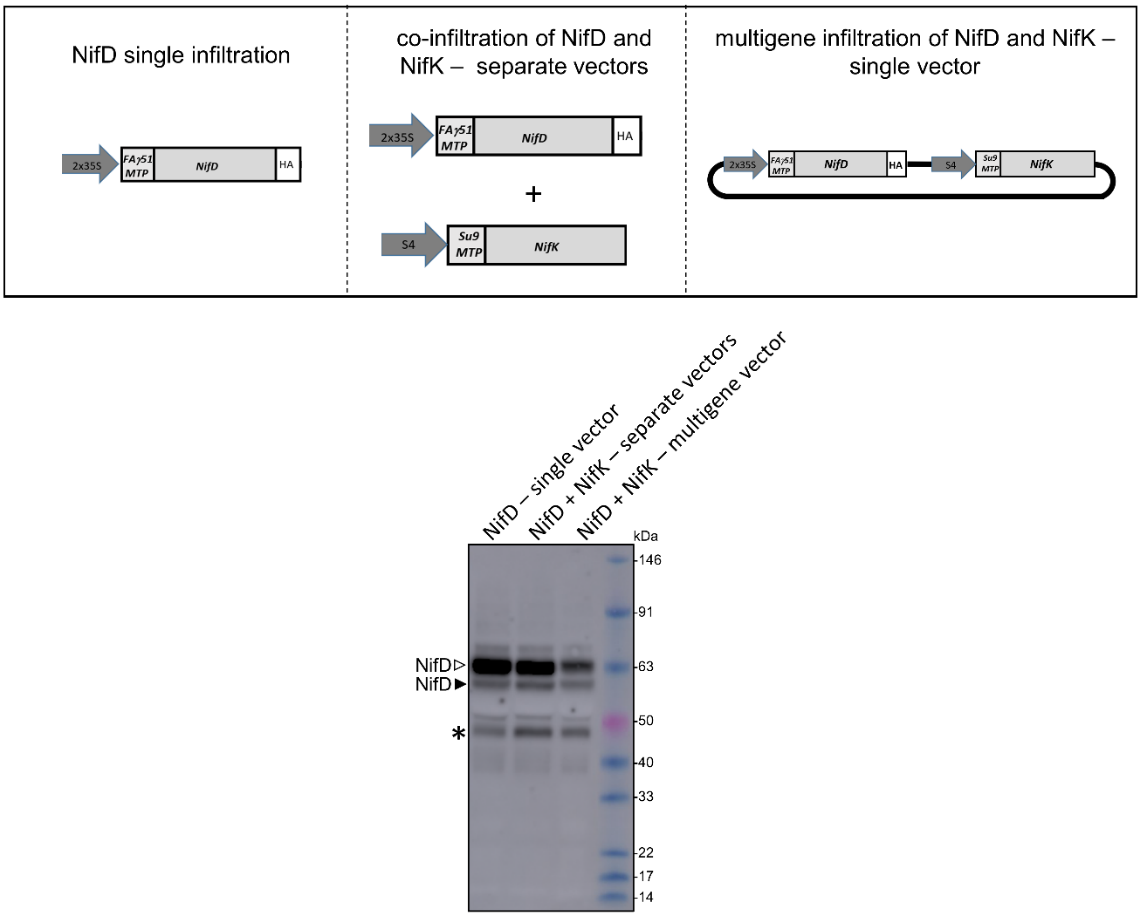

Figure S2. The NifD degradation product is present in both the presence and absence of NifK. Comparison of the accumulation of the degradation product when MTP::NifD vectors were infiltrated in *N.benthamiana* either without NifK, co-infiltrated with NifK on separate vectors, or infiltrated as a multigene construct containing both NifD and NifK on a single vector. The proteins were extracted and analysed by wetern blot using α-HA. The NifD degradation product accumulated regardless of whether NifD and NifK were co-infiltrated on separate vectors or on a single multigene vector. Unprocessed and processed NifD is marked by an empty and solid triangle, respectively, and the degradation product by an asterisk.

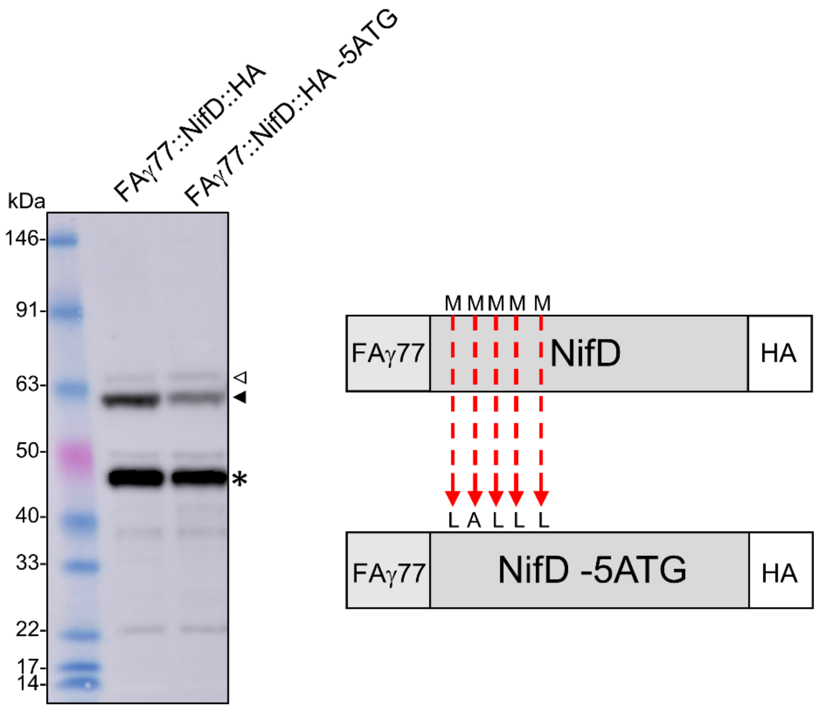

Figure S3. Removal of downstream NifD *ATG* codons does not prevent accumulation of the degradation product. A construct based on FAγ77::NifD::HA (pRA19) (8) was modified by replacing the five methionine codons downstream of the start codon with alternate codons (coding amino acids L, A, L, L, L) to create FAγ77::NifD::HA-5ATG (pRA30). FAγ77::NifD::HA and FAγ77::NifD::HA-5ATG were co-infiltrated with NifK (FAγ77::NifK, pRA25) into *N. benthamiana*, the proteins were extracted, and western blotting was carried out using α-HA. Unprocessed and processed NifD is marked by an empty and solid triangle, respectively, and the degradation product by an asterisk.

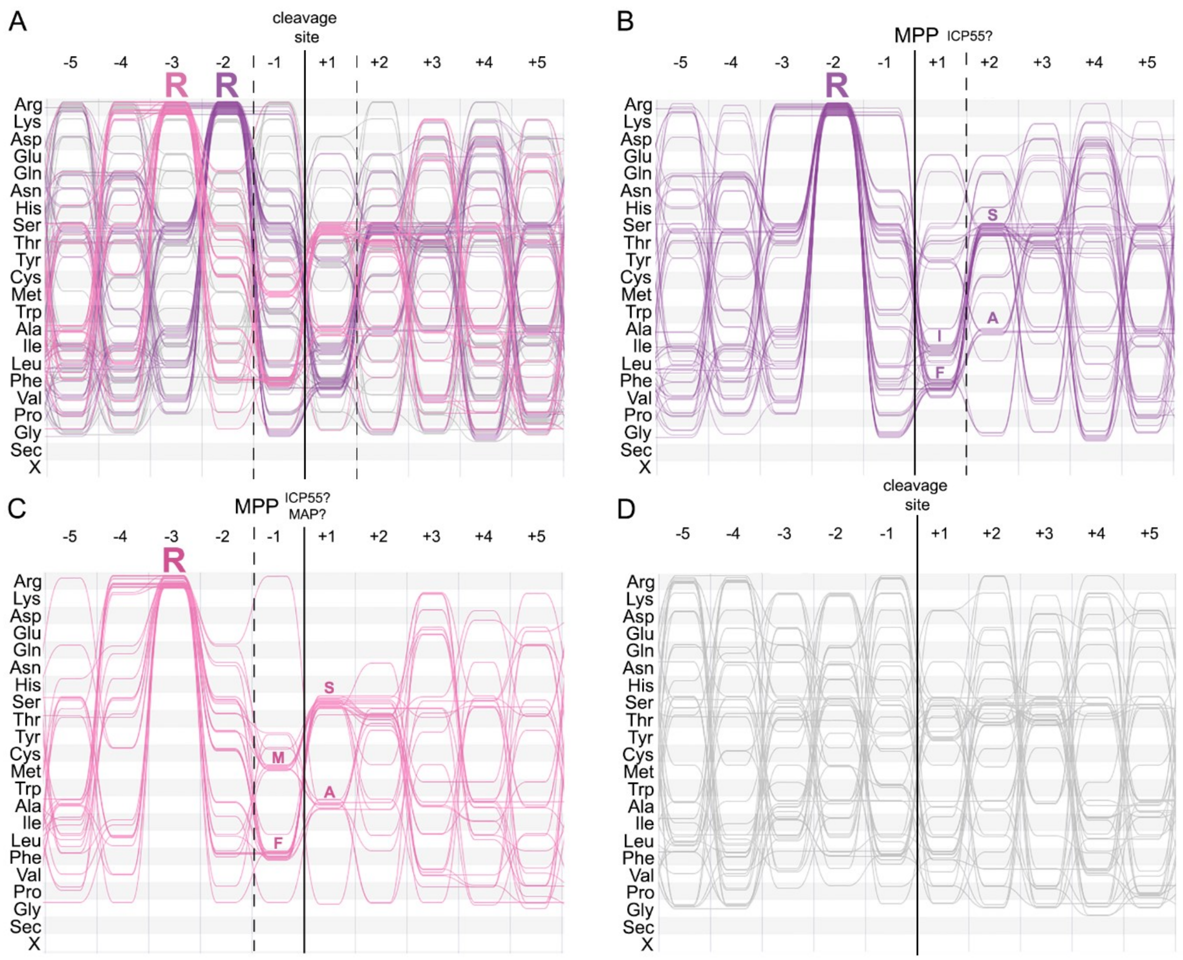

Figure S4. Visualisation of mitochondrial cleavage sites. **A** ALVIS visualisation of mitochondrial cleavage sites from Δ*icp55* and wildtype *Arabidopsis thaliana,* previously identified by Carrie et al. (2015) and Huang et al. (2009) (1, 2). Sequences containing an Arg residue in position -2 or -3 are shown in purple and pink, respectively. Sequences containing no conserved Arg residue are shown in grey. Of the 140 sequences in this alignment, 65 belong to the -2R group, 30 to the -3R group, and 45 contain no conserved Arg residue. **B** Alignment of -2R proteins, which are cleaved by the MPP at the indicated site. As some cleavage sites were identified in an Δ*icp55* background, some proteins contain an additional ICP55 cleavage site between position +1 and +2. **C** Alignment of -3R proteins. The MPP cleavage site is most likely located between position -1 and -2. Cleavage by the MPP is then followed by the removal of one further amino acid. ICP55 has been shown to remove Phe, Tyr and Leu residues (1) that make up 50%, 6.6% and 3.3%, respectively, of the -3R proteins shown here. Met residues (26.6%) could potentially be removed by a methionine aminopeptidase (MAP) (9). **D** Sequences containing no conserved Arg residue in position -2 or -3. For a full list of protein sequences used in this alignment refer to table S3.

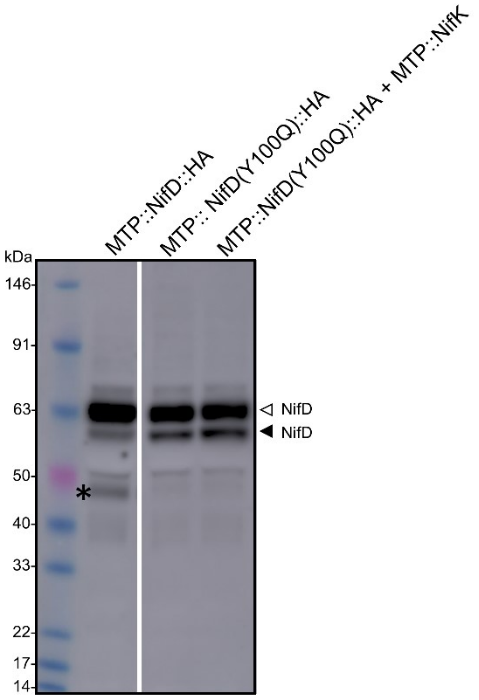

Figure S5: MTP::NifD(Y100Q) is resistant to degradation in the absence of NifK. Comparison of degradation resistance of MTP::NifD-Y100Q in the presence and absence of MTP::NifK. After *N. benthamiana* infiltration, total protein was extracted and subject to western blot analysis using α-HA. Unprocessed and processed NifD is marked by an empty and solid triangle, respectively, and the degradation product by an asterisk.

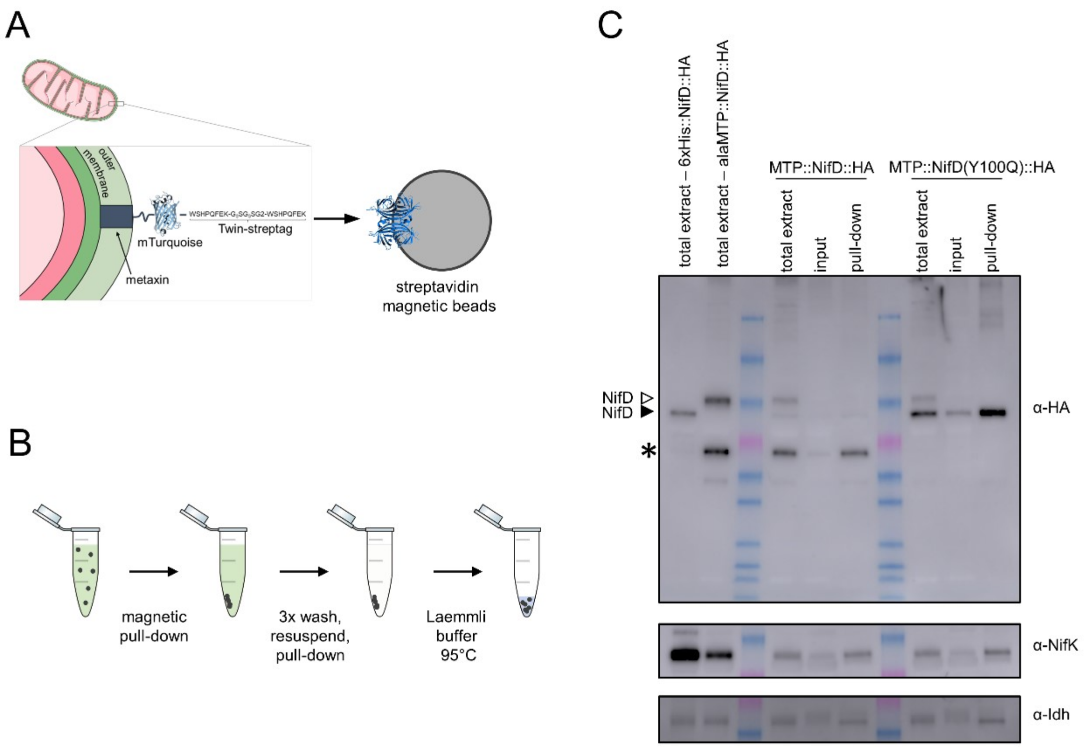

Figure S6: : Mitochondrial pull-down confirming the mitochondrial location of MTP::NifD proteins. **A** Principle of the metaxin-based mitochondrial pull-down. Metaxin is a mitochondrial outer membrane protein with an N-terminal Cytosolic domain and a C-terminal membrane domain. We designed a fusion protein of metaxin that is linked, at its N-terminus, to Twin-streptag::mTurquoise. We were confident that this design would not disrupt the targeting of metaxin to the outer membrane, as the fusion protein GFP-metaxin was previously shown to be targeted to the mitochondrial outer membrane (10). The Twin-streptag exposed to the cytosol allows the capture of mitochondria using magnetic beads coated with streptavidin. **B** Procedure of the small-scale mitochondrial purification. **C** Western blot analysis of metaxin-based mitochondrial pull-down of plant leaves expressing MTP::NifD::HA and MTP::NifD-Y100Q::HA, both co-expressed with MTP::NifK. The first two lanes contain two size controls: 1) Cytosolic NifD, 6xHis::NifD::HA, mimicking the processed size of NifD (solid triangle) and 2) alaMTP::NifD::HA, mimicking the unprocessed size (empty triangle). AlaMTP::NifD::HA is imported into the mitochondria, but not cleaved at the canonical MTP cleavage site. However, it is still cleaved at the secondary RRNYY site, and thus also shows a band for the degradation product (asterisk). In the total extract of leaves expressing MTP::NifD::HA, all three forms of NifD are present: unprocessed NifD, processed NifD, and the degradation product. Compared to the total extract, the concentration of NifD is greatly reduced in the input sample. Compared to the input sample, the pull-down sample shows clear

enrichment of the degradation product, confirming that the degradation product is accumulating in mitochondria. In the total extract of MTP::NifD-Y100Q, only the forms for unprocessed and processed NifD are present. Again, the concentration of NifD is greatly reduced in the input sample. The sample from the mitochondrial pull-down shows an enrichment of correctly processed NifD and no degradation product, confirming that MTP::NifD-Y100Q was imported into mitochondria, but not degraded. The lower panels show the blot probed with α-NifK and α-isocitrate dehydrogenase (α-Idh) antibodies. Idh is a mitochondrial matrix protein. Its enrichment in the pull-down samples compared to the input samples shows that we were able to enrich mitochondria using the metaxin-based pull-down. The anti-Idh antibody was applied to the same membrane as the anti-NifK antibody without stripping the membrane.

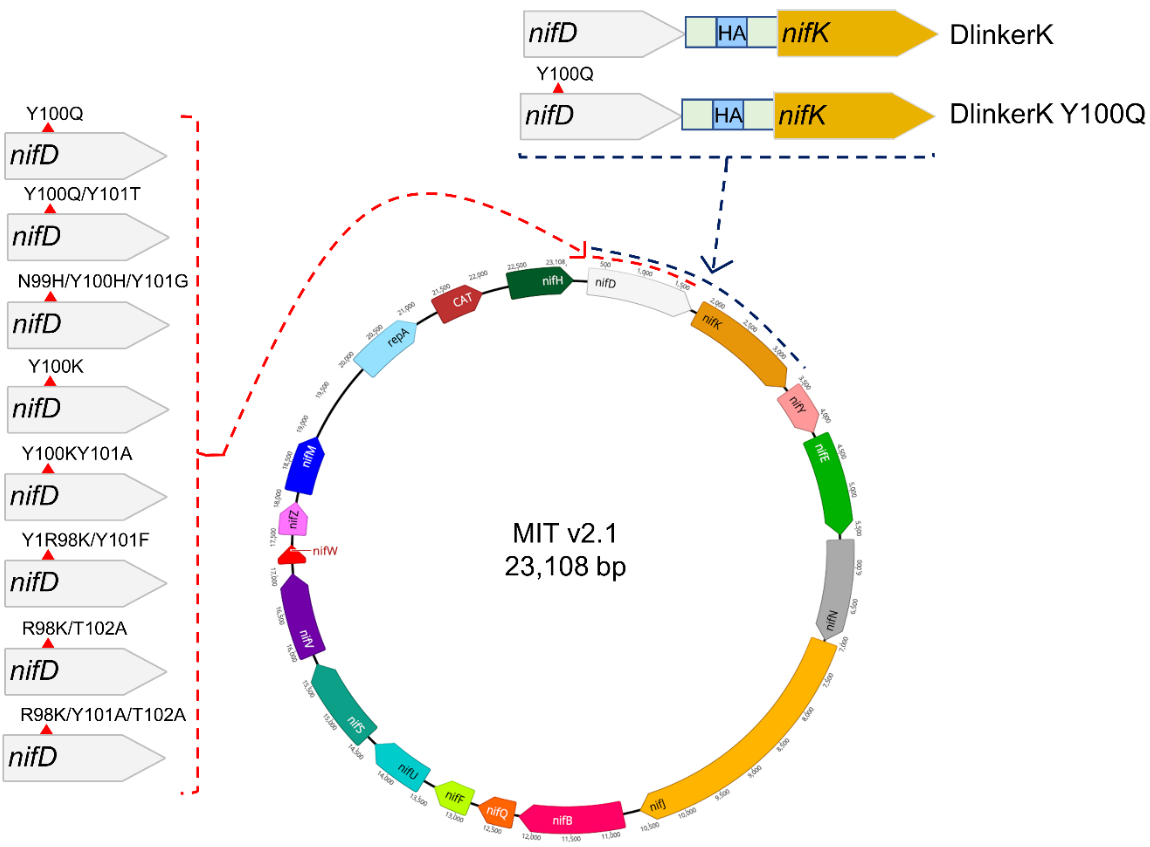

Figure S7: Details of the modifications made to MIT v2.1 for assessing NifD variants. The arrangement of genes within MIT v2.1 is shown to scale. For individual NifD variants single or multiple amino acid substitutions were incorporated within the NifD sequence as shown (red dashed lines). To test the NifD::linker::NifK protein, the linker replaced the region between the NifD and NifK subunits and for the NifD(Y100Q)::linker::NifK protein, the Y100Q mutation was also included (dark blue dashed lines).

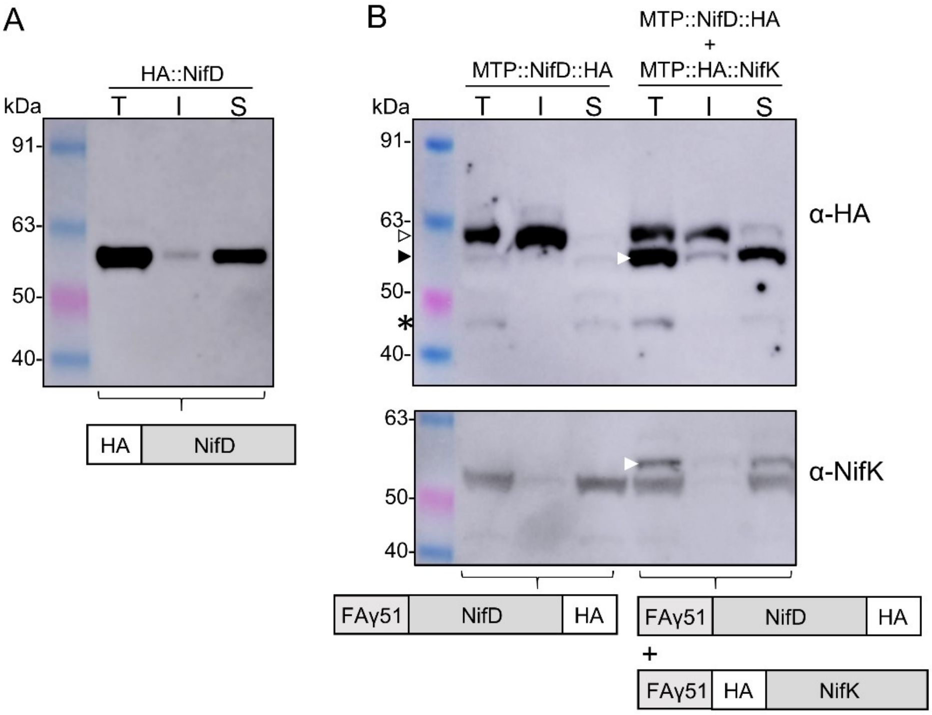

Figure S8. Solubility of NifD in different environments. **A** Cytosolic HA::NifD accumulates mostly in the soluble fraction. After *N. benthamiana* infiltration, protein was separated into total (T), insoluble (I) and soluble (S) fractions and subjected to western blot analysis using α-HA. No degradation product was observed and most HA::NifD accumulated in the soluble fraction. **B** The presence of NifK does not increase the solubility of MTP::NifD. MTP::NifD was infiltrated either separately or co-infiltrated with MTP::HA::NifK. Protein extracts were separated into total (T), insoluble (I) and soluble (S) fractions. Western blot analysis using α-HA and α-NifK were used to discriminate the proteins in each fraction, as processed NifK (white solid triangle) runs at the same size as processed NifD. The NifK antibody cross-reacts with the total and soluble fractions, the specific band size is indicated by a white triangle. Unprocessed and processed NifD is marked by an empty and solid triangle, respectively, and the degradation product by an asterisk.

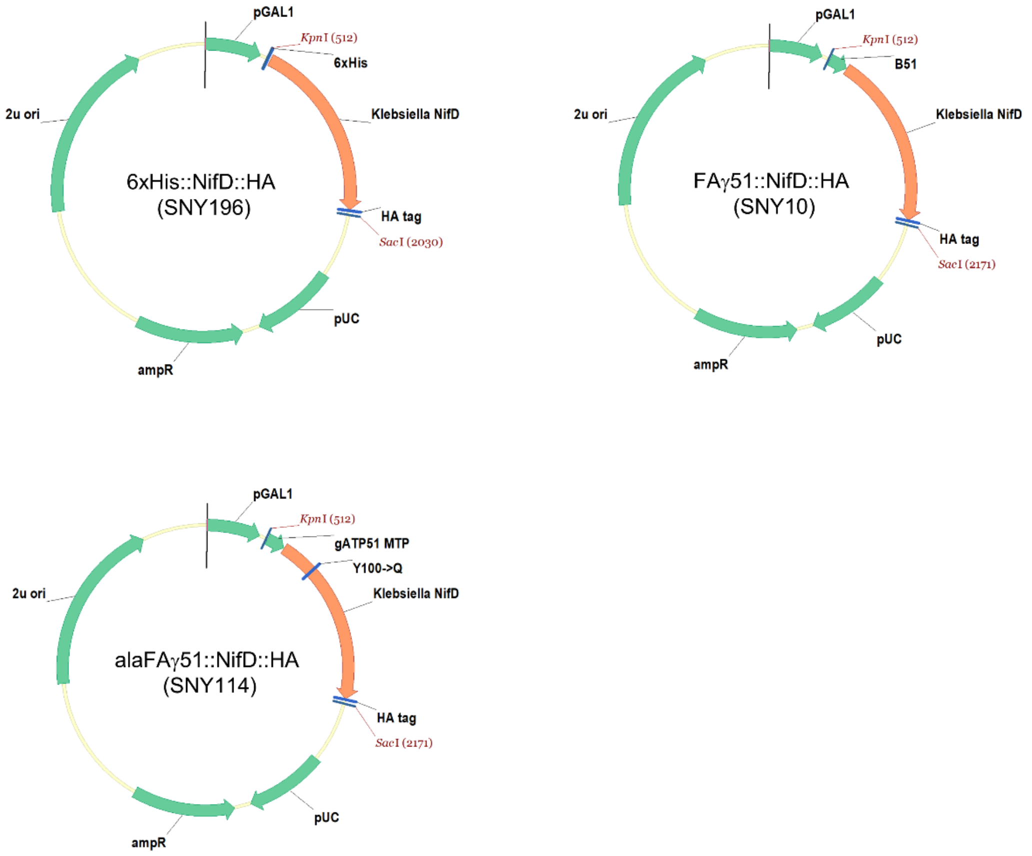

Figure S9: Genetic maps of yeast expression constructs 6xHis::NifD::HA, FAγ51::NiD::HA and alaFAγ51::NifD::HA.

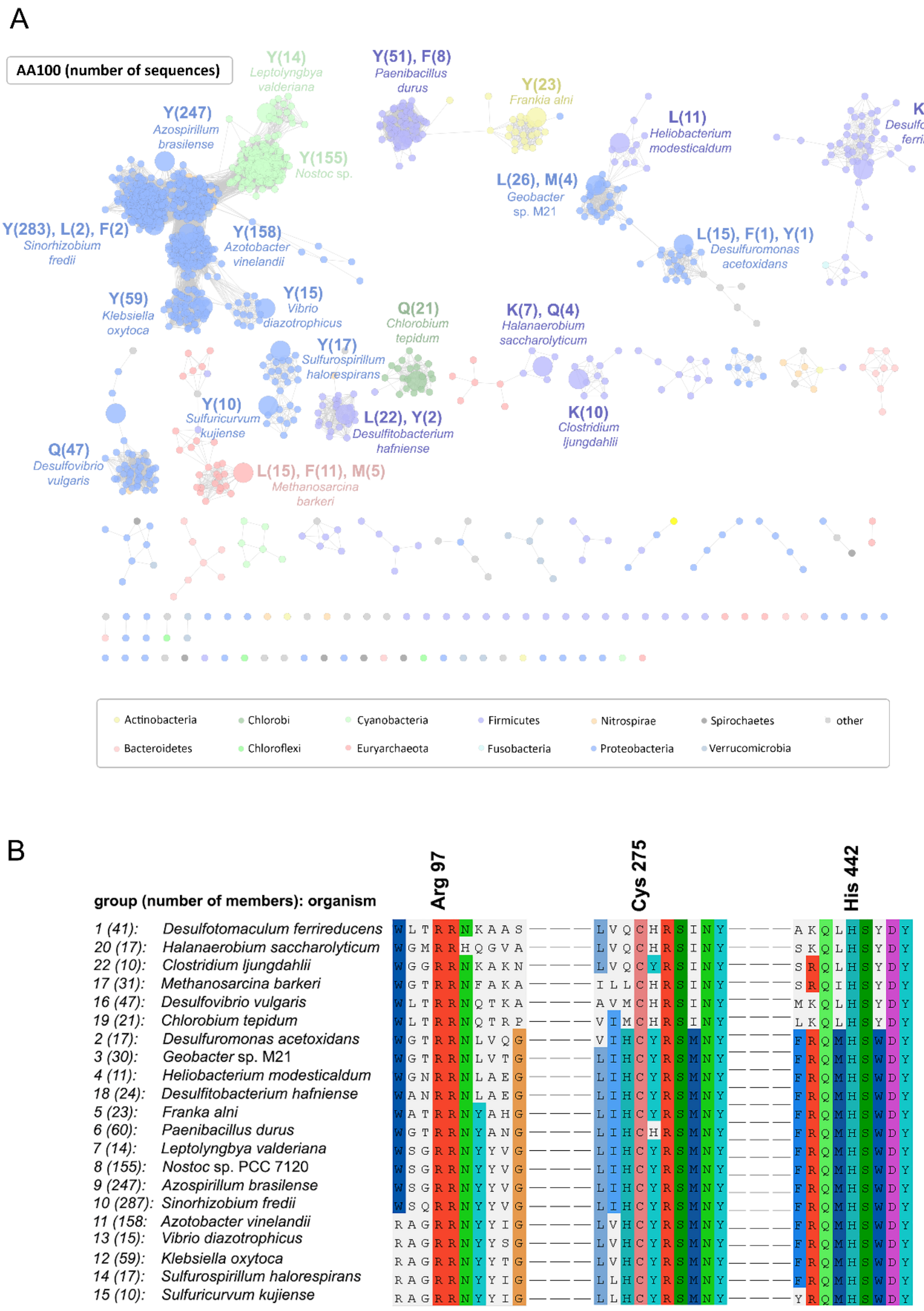

Figure S10. Sequence diversity of NifD proteins**. A** Sequence similarity network of putative NifD sequences and distribution of the amino acid that is equivalent to Y100 in *K. oxytoca* across different clusters. Nodes (circles) represent protein sequences and edges (grey lines) connect two sequences that have a Kimura distance below 0.25. Protein sequences of different

phyla are displayed in different colours as indicated by the legend below. The NifD sequences can be divided into 21 clusters with at least 10 members. The one-letter amino acid code displayed for each cluster shows how many sequences within this cluster contain this amino acid in the equivalent position of Y100 in *K. oxytoca.* For each cluster a representative sequence was chosen, displayed by a larger node. **B** Sequence alignment of representative NifD proteins. The sequence alignment shows the amino acid residues around the secondary cleavage site, and the FeMoco ligands, Cys 275 and His 442. The amino acid numbering is based on the sequence of *K. oxytoca*.

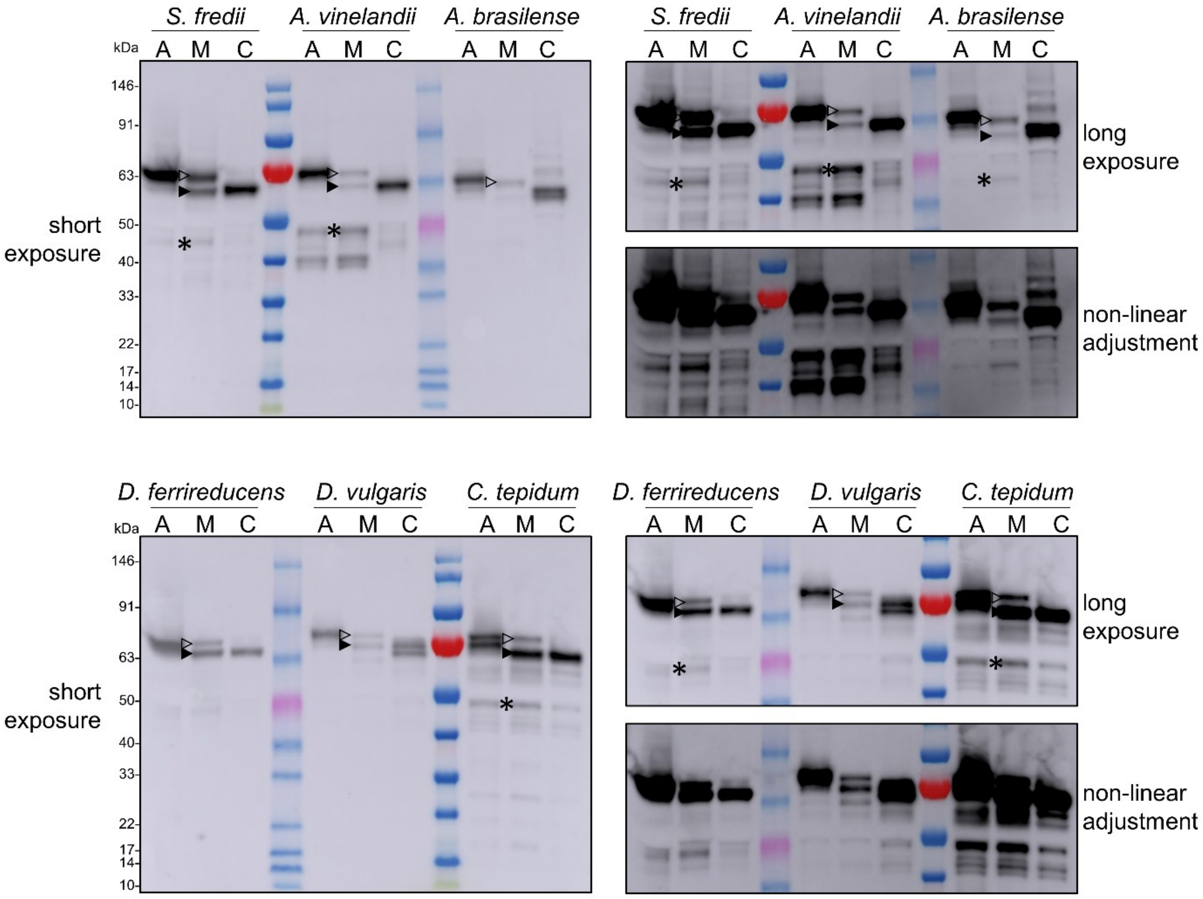

Figure S11. Mitochondrial processing of NifD proteins from different organisms. Western blot (α-HA) to assess mitochondrial processing of NifD proteins from *Azotobacter vinelandii*, *Azospirillum brasilense*, *Chlorobium tepidum*, *Desulfotomaculum ferrireducens*, *Desulfovibrio vulgaris* and *Sinorhizobium fredii* in *N. benthamiana*. Three constructs were analysed for each NifD protein: alaMTP::NifD::HA, which cannot be cleaved by the MPP at the canonical MPP cleavage site (A), MTP::NifD::HA, which is targeted to mitochondria (M), and 6xHis::NifD::HA, which is located in the cytosol (C). The size of unprocessed and processed MTP-::NifD::HA is indicated by empty and solid triangles respectively, and the size of the C-terminal degradation product is indicated by an asterix. The predicted molecular weights of unprocessed and processed NifD proteins, and the C-terminal degradation product are shown in Table S5.

Supplementary Tables

Table S1: List of all constructs for expression of genes in plants. For detailed description of protein coding sequences and DNA parts, refer to Table S2, Dataset S1.

**Key protein features**	**Short name ***	**Description****	**construct number[#]**
CPN60::NifD::HA		NifD with C-terminal HA-tag targeted to mitochondria using MTP CPN60	SN4
S4i - FAγ51::NifD::HA		NifD with C-terminal HA-tag targeted to mitochondria using MTP FAγ51 with S4i promotor	SN6
S4ii - FAγ51::NifD::HA		NifD with C-terminal HA-tag targeted to mitochondria using MTP FAγ51 with S4ii promotor	SN7
S7 - FAγ51::NifD::HA		NifD with C-terminal HA-tag targeted to mitochondria using MTP FAγ51 with S7 promotor	SN8
35SL - FAγ51::NifD::HA		NifD with C-terminal HA-tag targeted to mitochondria using MTP FAγ51 with 35SL promotor	SN9
FAγ51::NifD::HA	MTP::NifD	NifD with C-terminal HA-tag targeted to mitochondria using MTP FAγ51	SN10
CPN60g::NifD::HA		NifD with C-terminal HA-tag targeted to mitochondria using MTP CPN60 with no GG linker between the MTP and NifD	SN11
SOD::NifD::HA		NifD with C-terminal HA-tag targeted to mitochondria using MTP SOD	SN13
Su9::NifD::HA		NifD with C-terminal HA-tag targeted to mitochondria using Su9 MTP	SN14
Kan + FAγ51::NifD::HA		NifD with C-terminal HA-tag targeted to mitochondria using MTP FAy51 (SN10) on a multigene construct with a kanamycin resistance gene as a selection marker for stable transformation	SL44
HA::NifD		NifD with an N-terminal HA-tag, expected to be located in the cytosol	SN33
HA::NifD::HA		NifD with an N- and C-terminal HA-tag, expected to be located in the cytosol	SN34
FAγ51::HA::NifD::HA		NifD with an N- and C-terminal HA-tag, targeted to mitochondria using MTP FAγ51	SN75
FAγ51::NifD(49-53 AA)::HA	NifD-Var1	FAy51::NifD::HA with residues 49-53 replaced by alanine residues	SN52
FAγ51::NifD::HA 54-58 AA	NifD-Var2	FAy51::NifD::HA with residues 54-58 replaced by alanine residues	SN53
FAγ51::NifD::HA 59-63 AA	NifD-Var3	FAy51::NifD::HA with residues 59-63 replaced by alanine residues	SN54
FAγ51::NifD::HA 64-68 AA	NifD-Var4	FAy51::NifD::HA with residues 64-68 replaced by alanine/glycine residues	SN55
FAγ51::NifD::HA 69-73 AA	NifD-Var5	FAy51::NifD::HA with residues 69-73 replaced by alanine residues	SN56
FAγ51::NifD::HA 74-78 AA	NifD-Var6	FAy51::NifD::HA with residues 74-78 replaced by alanine residues	SN57
FAγ51::NifD::HA 79-83 AA	NifD-Var7	FAy51::NifD::HA with residues 79-83 replaced by alanine/glycine residues	SN96
FAγ51::NifD::HA 84-88 AA	NifD-Var8	FAy51::NifD::HA with residues 84-88 replaced by alanine residues	SN97
FAγ51::NifD::HA 89-93 AA	NifD-Var9	FAy51::NifD::HA with residues 89-93 replaced by alanine residues	SN98
FAγ51::NifD::HA 94-98 AA	NifD-Var10	FAy51::NifD::HA with residues 94-98 replaced by alanine/glycine residues	SN99
FAγ51::NifD::HA 99-103 AA	NifD-Var11	FAy51::NifD::HA with residues 99-103 replaced by alanine residues	SN100
FAγ51::NifD::HA 104-108 AA	NifD-Var12	FAy51::NifD::HA with residues 104-108 replaced by alanine residues	SN101
FAγ51::NifD::HA Y101F		FAy51::NifD::HA variant	SN108
FAγ51::NifD::HA T102A		FAy51::NifD::HA variant	SN109
FAγ51::NifD::HA Y101F/T102A		FAy51::NifD::HA variant	SN110
FAγ51::NifD::HA T102A/A103A		FAy51::NifD::HA variant	SN111
FAγ51::NifD::HA R98A		FAy51::NifD::HA variant	SN112
FAγ51::NifD::HA N99H		FAy51::NifD::HA variant	SN113
FAγ51::NifD::HA Y100Q		FAy51::NifD::HA variant	SN114
FAγ51::NifD::HA Y101T		FAy51::NifD::HA variant	SN115
FAγ51::NifD::HA T102V		FAy51::NifD::HA variant	SN116

FAγ51::NifD::HA Y100Q/Y101T		FAγ51::NifD::HA variant	SN117
FAγ51::NifD::HA N99H/Y100K/Y101G		FAγ51::NifD::HA variant	SN118
FAγ51::NifD::HA Y100K		FAγ51::NifD::HA variant	SN119
FAγ51::NifD::HA Y100K/Y101A		FAγ51::NifD::HA variant	SN120
FAγ51::NifD::HA Y101A		FAγ51::NifD::HA variant	SN121
FAγ51::NifD::HA R98K		FAγ51::NifD::HA variant	SN122
FAγ51::NifD::HA R98K/Y101F		FAγ51::NifD::HA variant	SN123
FAγ51::NifD::HA R98K/T102A		FAγ51::NifD::HA variant	SN124
FAγ51::NifD::HA R98K/Y101F/T102A		FAγ51::NifD::HA variant	SN125
FAγ51::NifD::HA R98K/Y101A/T102A		FAγ51::NifD::HA variant	SN126
FAγ51::NifD-linker(HA)-NifK		MTP::NifD-NifK fusion protein, with linker containing a HA tag.	SN68
FAγ51::NifD-linker(HA)-NifK Y100Q		MTP::NifD-NifK fusion protein, including the Y100Q variant, with linker containing a HA tag	SN159
ala-FAγ51::NifD-linker(HA)-NifK Y100Q		NifD-NifK fusion protein, with the Y100Q variant, with linker containing a HA tag, the MTP has been alanine scanned to prevent mitochondrial processing	SN160
6xHis::NifD-linker(HA)-NifK		NifD-linker(HA)-NifK with an N-terminal 6xHis-tag, expected to be located in the cytosol	SN176
6xHis::NifD::HA		NifD with an N-terminal 6xHis-tag, expected to be located in the cytosol	SN196
Su9::NifK	MTP::NifK	Su9::NifK	SN46
Su9::NifK for L2	MTP::NifK	Identical to SN46, but in position 3 L1 vector	SN369
FAγ51::HA::NifK	MTP::HA::NifK		SN140
FAγ51::*Df*NifD::HA		*D. ferrireducens* NifD with C-terminal HA-tag targeted to mitochondria using MTP FAγ51	SN221
FAγ51::*Sf*NifD::HA		*S. fredii* NifD with C-terminal HA-tag targeted to mitochondria using MTP FAγ51	SN222
FAγ51::*Av*NifD::HA		*A. vinelandii* NifD with C-terminal HA-tag targeted to mitochondria using MTP FAγ51	SN223
FAγ51::*Dv*NifD::HA		*D. vulgaris* NifD with C-terminal HA-tag targeted to mitochondria using MTP FAγ51	SN224
FAγ51::*Ct*NifD::HA		*C. tepidum* NifD with C-terminal HA-tag targeted to mitochondria using MTP FAγ51	SN225
FAγ51::*Ab*NifD::HA		*A. brasilense* NifD with C-terminal HA-tag targeted to mitochondria using MTP FAγ51	SN226
FAγ51::HA::S*f*NifK		*S. fredii* NifK with N-terminal HA-tag targeted to mitochondria using MTP FAγ51	SN356
FAγ51::HA::*Dv*NifK		*D. vulgaris* NifK with N-terminal HA-tag targeted to mitochondria using MTP FAγ51	SN357
FAγ51::HA::*Df*NifK		*D. ferrireducens* NifK with N-terminal HA-tag targeted to mitochondria using MTP FAγ51	SN358
FAγ51::HA::*Av*NifK		*A. vinelandii* NifK with N-terminal HA-tag targeted to mitochondria using MTP FAγ51	SN384
FAγ51::HA::A*b*NifK		*A. brasilense* NifK with N-terminal HA-tag targeted to mitochondria using MTP FAγ51	SN385
FAγ51::HA::*Ct*NifK		*C. tepidum* NifK with N-terminal HA-tag targeted to mitochondria using MTP FAγ51	SN388
Kanamycin resistance cassette			SN45
FAγ77::NifD::HA		NifD with C-terminal HA-tag targeted to mitochondria using MTP FAγ77 as described in Allen et al., 2017.	pRA19
FAγ77::NifD::HA - 5ATG		NifD with C-terminal HA-tag targeted to mitochondria using MTP FAγ77 with second to fifth start codons modified, based on pRA19.	pRA30
FAγ77::NifK		NifK targeted to mitochondria using MTP FAγ77	pRA25
Twin-strep::mTurquoise::metaxin	Metaxin	Twin-strep::mTurquoise::metaxin fusion protein for mitochondrial isolations	SN197
ala-FAγ51::*Df*NifD::HA		*D. ferrireducens* NifD, with an alanine scanned MTP to prevent mitochondrial processing	SN235
ala-FAγ51::*Sf*NifD::HA		*S. fredii* NifD, with an alanine scanned MTP to prevent mitochondrial processing	SN236
ala-FAγ51::*Av*NifD::HA		*A. vinelandii* NifD, with an alanine scanned MTP to prevent mitochondrial processing	SN237
ala-FAγ51::*Dv*NifD::HA		*D. vulgaris* NifD, with an alanine scanned MTP to prevent mitochondrial processing	SN238
ala-FAγ51::*Ct*NifD::HA		*C. tepidum* NifD, with an alanine scanned MTP to prevent mitochondrial processing	SN239
ala-FAγ51::*Ab*NifD::HA		*A. brasilense,* with an alanine scanned MTP to prevent mitochondrial processing	SN240

6xHis::*Df*NifD::HA		*D. ferrireducens* NifD with an N-terminal 6xHis-tag, expected to be located in the cytosol	SN241
6xHis::*Sf*NifD::HA		*S. fredii* NifD with an N-terminal 6xHis-tag, expected to be located in the cytosol	SN242
6xHis::*Av*NifD::HA		*A. vinelandii* NifD with an N-terminal 6xHis-tag, expected to be located in the cytosol	SN243
6xHis::*Dv*NifD::HA		*D. vulgaris* NifD with an N-terminal 6xHis-tag, expected to be located in the cytosol	SN244
6xHis::*Ct*NifD::HA		*C. tepidum* NifD with an N-terminal 6xHis-tag, expected to be located in the cytosol	SN245
6xHis::*Ab*NifD::HA		*A. brasilense* NifD with an N-terminal 6xHis-tag, expected to be located in the cytosol	SN246
MTP::NifD+ MTP::NifK combined		L2 vector combining MTP:NifD (SN10) and MTP:NifK(SN369)	SL94

* A short name was only given to constructs frequently used in this study.

** If not otherwise mentioned, the promoter is 2x35S. The terminator for all constructs is 35S.

SN constructs are single gene constructs, while SL constructs are multigene constructs. pRA are constructs based on vectors used in Allen et al., 2017.

Table S2: Protein sequences for Nif constructs used in this study

>SN4
MMYRFASNLASKARIAQNARQVSSRMSWSRNYMMTNATGERNLALIQEVLEVFPETARKERR
KHMMVSDPEMESVGKCIISNRKSQPGVMTVRGCAYAGSKGVVFGPIKDMAHISHGPVGCGQY
SRAERRNYYTGVSGVDSFGTLNFTSDFQERDIVFGGDKKLSKLIEEMELLFPLTKGITIQSE
CPVGLIGDDISAVANASSKALDKPVIPVRCEGFRGVSQSLGHHIANDVVRDWILNNREGQPF
ETTPYDVAIIGDYNIGGDAWASRILLEEMGLRVVAQWSGDGTLVEMENTPFVKLNLVHCYRS
MNYIARHMEEKHQIPWMEYNFFGPTKIAESLRKIADQFDDTIRANAEAVIARYEGQMAAIIA
KYRPRLEGRKVLLYMGGLRPRHVIGAYEDLGMEIIAAGYEFAHNDDYDRTLPDLKEGTLLFD
DASSYELEAFVKALKPDLIGSGIKEKYIFQKMGVPFRQMHSWDYSGPYHGYDGFAIFARDMD
MTLNNPAWNELTAPWLKSGGYPYDVPDYA

>SN6,7,8,9,10, SNY10
MMAMAVFRREGRRLLPSIAARPIAAIRSPLSSDQEEGLLGVRSISTQVVRNRGGMMTNATGE
RNLALIQEVLEVFPETARKERRKHMMVSDPEMESVGKCIISNRKSQPGVMTVRGCAYAGSKG
VVFGPIKDMAHISHGPVGCGQYSRAGRRNYYTGVSGVDSFGTLNFTSDFQERDIVFGGDKKL
SKLIEEMELLFPLTKGITIQSECPVGLIGDDISAVANASSKALDKPVIPVRCEGFRGVSQSL
GHHIANDVVRDWILNNREGQPFETTPYDVAIIGDYNIGGDAWASRILLEEMGLRVVAQWSGD
GTLVEMENTPFVKLNLVHCYRSMNYIARHMEEKHQIPWMEYNFFGPTKIAESLRKIADQFDD
TIRANAEAVIARYEGQMAAIIAKYRPRLEGRKVLLYMGGLRPRHVIGAYEDLGMEIIAAGYE
FAHNDDYDRTLPDLKEGTLLFDDASSYELEAFVKALKPDLIGSGIKEKYIFQKMGVPFRQMH
SWDYSGPYHGYDGFAIFARDMDMTLNNPAWNELTAPWLKSGGYPYDVPDYA

>SN11
MYRFASNLASKARIAQNARQVSSRMSWSRNYGGMMTNATGERNLALIQEVLEVFPETARKER
RKHMMVSDPEMESVGKCIISNRKSQPGVMTVRGCAYAGSKGVVFGPIKDMAHISHGPVGCGQ
YSRAGRRNYYTGVSGVDSFGTLNFTSDFQERDIVFGGDKKLSKLIEEMELLFPLTKGITIQS
ECPVGLIGDDISAVANASSKALDKPVIPVRCEGFRGVSQSLGHHIANDVVRDWILNNREGQP
FETTPYDVAIIGDYNIGGDAWASRILLEEMGLRVVAQWSGDGTLVEMENTPFVKLNLVHCYR
SMNYIARHMEEKHQIPWMEYNFFGPTKIAESLRKIADQFDDTIRANAEAVIARYEGQMAAII
AKYRPRLEGRKVLLYMGGLRPRHVIGAYEDLGMEIIAAGYEFAHNDDYDRTLPDLKEGTLLF
DDASSYELEAFVKALKPDLIGSGIKEKYIFQKMGVPFRQMHSWDYSGPYHGYDGFAIFARDM
DMTLNNPAWNELTAPWLKSGGYPYDVPDYA

>SN13
MAIRCVASRKTLAGLKETSSRLLRIRGIQGGMMTNATGERNLALIQEVLEVFPETARKERRK
HMMVSDPEMESVGKCIISNRKSQPGVMTVRGCAYAGSKGVVFGPIKDMAHISHGPVGCGQYS
RAGRRNYYTGVSGVDSFGTLNFTSDFQERDIVFGGDKKLSKLIEEMELLFPLTKGITIQSEC
PVGLIGDDISAVANASSKALDKPVIPVRCEGFRGVSQSLGHHIANDVVRDWILNNREGQPFE
TTPYDVAIIGDYNIGGDAWASRILLEEMGLRVVAQWSGDGTLVEMENTPFVKLNLVHCYRSM
NYIARHMEEKHQIPWMEYNFFGPTKIAESLRKIADQFDDTIRANAEAVIARYEGQMAAIIAK
YRPRLEGRKVLLYMGGLRPRHVIGAYEDLGMEIIAAGYEFAHNDDYDRTLPDLKEGTLLFDD
ASSYELEAFVKALKPDLIGSGIKEKYIFQKMGVPFRQMHSWDYSGPYHGYDGFAIFARDMDM
TLNNPAWNELTAPWLKSGGYPYDVPDYA

>SN14
MASTRVLASRLASQMAASAKVARPAVRVAQVSKRTIQTGSPLQTLKRTQMTSIVNATTRQAF
QKRAYSGGMMTNATGERNLALIQEVLEVFPETARKERRKHMMVSDPEMESVGKCIISNRKSQ
PGVMTVRGCAYAGSKGVVFGPIKDMAHISHGPVGCGQYSRAGRRNYYTGVSGVDSFGTLNFT
SDFQERDIVFGGDKKLSKLIEEMELLFPLTKGITIQSECPVGLIGDDISAVANASSKALDKP

VIPVRCEGFRGVSQSLGHHIANDVVRDWILNNREGQPFETTPYDVAIIGDYNIGGDAWASRI
LLEEMGLRVVAQWSGDGTLVEMENTPFVKLNLVHCYRSMNYIARHMEEKHQIPWMEYNFFGP
TKIAESLRKIADQFDDTIRANAEAVIARYEGQMAAIIAKYRPRLEGRKVLLYMGGLRPRHVI
GAYEDLGMEIIAAGYEFAHNDDYDRTLPDLKEGTLLFDDASSYELEAFVKALKPDLIGSGIK
EKYIFQKMGVPFRQMHSWDYSGPYHGYDGFAIFARDMDMTLNNPAWNELTAPWLKSGGYPYD
VPDYA

>SN33
MYPYDVPDYAGGMMTNATGERNLALIQEVLEVFPETARKERRKHMMVSDPEMESVGKCIISN
RKSQPGVMTVRGCAYAGSKGVVFGPIKDMAHISHGPVGCGQYSRAGRRNYYTGVSGVDSFGT
LNFTSDFQERDIVFGGDKKLSKLIEEMELLFPLTKGITIQSECPVGLIGDDISAVANASSKA
LDKPVIPVRCEGFRGVSQSLGHHIANDVVRDWILNNREGQPFETTPYDVAIIGDYNIGGDAW
ASRILLEEMGLRVVAQWSGDGTLVEMENTPFVKLNLVHCYRSMNYIARHMEEKHQIPWMEYN
FFGPTKIAESLRKIADQFDDTIRANAEAVIARYEGQMAAIIAKYRPRLEGRKVLLYMGGLRP
RHVIGAYEDLGMEIIAAGYEFAHNDDYDRTLPDLKEGTLLFDDASSYELEAFVKALKPDLIG
SGIKEKYIFQKMGVPFRQMHSWDYSGPYHGYDGFAIFARDMDMTLNNPAWNELTAPWLKS

>SN34
MYPYDVPDYAGGMMTNATGERNLALIQEVLEVFPETARKERRKHMMVSDPEMESVGKCIISN
RKSQPGVMTVRGCAYAGSKGVVFGPIKDMAHISHGPVGCGQYSRAGRRNYYTGVSGVDSFGT
LNFTSDFQERDIVFGGDKKLSKLIEEMELLFPLTKGITIQSECPVGLIGDDISAVANASSKA
LDKPVIPVRCEGFRGVSQSLGHHIANDVVRDWILNNREGQPFETTPYDVAIIGDYNIGGDAW
ASRILLEEMGLRVVAQWSGDGTLVEMENTPFVKLNLVHCYRSMNYIARHMEEKHQIPWMEYN
FFGPTKIAESLRKIADQFDDTIRANAEAVIARYEGQMAAIIAKYRPRLEGRKVLLYMGGLRP
RHVIGAYEDLGMEIIAAGYEFAHNDDYDRTLPDLKEGTLLFDDASSYELEAFVKALKPDLIG
SGIKEKYIFQKMGVPFRQMHSWDYSGPYHGYDGFAIFARDMDMTLNNPAWNELTAPWLKSGG
YPYDVPDYA

>SN45
MIEQDGLHAGSPAAWVERLFGYDWAQQTIGCSDAAVFRLSAQGRPVLFVKTDLSGALNELQD
EAARLSWLATTGVPCAAVLDVVTEAGRDWLLLGEVPGQDLLSSHLAPAEKVSIMADAMRRLH
TLDPATCPFDHQAKHRIERGRTRMEAGLVDQDDLDEEHQGLAPAELFARLKARMPDGEDLVV
TQGDACLPNIMVENGRFSGFIDCGRLGVADRYQDIALATRDIAEELGGEWADRFLVLYGIAA
PDSQRIAFYRLLDEFF

>SN46/SN369
MSQTIDKINSCYPLFEQDEYQELFRNKRQLEEAHDAQRVQEVFAWTTTAEYEALNFQREALT
VDPAKACQPLGAVLCSLGFANTLPYVHGSQGCVAYFRTYFNRHFKEPIACVSDSMTEDAAVF
GGNNNMNLGLQNASALYKPEIIAVSTTCMAEVIGDDLQAFIANAKKDGFVDSSIAVPHAHTP
SFIGSHVTGWDNMFEGFAKTFTADYQGQPGKLPKLNLVTGFETYLGNFRVLKRMMEQMAVPC
SLLSDPSEVLDTPADGHYRMYSGGTTQQEMKEAPDAIDTLLLQPWQLLKSKKVVQEMWNQPA
TEVAIPLGLAATDELLMTVSQLSGKPIADALTLERGRLVDMMLDSHTWLHGKKFGLYGDPDF
VMGLTRFLLELGCEPTVILSHNANKRWQKAMNKMLDASPYGRDSEVFINCDLWHFRSLMFTR
QPDFMIGNSYGKFIQRDTLAKGKAFEVPLIRLGFPLFDRHHLHRQTTWGYEGAMNIVTTLVN
AVLEKLDSDTSQLGKTDYSFDLVR

>SN52
MMAMAVFRREGRRLLPSIAARPIAAIRSPLSSDQEEGLLGVRSISTQVVRNRGGMMTNATGE
RNLALIQEVLEVFPETARKERRKHMMVSDPEMESVGKCIIAAAAAQPGVMTVRGCAYAGSKG
VVFGPIKDMAHISHGPVGCGQYSRAGRRNQYTGVSGVDSFGTLNFTSDFQERDIVFGGDKKL
SKLIEEMELLFPLTKGITIQSECPVGLIGDDISAVANASSKALDKPVIPVRCEGFRGVSQSL

GHHIANDVVRDWILNNREGQPFETTPYDVAIIGDYNIGGDAWASRILLEEMGLRVVAQWSGD
GTLVEMENTPFVKLNLVHCYRSMNYIARHMEEKHQIPWMEYNFFGPTKIAESLRKIADQFDD
TIRANAEAVIARYEGQMAAIIAKYRPRLEGRKVLLYMGGLRPRHVIGAYEDLGMEIIAAGYE
FAHNDDYDRTLPDLKEGTLLFDDASSYELEAFVKALKPDLIGSGIKEKYIFQKMGVPFRQMH
SWDYSGPYHGYDGFAIFARDMDMTLNNPAWNELTAPWLKSGGYPYDVPDYA

>SN53
MMAMAVFRREGRRLLPSIAARPIAAIRSPLSSDQEEGLLGVRSISTQVVRNRGGMMTNATGE
RNLALIQEVLEVFPETARKERRKHMMVSDPEMESVGKCIISNRKSAAAAATVRGCAYAGSKG
VVFGPIKDMAHISHGPVGCGQYSRAGRRNYYTGVSGVDSFGTLNFTSDFQERDIVFGGDKKL
SKLIEEMELLFPLTKGITIQSECPVGLIGDDISAVANASSKALDKPVIPVRCEGFRGVSQSL
GHHIANDVVRDWILNNREGQPFETTPYDVAIIGDYNIGGDAWASRILLEEMGLRVVAQWSGD
GTLVEMENTPFVKLNLVHCYRSMNYIARHMEEKHQIPWMEYNFFGPTKIAESLRKIADQFDD
TIRANAEAVIARYEGQMAAIIAKYRPRLEGRKVLLYMGGLRPRHVIGAYEDLGMEIIAAGYE
FAHNDDYDRTLPDLKEGTLLFDDASSYELEAFVKALKPDLIGSGIKEKYIFQKMGVPFRQMH
SWDYSGPYHGYDGFAIFARDMDMTLNNPAWNELTAPWLKSGGYPYDVPDYA

>SN54
MMAMAVFRREGRRLLPSIAARPIAAIRSPLSSDQEEGLLGVRSISTQVVRNRGGMMTNATGE
RNLALIQEVLEVFPETARKERRKHMMVSDPEMESVGKCIISNRKSQPGVMAAAAAAYAGSKG
VVFGPIKDMAHISHGPVGCGQYSRAGRRNYYTGVSGVDSFGTLNFTSDFQERDIVFGGDKKL
SKLIEEMELLFPLTKGITIQSECPVGLIGDDISAVANASSKALDKPVIPVRCEGFRGVSQSL
GHHIANDVVRDWILNNREGQPFETTPYDVAIIGDYNIGGDAWASRILLEEMGLRVVAQWSGD
GTLVEMENTPFVKLNLVHCYRSMNYIARHMEEKHQIPWMEYNFFGPTKIAESLRKIADQFDD
TIRANAEAVIARYEGQMAAIIAKYRPRLEGRKVLLYMGGLRPRHVIGAYEDLGMEIIAAGYE
FAHNDDYDRTLPDLKEGTLLFDDASSYELEAFVKALKPDLIGSGIKEKYIFQKMGVPFRQMH
SWDYSGPYHGYDGFAIFARDMDMTLNNPAWNELTAPWLKSGGYPYDVPDYA

>SN55
MMAMAVFRREGRRLLPSIAARPIAAIRSPLSSDQEEGLLGVRSISTQVVRNRGGMMTNATGE
RNLALIQEVLEVFPETARKERRKHMMVSDPEMESVGKCIISNRKSQPGVMTVRGCGAGAAKG
VVFGPIKDMAHISHGPVGCGQYSRAGRRNYYTGVSGVDSFGTLNFTSDFQERDIVFGGDKKL
SKLIEEMELLFPLTKGITIQSECPVGLIGDDISAVANASSKALDKPVIPVRCEGFRGVSQSL
GHHIANDVVRDWILNNREGQPFETTPYDVAIIGDYNIGGDAWASRILLEEMGLRVVAQWSGD
GTLVEMENTPFVKLNLVHCYRSMNYIARHMEEKHQIPWMEYNFFGPTKIAESLRKIADQFDD
TIRANAEAVIARYEGQMAAIIAKYRPRLEGRKVLLYMGGLRPRHVIGAYEDLGMEIIAAGYE
FAHNDDYDRTLPDLKEGTLLFDDASSYELEAFVKALKPDLIGSGIKEKYIFQKMGVPFRQMH
SWDYSGPYHGYDGFAIFARDMDMTLNNPAWNELTAPWLKSGGYPYDVPDYA

>SN56
MMAMAVFRREGRRLLPSIAARPIAAIRSPLSSDQEEGLLGVRSISTQVVRNRGGMMTNATGE
RNLALIQEVLEVFPETARKERRKHMMVSDPEMESVGKCIISNRKSQPGVMTVRGCAYAGSAA
AAAGPIKDMAHISHGPVGCGQYSRAGRRNYYTGVSGVDSFGTLNFTSDFQERDIVFGGDKKL
SKLIEEMELLFPLTKGITIQSECPVGLIGDDISAVANASSKALDKPVIPVRCEGFRGVSQSL
GHHIANDVVRDWILNNREGQPFETTPYDVAIIGDYNIGGDAWASRILLEEMGLRVVAQWSGD
GTLVEMENTPFVKLNLVHCYRSMNYIARHMEEKHQIPWMEYNFFGPTKIAESLRKIADQFDD
TIRANAEAVIARYEGQMAAIIAKYRPRLEGRKVLLYMGGLRPRHVIGAYEDLGMEIIAAGYE
FAHNDDYDRTLPDLKEGTLLFDDASSYELEAFVKALKPDLIGSGIKEKYIFQKMGVPFRQMH
SWDYSGPYHGYDGFAIFARDMDMTLNNPAWNELTAPWLKSGGYPYDVPDYA

>SN57
MMAMAVFRREGRRLLPSIAARPIAAIRSPLSSDQEEGLLGVRSISTQVVRNRGGMMTNATGE
RNLALIQEVLEVFPETARKERRKHMMVSDPEMESVGKCIISNRKSQPGVMTVRGCAYAGSKG
VVFGPIKDAAAAAHGPVGCGQYSRAGRRNYYTGVSGVDSFGTLNFTSDFQERDIVFGGDKKL
SKLIEEMELLFPLTKGITIQSECPVGLIGDDISAVANASSKALDKPVIPVRCEGFRGVSQSL
GHHIANDVVRDWILNNREGQPFETTPYDVAIIGDYNIGGDAWASRILLEEMGLRVVAQWSGD
GTLVEMENTPFVKLNLVHCYRSMNYIARHMEEKHQIPWMEYNFFGPTKIAESLRKIADQFDD
TIRANAEAVIARYEGQMAAIIAKYRPRLEGRKVLLYMGGLRPRHVIGAYEDLGMEIIAAGYE
FAHNDDYDRTLPDLKEGTLLFDDASSYELEAFVKALKPDLIGSGIKEKYIFQKMGVPFRQMH
SWDYSGPYHGYDGFAIFARDMDMTLNNPAWNELTAPWLKSGGYPYDVPDYA

>SN75
MAMAVFRREGRRLLPSIAARPIAAIRSPLSSDQEEGLLGVRSISTQVVRNRGGYPYDVPDYA
GGMMTNATGERNLALIQEVLEVFPETARKERRKHMMVSDPEMESVGKCIISNRKSQPGVMTV
RGCAYAGSKGVVFGPIKDMAHISHGPVGCGQYSRAGRRNYYTGVSGVDSFGTLNFTSDFQER
DIVFGGDKKLSKLIEEMELLFPLTKGITIQSECPVGLIGDDISAVANASSKALDKPVIPVRC
EGFRGVSQSLGHHIANDVVRDWILNNREGQPFETTPYDVAIIGDYNIGGDAWASRILLEEMG
LRVVAQWSGDGTLVEMENTPFVKLNLVHCYRSMNYIARHMEEKHQIPWMEYNFFGPTKIAES
LRKIADQFDDTIRANAEAVIARYEGQMAAIIAKYRPRLEGRKVLLYMGGLRPRHVIGAYEDL
GMEIIAAGYEFAHNDDYDRTLPDLKEGTLLFDDASSYELEAFVKALKPDLIGSGIKEKYIFQ
KMGVPFRQMHSWDYSGPYHGYDGFAIFARDMDMTLNNPAWNELTAPWLKSGGYPYDVPDYA

>SN68
MMAMAVFRREGRRLLPSIAARPIAAIRSPLSSDQEEGLLGVRSISTQVVRNRGGMTNATGER
NLALIQEVLEVFPETARKERRKHMMVSDPEMESVGKCIISNRKSQPGVMTVRGCAYAGSKGV
VFGPIKDMAHISHGPVGCGQYSRAGRRNYYTGVSGVDSFGTLNFTSDFQERDIVFGGDKKLS
KLIEEMELLFPLTKGITIQSECPVGLIGDDISAVANASSKALDKPVIPVRCEGFRGVSQSLG
HHIANDVVRDWILNNREGQPFETTPYDVAIIGDYNIGGDAWASRILLEEMGLRVVAQWSGDG
TLVEMENTPFVKLNLVHCYRSMNYIARHMEEKHQIPWMEYNFFGPTKIAESLRKIADQFDDT
IRANAEAVIARYEGQMAAIIAKYRPRLEGRKVLLYMGGLRPRHVIGAYEDLGMEIIAAGYEF
AHNDDYDRTLPDLKEGTLLFDDASSYELEAFVKALKPDLIGSGIKEKYIFQKMGVPFRQMHS
WDYSGPYHGYDGFAIFARDMDMTLNNPAWNELTAPWLKSAATPPPGSTTTAYPYDVPDYATP
PPGSTTTASQTIDKINSCYPLFEQDEYQELFRNKRQLEEAHDAQRVQEVFAWTTTAEYEALN
FQREALTVDPAKACQPLGAVLCSLGFANTLPYVHGSQGCVAYFRTYFNRHFKEPIACVSDSM
TEDAAVFGGNNNMNLGLQNASALYKPEIIAVSTTCMAEVIGDDLQAFIANAKKDGFVDSSIA
VPHAHTPSFIGSHVTGWDNMFEGFAKTFTADYQGQPGKLPKLNLVTGFETYLGNFRVLKRMM
EQMAVPCSLLSDPSEVLDTPADGHYRMYSGGTTQQEMKEAPDAIDTLLLQPWQLLKSKKVVQ
EMWNQPATEVAIPLGLAATDELLMTVSQLSGKPIADALTLERGRLVDMMLDSHTWLHGKKFG
LYGDPDFVMGLTRFLLELGCEPTVILSHNANKRWQKAMNKMLDASPYGRDSEVFINCDLWHF
RSLMFTRQPDFMIGNSYGKFIQRDTLAKGKAFEVPLIRLGFPLFDRHHLHRQTTWGYEGAMN
IVTTLVNAVLEKLDSDTSQLGKTDYSFDLVR

>SN96
MMAMAVFRREGRRLLPSIAARPIAAIRSPLSSDQEEGLLGVRSISTQVVRNRGGMMTNATGE
RNLALIQEVLEVFPETARKERRKHMMVSDPEMESVGKCIISNRKSQPGVMTVRGCAYAGSKG
VVFAGAAAMAHISHGPVGCGQYSRAGRRNYYTGVSGVDSFGTLNFTSDFQERDIVFGGDKKL
SKLIEEMELLFPLTKGITIQSECPVGLIGDDISAVANASSKALDKPVIPVRCEGFRGVSQSL
GHHIANDVVRDWILNNREGQPFETTPYDVAIIGDYNIGGDAWASRILLEEMGLRVVAQWSGD
GTLVEMENTPFVKLNLVHCYRSMNYIARHMEEKHQIPWMEYNFFGPTKIAESLRKIADQFDD
TIRANAEAVIARYEGQMAAIIAKYRPRLEGRKVLLYMGGLRPRHVIGAYEDLGMEIIAAGYE

FAHNDDYDRTLPDLKEGTLLFDDASSYELEAFVKALKPDLIGSGIKEKYIFQKMGVPFRQMH
SWDYSGPYHGYDGFAIFARDMDMTLNNPAWNELTAPWLKSGGYPYDVPDYA

>SN97
MMAMAVFRREGRRLLPSIAARPIAAIRSPLSSDQEEGLLGVRSISTQVVRNRGGMMTNATGE
RNLALIQEVLEVFPETARKERRKHMMVSDPEMESVGKCIISNRKSQPGVMTVRGCAYAGSKG
VVFGPIKDMAHISAAAAACGQYSRAGRRNYYTGVSGVDSFGTLNFTSDFQERDIVFGGDKKL
SKLIEEMELLFPLTKGITIQSECPVGLIGDDISAVANASSKALDKPVIPVRCEGFRGVSQSL
GHHIANDVVRDWILNNREGQPFETTPYDVAIIGDYNIGGDAWASRILLEEMGLRVVAQWSGD
GTLVEMENTPFVKLNLVHCYRSMNYIARHMEEKHQIPWMEYNFFGPTKIAESLRKIADQFDD
TIRANAEAVIARYEGQMAAIIAKYRPRLEGRKVLLYMGGLRPRHVIGAYEDLGMEIIAAGYE
FAHNDDYDRTLPDLKEGTLLFDDASSYELEAFVKALKPDLIGSGIKEKYIFQKMGVPFRQMH
SWDYSGPYHGYDGFAIFARDMDMTLNNPAWNELTAPWLKSGGYPYDVPDYA

>SN98
MMAMAVFRREGRRLLPSIAARPIAAIRSPLSSDQEEGLLGVRSISTQVVRNRGGMMTNATGE
RNLALIQEVLEVFPETARKERRKHMMVSDPEMESVGKCIISNRKSQPGVMTVRGCAYAGSKG
VVFGPIKDMAHISHGPVGCGQYSAGAAANYYTGVSGVDSFGTLNFTSDFQERDIVFGGDKKL
SKLIEEMELLFPLTKGITIQSECPVGLIGDDISAVANASSKALDKPVIPVRCEGFRGVSQSL
GHHIANDVVRDWILNNREGQPFETTPYDVAIIGDYNIGGDAWASRILLEEMGLRVVAQWSGD
GTLVEMENTPFVKLNLVHCYRSMNYIARHMEEKHQIPWMEYNFFGPTKIAESLRKIADQFDD
TIRANAEAVIARYEGQMAAIIAKYRPRLEGRKVLLYMGGLRPRHVIGAYEDLGMEIIAAGYE
FAHNDDYDRTLPDLKEGTLLFDDASSYELEAFVKALKPDLIGSGIKEKYIFQKMGVPFRQMH
SWDYSGPYHGYDGFAIFARDMDMTLNNPAWNELTAPWLKSGGYPYDVPDYA

>SN99
MMAMAVFRREGRRLLPSIAARPIAAIRSPLSSDQEEGLLGVRSISTQVVRNRGGMMTNATGE
RNLALIQEVLEVFPETARKERRKHMMVSDPEMESVGKCIISNRKSQPGVMTVRGCAYAGSKG
VVFGPIKDMAHISHGPVGCGQYSAGAAANYYTGVSGVDSFGTLNFTSDFQERDIVFGGDKKL
SKLIEEMELLFPLTKGITIQSECPVGLIGDDISAVANASSKALDKPVIPVRCEGFRGVSQSL
GHHIANDVVRDWILNNREGQPFETTPYDVAIIGDYNIGGDAWASRILLEEMGLRVVAQWSGD
GTLVEMENTPFVKLNLVHCYRSMNYIARHMEEKHQIPWMEYNFFGPTKIAESLRKIADQFDD
TIRANAEAVIARYEGQMAAIIAKYRPRLEGRKVLLYMGGLRPRHVIGAYEDLGMEIIAAGYE
FAHNDDYDRTLPDLKEGTLLFDDASSYELEAFVKALKPDLIGSGIKEKYIFQKMGVPFRQMH
SWDYSGPYHGYDGFAIFARDMDMTLNNPAWNELTAPWLKSGGYPYDVPDYA

>SN100
MMAMAVFRREGRRLLPSIAARPIAAIRSPLSSDQEEGLLGVRSISTQVVRNRGGMMTNATGE
RNLALIQEVLEVFPETARKERRKHMMVSDPEMESVGKCIISNRKSQPGVMTVRGCAYAGSKG
VVFGPIKDMAHISHGPVGCGQYSRAGRRAAAAAVSGVDSFGTLNFTSDFQERDIVFGGDKKL
SKLIEEMELLFPLTKGITIQSECPVGLIGDDISAVANASSKALDKPVIPVRCEGFRGVSQSL
GHHIANDVVRDWILNNREGQPFETTPYDVAIIGDYNIGGDAWASRILLEEMGLRVVAQWSGD
GTLVEMENTPFVKLNLVHCYRSMNYIARHMEEKHQIPWMEYNFFGPTKIAESLRKIADQFDD
TIRANAEAVIARYEGQMAAIIAKYRPRLEGRKVLLYMGGLRPRHVIGAYEDLGMEIIAAGYE
FAHNDDYDRTLPDLKEGTLLFDDASSYELEAFVKALKPDLIGSGIKEKYIFQKMGVPFRQMH
SWDYSGPYHGYDGFAIFARDMDMTLNNPAWNELTAPWLKSGGYPYDVPDYA

>SN101
MMAMAVFRREGRRLLPSIAARPIAAIRSPLSSDQEEGLLGVRSISTQVVRNRGGMMTNATGE
RNLALIQEVLEVFPETARKERRKHMMVSDPEMESVGKCIISNRKSQPGVMTVRGCAYAGSKG

VVFGPIKDMAHISHGPVGCGQYSRAGRRNYYTGAAAAASFGTLNFTSDFQERDIVFGGDKKL
SKLIEEMELLFPLTKGITIQSECPVGLIGDDISAVANASSKALDKPVIPVRCEGFRGVSQSL
GHHIANDVVRDWILNNREGQPFETTPYDVAIIGDYNIGGDAWASRILLEEMGLRVVAQWSGD
GTLVEMENTPFVKLNLVHCYRSMNYIARHMEEKHQIPWMEYNFFGPTKIAESLRKIADQFDD
TIRANAEAVIARYEGQMAAIIAKYRPRLEGRKVLLYMGGLRPRHVIGAYEDLGMEIIAAGYE
FAHNDDYDRTLPDLKEGTLLFDDASSYELEAFVKALKPDLIGSGIKEKYIFQKMGVPFRQMH
SWDYSGPYHGYDGFAIFARDMDMTLNNPAWNELTAPWLKSGGYPYDVPDYA

>SN108
MMAMAVFRREGRRLLPSIAARPIAAIRSPLSSDQEEGLLGVRSISTQVVRNRGGMMTNATGE
RNLALIQEVLEVFPETARKERRKHMMVSDPEMESVGKCIISNRKSQPGVMTVRGCAYAGSKG
VVFGPIKDMAHISHGPVGCGQYSRAGRRNYFTGVSGVDSFGTLNFTSDFQERDIVFGGDKKL
SKLIEEMELLFPLTKGITIQSECPVGLIGDDISAVANASSKALDKPVIPVRCEGFRGVSQSL
GHHIANDVVRDWILNNREGQPFETTPYDVAIIGDYNIGGDAWASRILLEEMGLRVVAQWSGD
GTLVEMENTPFVKLNLVHCYRSMNYIARHMEEKHQIPWMEYNFFGPTKIAESLRKIADQFDD
TIRANAEAVIARYEGQMAAIIAKYRPRLEGRKVLLYMGGLRPRHVIGAYEDLGMEIIAAGYE
FAHNDDYDRTLPDLKEGTLLFDDASSYELEAFVKALKPDLIGSGIKEKYIFQKMGVPFRQMH
SWDYSGPYHGYDGFAIFARDMDMTLNNPAWNELTAPWLKSGGYPYDVPDYA

>SN109
MMAMAVFRREGRRLLPSIAARPIAAIRSPLSSDQEEGLLGVRSISTQVVRNRGGMMTNATGE
RNLALIQEVLEVFPETARKERRKHMMVSDPEMESVGKCIISNRKSQPGVMTVRGCAYAGSKG
VVFGPIKDMAHISHGPVGCGQYSRAGRRNYATGVSGVDSFGTLNFTSDFQERDIVFGGDKKL
SKLIEEMELLFPLTKGITIQSECPVGLIGDDISAVANASSKALDKPVIPVRCEGFRGVSQSL
GHHIANDVVRDWILNNREGQPFETTPYDVAIIGDYNIGGDAWASRILLEEMGLRVVAQWSGD
GTLVEMENTPFVKLNLVHCYRSMNYIARHMEEKHQIPWMEYNFFGPTKIAESLRKIADQFDD
TIRANAEAVIARYEGQMAAIIAKYRPRLEGRKVLLYMGGLRPRHVIGAYEDLGMEIIAAGYE
FAHNDDYDRTLPDLKEGTLLFDDASSYELEAFVKALKPDLIGSGIKEKYIFQKMGVPFRQMH
SWDYSGPYHGYDGFAIFARDMDMTLNNPAWNELTAPWLKSGGYPYDVPDYA

>SN110
MMAMAVFRREGRRLLPSIAARPIAAIRSPLSSDQEEGLLGVRSISTQVVRNRGGMMTNATGE
RNLALIQEVLEVFPETARKERRKHMMVSDPEMESVGKCIISNRKSQPGVMTVRGCAYAGSKG
VVFGPIKDMAHISHGPVGCGQYSRAGRRNYFAGVSGVDSFGTLNFTSDFQERDIVFGGDKKL
SKLIEEMELLFPLTKGITIQSECPVGLIGDDISAVANASSKALDKPVIPVRCEGFRGVSQSL
GHHIANDVVRDWILNNREGQPFETTPYDVAIIGDYNIGGDAWASRILLEEMGLRVVAQWSGD
GTLVEMENTPFVKLNLVHCYRSMNYIARHMEEKHQIPWMEYNFFGPTKIAESLRKIADQFDD
TIRANAEAVIARYEGQMAAIIAKYRPRLEGRKVLLYMGGLRPRHVIGAYEDLGMEIIAAGYE
FAHNDDYDRTLPDLKEGTLLFDDASSYELEAFVKALKPDLIGSGIKEKYIFQKMGVPFRQMH
SWDYSGPYHGYDGFAIFARDMDMTLNNPAWNELTAPWLKSGGYPYDVPDYA

>SN111
MMAMAVFRREGRRLLPSIAARPIAAIRSPLSSDQEEGLLGVRSISTQVVRNRGGMMTNATGE
RNLALIQEVLEVFPETARKERRKHMMVSDPEMESVGKCIISNRKSQPGVMTVRGCAYAGSKG
VVFGPIKDMAHISHGPVGCGQYSRAGRRNYYAAVSGVDSFGTLNFTSDFQERDIVFGGDKKL
SKLIEEMELLFPLTKGITIQSECPVGLIGDDISAVANASSKALDKPVIPVRCEGFRGVSQSL
GHHIANDVVRDWILNNREGQPFETTPYDVAIIGDYNIGGDAWASRILLEEMGLRVVAQWSGD
GTLVEMENTPFVKLNLVHCYRSMNYIARHMEEKHQIPWMEYNFFGPTKIAESLRKIADQFDD
TIRANAEAVIARYEGQMAAIIAKYRPRLEGRKVLLYMGGLRPRHVIGAYEDLGMEIIAAGYE
FAHNDDYDRTLPDLKEGTLLFDDASSYELEAFVKALKPDLIGSGIKEKYIFQKMGVPFRQMH
SWDYSGPYHGYDGFAIFARDMDMTLNNPAWNELTAPWLKSGGYPYDVPDYA

>SN112
MMAMAVFRREGRRLLPSIAARPIAAIRSPLSSDQEEGLLGVRSISTQVVRNRGGMMTNATGE
RNLALIQEVLEVFPETARKERRKHMMVSDPEMESVGKCIISNRKSQPGVMTVRGCAYAGSKG
VVFGPIKDMAHISHGPVGCGQYSRAGRANYYTGVSGVDSFGTLNFTSDFQERDIVFGGDKKL
SKLIEEMELLFPLTKGITIQSECPVGLIGDDISAVANASSKALDKPVIPVRCEGFRGVSQSL
GHHIANDVVRDWILNNREGQPFETTPYDVAIIGDYNIGGDAWASRILLEEMGLRVVAQWSGD
GTLVEMENTPFVKLNLVHCYRSMNYIARHMEEKHQIPWMEYNFFGPTKIAESLRKIADQFDD
TIRANAEAVIARYEGQMAAIIAKYRPRLEGRKVLLYMGGLRPRHVIGAYEDLGMEIIAAGYE
FAHNDDYDRTLPDLKEGTLLFDDASSYELEAFVKALKPDLIGSGIKEKYIFQKMGVPFRQMH
SWDYSGPYHGYDGFAIFARDMDMTLNNPAWNELTAPWLKSGGYPYDVPDYA

>SN113
MMAMAVFRREGRRLLPSIAARPIAAIRSPLSSDQEEGLLGVRSISTQVVRNRGGMMTNATGE
RNLALIQEVLEVFPETARKERRKHMMVSDPEMESVGKCIISNRKSQPGVMTVRGCAYAGSKG
VVFGPIKDMAHISHGPVGCGQYSRAGRRHYYTGVSGVDSFGTLNFTSDFQERDIVFGGDKKL
SKLIEEMELLFPLTKGITIQSECPVGLIGDDISAVANASSKALDKPVIPVRCEGFRGVSQSL
GHHIANDVVRDWILNNREGQPFETTPYDVAIIGDYNIGGDAWASRILLEEMGLRVVAQWSGD
GTLVEMENTPFVKLNLVHCYRSMNYIARHMEEKHQIPWMEYNFFGPTKIAESLRKIADQFDD
TIRANAEAVIARYEGQMAAIIAKYRPRLEGRKVLLYMGGLRPRHVIGAYEDLGMEIIAAGYE
FAHNDDYDRTLPDLKEGTLLFDDASSYELEAFVKALKPDLIGSGIKEKYIFQKMGVPFRQMH
SWDYSGPYHGYDGFAIFARDMDMTLNNPAWNELTAPWLKSGGYPYDVPDYA

>SN114, SNY114
MMAMAVFRREGRRLLPSIAARPIAAIRSPLSSDQEEGLLGVRSISTQVVRNRGGMMTNATGE
RNLALIQEVLEVFPETARKERRKHMMVSDPEMESVGKCIISNRKSQPGVMTVRGCAYAGSKG
VVFGPIKDMAHISHGPVGCGQYSRAGRRNQYTGVSGVDSFGTLNFTSDFQERDIVFGGDKKL
SKLIEEMELLFPLTKGITIQSECPVGLIGDDISAVANASSKALDKPVIPVRCEGFRGVSQSL
GHHIANDVVRDWILNNREGQPFETTPYDVAIIGDYNIGGDAWASRILLEEMGLRVVAQWSGD
GTLVEMENTPFVKLNLVHCYRSMNYIARHMEEKHQIPWMEYNFFGPTKIAESLRKIADQFDD
TIRANAEAVIARYEGQMAAIIAKYRPRLEGRKVLLYMGGLRPRHVIGAYEDLGMEIIAAGYE
FAHNDDYDRTLPDLKEGTLLFDDASSYELEAFVKALKPDLIGSGIKEKYIFQKMGVPFRQMH
SWDYSGPYHGYDGFAIFARDMDMTLNNPAWNELTAPWLKSGGYPYDVPDYA

>SN115
MMAMAVFRREGRRLLPSIAARPIAAIRSPLSSDQEEGLLGVRSISTQVVRNRGGMMTNATGE
RNLALIQEVLEVFPETARKERRKHMMVSDPEMESVGKCIISNRKSQPGVMTVRGCAYAGSKG
VVFGPIKDMAHISHGPVGCGQYSRAGRRNYTTGVSGVDSFGTLNFTSDFQERDIVFGGDKKL
SKLIEEMELLFPLTKGITIQSECPVGLIGDDISAVANASSKALDKPVIPVRCEGFRGVSQSL
GHHIANDVVRDWILNNREGQPFETTPYDVAIIGDYNIGGDAWASRILLEEMGLRVVAQWSGD
GTLVEMENTPFVKLNLVHCYRSMNYIARHMEEKHQIPWMEYNFFGPTKIAESLRKIADQFDD
TIRANAEAVIARYEGQMAAIIAKYRPRLEGRKVLLYMGGLRPRHVIGAYEDLGMEIIAAGYE
FAHNDDYDRTLPDLKEGTLLFDDASSYELEAFVKALKPDLIGSGIKEKYIFQKMGVPFRQMH
SWDYSGPYHGYDGFAIFARDMDMTLNNPAWNELTAPWLKSGGYPYDVPDYA

>SN116
MMAMAVFRREGRRLLPSIAARPIAAIRSPLSSDQEEGLLGVRSISTQVVRNRGGMMTNATGE
RNLALIQEVLEVFPETARKERRKHMMVSDPEMESVGKCIISNRKSQPGVMTVRGCAYAGSKG
VVFGPIKDMAHISHGPVGCGQYSRAGRRNYYVGVSGVDSFGTLNFTSDFQERDIVFGGDKKL
SKLIEEMELLFPLTKGITIQSECPVGLIGDDISAVANASSKALDKPVIPVRCEGFRGVSQSL
GHHIANDVVRDWILNNREGQPFETTPYDVAIIGDYNIGGDAWASRILLEEMGLRVVAQWSGD

GTLVEMENTPFVKLNLVHCYRSMNYIARHMEEKHQIPWMEYNFFGPTKIAESLRKIADQFDD
TIRANAEAVIARYEGQMAAIIAKYRPRLEGRKVLLYMGGLRPRHVIGAYEDLGMEIIAAGYE
FAHNDDYDRTLPDLKEGTLLFDDASSYELEAFVKALKPDLIGSGIKEKYIFQKMGVPFRQMH
SWDYSGPYHGYDGFAIFARDMDMTLNNPAWNELTAPWLKSGGYPYDVPDYA

>SN117
MMAMAVFRREGRRLLPSIAARPIAAIRSPLSSDQEEGLLGVRSISTQVVRNRGGMMTNATGE
RNLALIQEVLEVFPETARKERRKHMMVSDPEMESVGKCIISNRKSQPGVMTVRGCAYAGSKG
VVFGPIKDMAHISHGPVGCGQYSRAGRRNQTTGVSGVDSFGTLNFTSDFQERDIVFGGDKKL
SKLIEEMELLFPLTKGITIQSECPVGLIGDDISAVANASSKALDKPVIPVRCEGFRGVSQSL
GHHIANDVVRDWILNNREGQPFETTPYDVAIIGDYNIGGDAWASRILLEEMGLRVVAQWSGD
GTLVEMENTPFVKLNLVHCYRSMNYIARHMEEKHQIPWMEYNFFGPTKIAESLRKIADQFDD
TIRANAEAVIARYEGQMAAIIAKYRPRLEGRKVLLYMGGLRPRHVIGAYEDLGMEIIAAGYE
FAHNDDYDRTLPDLKEGTLLFDDASSYELEAFVKALKPDLIGSGIKEKYIFQKMGVPFRQMH
SWDYSGPYHGYDGFAIFARDMDMTLNNPAWNELTAPWLKSGGYPYDVPDYA

>SN118
MMAMAVFRREGRRLLPSIAARPIAAIRSPLSSDQEEGLLGVRSISTQVVRNRGGMMTNATGE
RNLALIQEVLEVFPETARKERRKHMMVSDPEMESVGKCIISNRKSQPGVMTVRGCAYAGSKG
VVFGPIKDMAHISHGPVGCGQYSRAGRRHKGTGVSGVDSFGTLNFTSDFQERDIVFGGDKKL
SKLIEEMELLFPLTKGITIQSECPVGLIGDDISAVANASSKALDKPVIPVRCEGFRGVSQSL
GHHIANDVVRDWILNNREGQPFETTPYDVAIIGDYNIGGDAWASRILLEEMGLRVVAQWSGD
GTLVEMENTPFVKLNLVHCYRSMNYIARHMEEKHQIPWMEYNFFGPTKIAESLRKIADQFDD
TIRANAEAVIARYEGQMAAIIAKYRPRLEGRKVLLYMGGLRPRHVIGAYEDLGMEIIAAGYE
FAHNDDYDRTLPDLKEGTLLFDDASSYELEAFVKALKPDLIGSGIKEKYIFQKMGVPFRQMH
SWDYSGPYHGYDGFAIFARDMDMTLNNPAWNELTAPWLKSGGYPYDVPDYA

>SN119
MMAMAVFRREGRRLLPSIAARPIAAIRSPLSSDQEEGLLGVRSISTQVVRNRGGMMTNATGE
RNLALIQEVLEVFPETARKERRKHMMVSDPEMESVGKCIISNRKSQPGVMTVRGCAYAGSKG
VVFGPIKDMAHISHGPVGCGQYSRAGRRNKYTGVSGVDSFGTLNFTSDFQERDIVFGGDKKL
SKLIEEMELLFPLTKGITIQSECPVGLIGDDISAVANASSKALDKPVIPVRCEGFRGVSQSL
GHHIANDVVRDWILNNREGQPFETTPYDVAIIGDYNIGGDAWASRILLEEMGLRVVAQWSGD
GTLVEMENTPFVKLNLVHCYRSMNYIARHMEEKHQIPWMEYNFFGPTKIAESLRKIADQFDD
TIRANAEAVIARYEGQMAAIIAKYRPRLEGRKVLLYMGGLRPRHVIGAYEDLGMEIIAAGYE
FAHNDDYDRTLPDLKEGTLLFDDASSYELEAFVKALKPDLIGSGIKEKYIFQKMGVPFRQMH
SWDYSGPYHGYDGFAIFARDMDMTLNNPAWNELTAPWLKSGGYPYDVPDYA

>SN120
MMAMAVFRREGRRLLPSIAARPIAAIRSPLSSDQEEGLLGVRSISTQVVRNRGGMMTNATGE
RNLALIQEVLEVFPETARKERRKHMMVSDPEMESVGKCIISNRKSQPGVMTVRGCAYAGSKG
VVFGPIKDMAHISHGPVGCGQYSRAGRRNKATGVSGVDSFGTLNFTSDFQERDIVFGGDKKL
SKLIEEMELLFPLTKGITIQSECPVGLIGDDISAVANASSKALDKPVIPVRCEGFRGVSQSL
GHHIANDVVRDWILNNREGQPFETTPYDVAIIGDYNIGGDAWASRILLEEMGLRVVAQWSGD
GTLVEMENTPFVKLNLVHCYRSMNYIARHMEEKHQIPWMEYNFFGPTKIAESLRKIADQFDD
TIRANAEAVIARYEGQMAAIIAKYRPRLEGRKVLLYMGGLRPRHVIGAYEDLGMEIIAAGYE
FAHNDDYDRTLPDLKEGTLLFDDASSYELEAFVKALKPDLIGSGIKEKYIFQKMGVPFRQMH
SWDYSGPYHGYDGFAIFARDMDMTLNNPAWNELTAPWLKSGGYPYDVPDYA

>SN121
MMAMAVFRREGRRLLPSIAARPIAAIRSPLSSDQEEGLLGVRSISTQVVRNRGGMMTNATGE
RNLALIQEVLEVFPETARKERRKHMMVSDPEMESVGKCIISNRKSQPGVMTVRGCAYAGSKG
VVFGPIKDMAHISHGPVGCGQYSRAGRRNYATGVSGVDSFGTLNFTSDFQERDIVFGGDKKL
SKLIEEMELLFPLTKGITIQSECPVGLIGDDISAVANASSKALDKPVIPVRCEGFRGVSQSL
GHHIANDVVRDWILNNREGQPFETTPYDVAIIGDYNIGGDAWASRILLEEMGLRVVAQWSGD
GTLVEMENTPFVKLNLVHCYRSMNYIARHMEEKHQIPWMEYNFFGPTKIAESLRKIADQFDD
TIRANAEAVIARYEGQMAAIIAKYRPRLEGRKVLLYMGGLRPRHVIGAYEDLGMEIIAAGYE
FAHNDDYDRTLPDLKEGTLLFDDASSYELEAFVKALKPDLIGSGIKEKYIFQKMGVPFRQMH
SWDYSGPYHGYDGFAIFARDMDMTLNNPAWNELTAPWLKSGGYPYDVPDYA

>SN122
MMAMAVFRREGRRLLPSIAARPIAAIRSPLSSDQEEGLLGVRSISTQVVRNRGGMMTNATGE
RNLALIQEVLEVFPETARKERRKHMMVSDPEMESVGKCIISNRKSQPGVMTVRGCAYAGSKG
VVFGPIKDMAHISHGPVGCGQYSRAGRKNYYTGVSGVDSFGTLNFTSDFQERDIVFGGDKKL
SKLIEEMELLFPLTKGITIQSECPVGLIGDDISAVANASSKALDKPVIPVRCEGFRGVSQSL
GHHIANDVVRDWILNNREGQPFETTPYDVAIIGDYNIGGDAWASRILLEEMGLRVVAQWSGD
GTLVEMENTPFVKLNLVHCYRSMNYIARHMEEKHQIPWMEYNFFGPTKIAESLRKIADQFDD
TIRANAEAVIARYEGQMAAIIAKYRPRLEGRKVLLYMGGLRPRHVIGAYEDLGMEIIAAGYE
FAHNDDYDRTLPDLKEGTLLFDDASSYELEAFVKALKPDLIGSGIKEKYIFQKMGVPFRQMH
SWDYSGPYHGYDGFAIFARDMDMTLNNPAWNELTAPWLKSGGYPYDVPDYA

>SN123
MMAMAVFRREGRRLLPSIAARPIAAIRSPLSSDQEEGLLGVRSISTQVVRNRGGMMTNATGE
RNLALIQEVLEVFPETARKERRKHMMVSDPEMESVGKCIISNRKSQPGVMTVRGCAYAGSKG
VVFGPIKDMAHISHGPVGCGQYSRAGRKNYFTGVSGVDSFGTLNFTSDFQERDIVFGGDKKL
SKLIEEMELLFPLTKGITIQSECPVGLIGDDISAVANASSKALDKPVIPVRCEGFRGVSQSL
GHHIANDVVRDWILNNREGQPFETTPYDVAIIGDYNIGGDAWASRILLEEMGLRVVAQWSGD
GTLVEMENTPFVKLNLVHCYRSMNYIARHMEEKHQIPWMEYNFFGPTKIAESLRKIADQFDD
TIRANAEAVIARYEGQMAAIIAKYRPRLEGRKVLLYMGGLRPRHVIGAYEDLGMEIIAAGYE
FAHNDDYDRTLPDLKEGTLLFDDASSYELEAFVKALKPDLIGSGIKEKYIFQKMGVPFRQMH
SWDYSGPYHGYDGFAIFARDMDMTLNNPAWNELTAPWLKSGGYPYDVPDYA

>SN124
MMAMAVFRREGRRLLPSIAARPIAAIRSPLSSDQEEGLLGVRSISTQVVRNRGGMMTNATGE
RNLALIQEVLEVFPETARKERRKHMMVSDPEMESVGKCIISNRKSQPGVMTVRGCAYAGSKG
VVFGPIKDMAHISHGPVGCGQYSRAGRKNYYAGVSGVDSFGTLNFTSDFQERDIVFGGDKKL
SKLIEEMELLFPLTKGITIQSECPVGLIGDDISAVANASSKALDKPVIPVRCEGFRGVSQSL
GHHIANDVVRDWILNNREGQPFETTPYDVAIIGDYNIGGDAWASRILLEEMGLRVVAQWSGD
GTLVEMENTPFVKLNLVHCYRSMNYIARHMEEKHQIPWMEYNFFGPTKIAESLRKIADQFDD
TIRANAEAVIARYEGQMAAIIAKYRPRLEGRKVLLYMGGLRPRHVIGAYEDLGMEIIAAGYE
FAHNDDYDRTLPDLKEGTLLFDDASSYELEAFVKALKPDLIGSGIKEKYIFQKMGVPFRQMH
SWDYSGPYHGYDGFAIFARDMDMTLNNPAWNELTAPWLKSGGYPYDVPDYA

>SN125
MMAMAVFRREGRRLLPSIAARPIAAIRSPLSSDQEEGLLGVRSISTQVVRNRGGMMTNATGE
RNLALIQEVLEVFPETARKERRKHMMVSDPEMESVGKCIISNRKSQPGVMTVRGCAYAGSKG
VVFGPIKDMAHISHGPVGCGQYSRAGRKNYFAGVSGVDSFGTLNFTSDFQERDIVFGGDKKL
SKLIEEMELLFPLTKGITIQSECPVGLIGDDISAVANASSKALDKPVIPVRCEGFRGVSQSL
GHHIANDVVRDWILNNREGQPFETTPYDVAIIGDYNIGGDAWASRILLEEMGLRVVAQWSGD
GTLVEMENTPFVKLNLVHCYRSMNYIARHMEEKHQIPWMEYNFFGPTKIAESLRKIADQFDD

TIRANAEAVIARYEGQMAAIIAKYRPRLEGRKVLLYMGGLRPRHVIGAYEDLGMEIIAAGYE
FAHNDDYDRTLPDLKEGTLLFDDASSYELEAFVKALKPDLIGSGIKEKYIFQKMGVPFRQMH
SWDYSGPYHGYDGFAIFARDMDMTLNNPAWNELTAPWLKSGGYPYDVPDYA

>SN126
MMAMAVFRREGRRLLPSIAARPIAAIRSPLSSDQEEGLLGVRSISTQVVRNRGGMMTNATGE
RNLALIQEVLEVFPETARKERRKHMMVSDPEMESVGKCIISNRKSQPGVMTVRGCAYAGSKG
VVFGPIKDMAHISHGPVGCGQYSRAGRKNYAAGVSGVDSFGTLNFTSDFQERDIVFGGDKKL
SKLIEEMELLFPLTKGITIQSECPVGLIGDDISAVANASSKALDKPVIPVRCEGFRGVSQSL
GHHIANDVVRDWILNNREGQPFETTPYDVAIIGDYNIGGDAWASRILLEEMGLRVVAQWSGD
GTLVEMENTPFVKLNLVHCYRSMNYIARHMEEKHQIPWMEYNFFGPTKIAESLRKIADQFDD
TIRANAEAVIARYEGQMAAIIAKYRPRLEGRKVLLYMGGLRPRHVIGAYEDLGMEIIAAGYE
FAHNDDYDRTLPDLKEGTLLFDDASSYELEAFVKALKPDLIGSGIKEKYIFQKMGVPFRQMH
SWDYSGPYHGYDGFAIFARDMDMTLNNPAWNELTAPWLKSGGYPYDVPDYA

>SN140
MAMAVFRREGRRLLPSIAARPIAAIRSPLSSDQEEGLLGVRSISTQVVRNRGGYPYDVPDYA
GGMSQTIDKINSCYPLFEQDEYQELFRNKRQLEEAHDAQRVQEVFAWTTTAEYEALNFQREA
LTVDPAKACQPLGAVLCSLGFANTLPYVHGSQGCVAYFRTYFNRHFKEPIACVSDSMTEDAA
VFGGNNNMNLGLQNASALYKPEIIAVSTTCMAEVIGDDLQAFIANAKKDGFVDSSIAVPHAH
TPSFIGSHVTGWDNMFEGFAKTFTADYQGQPGKLPKLNLVTGFETYLGNFRVLKRMMEQMAV
PCSLLSDPSEVLDTPADGHYRMYSGGTTQQEMKEAPDAIDTLLLQPWQLLKSKKVVQEMWNQ
PATEVAIPLGLAATDELLMTVSQLSGKPIADALTLERGRLVDMMLDSHTWLHGKKFGLYGDP
DFVMGLTRFLLELGCEPTVILSHNANKRWQKAMNKMLDASPYGRDSEVFINCDLWHFRSLMF
TRQPDFMIGNSYGKFIQRDTLAKGKAFEVPLIRLGFPLFDRHHLHRQTTWGYEGAMNIVTTL
VNAVLEKLDSDTSQLGKTDYSFDLVR

>SN159
MMAMAVFRREGRRLLPSIAARPIAAIRSPLSSDQEEGLLGVRSISTQVVRNRGGMMTNATGER
NLALIQEVLEVFPETARKERRKHMMVSDPEMESVGKCIISNRKSQPGVMTVRGCAYAGSKGV
VFGPIKDMAHISHGPVGCGQYSRAGRRNQYTGVSGVDSFGTLNFTSDFQERDIVFGGDKKLS
KLIEEMELLFPLTKGITIQSECPVGLIGDDISAVANASSKALDKPVIPVRCEGFRGVSQSLG
HHIANDVVRDWILNNREGQPFETTPYDVAIIGDYNIGGDAWASRILLEEMGLRVVAQWSGDG
TLVEMENTPFVKLNLVHCYRSMNYIARHMEEKHQIPWMEYNFFGPTKIAESLRKIADQFDDT
IRANAEAVIARYEGQMAAIIAKYRPRLEGRKVLLYMGGLRPRHVIGAYEDLGMEIIAAGYEF
AHNDDYDRTLPDLKEGTLLFDDASSYELEAFVKALKPDLIGSGIKEKYIFQKMGVPFRQMHS
WDYSGPYHGYDGFAIFARDMDMTLNNPAWNELTAPWLKSAATPPPGSTTTAYPYDVPDYATP
PPGSTTTASQTIDKINSCYPLFEQDEYQELFRNKRQLEEAHDAQRVQEVFAWTTTAEYEALN
FQREALTVDPAKACQPLGAVLCSLGFANTLPYVHGSQGCVAYFRTYFNRHFKEPIACVSDSM
TEDAAVFGGNNNMNLGLQNASALYKPEIIAVSTTCMAEVIGDDLQAFIANAKKDGFVDSSIA
VPHAHTPSFIGSHVTGWDNMFEGFAKTFTADYQGQPGKLPKLNLVTGFETYLGNFRVLKRMM
EQMAVPCSLLSDPSEVLDTPADGHYRMYSGGTTQQEMKEAPDAIDTLLLQPWQLLKSKKVVQ
EMWNQPATEVAIPLGLAATDELLMTVSQLSGKPIADALTLERGRLVDMMLDSHTWLHGKKFG
LYGDPDFVMGLTRFLLELGCEPTVILSHNANKRWQKAMNKMLDASPYGRDSEVFINCDLWHF
RSLMFTRQPDFMIGNSYGKFIQRDTLAKGKAFEVPLIRLGFPLFDRHHLHRQTTWGYEGAMN
IVTTLVNAVLEKLDSDTSQLGKTDYSFDLVR

>SN160
MMAMAVFRREGRRLLPSIAARPIAAAAAAAASSDQEEGLLAAAAAAAAVVRNRGGMTNATGER
NLALIQEVLEVFPETARKERRKHMMVSDPEMESVGKCIISNRKSQPGVMTVRGCAYAGSKGV

VFGPIKDMAHISHGPVGCGQYSRAGRRNQYTGVSGVDSFGTLNFTSDFQERDIVFGGDKKLS
KLIEEMELLFPLTKGITIQSECPVGLIGDDISAVANASSKALDKPVIPVRCEGFRGVSQSLG
HHIANDVVRDWILNNREGQPFETTPYDVAIIGDYNIGGDAWASRILLEEMGLRVVAQWSGDG
TLVEMENTPFVKLNLVHCYRSMNYIARHMEEKHQIPWMEYNFFGPTKIAESLRKIADQFDDT
IRANAEAVIARYEGQMAAIIAKYRPRLEGRKVLLYMGGLRPRHVIGAYEDLGMEIIAAGYEF
AHNDDYDRTLPDLKEGTLLFDDASSYELEAFVKALKPDLIGSGIKEKYIFQKMGVPFRQMHS
WDYSGPYHGYDGFAIFARDMDMTLNNPAWNELTAPWLKSAATPPPGSTTTAYPYDVPDYATP
PPGSTTTASQTIDKINSCYPLFEQDEYQELFRNKRQLEEAHDAQRVQEVFAWTTTAEYEALN
FQREALTVDPAKACQPLGAVLCSLGFANTLPYVHGSQGCVAYFRTYFNRHFKEPIACVSDSM
TEDAAVFGGNNNMNLGLQNASALYKPEIIAVSTTCMAEVIGDDLQAFIANAKKDGFVDSSIA
VPHAHTPSFIGSHVTGWDNMFEGFAKTFTADYQGQPGKLPKLNLVTGFETYLGNFRVLKRMM
EQMAVPCSLLSDPSEVLDTPADGHYRMYSGGTTQQEMKEAPDAIDTLLLQPWQLLKSKKVVQ
EMWNQPATEVAIPLGLAATDELLMTVSQLSGKPIADALTLERGRLVDMMLDSHTWLHGKKFG
LYGDPDFVMGLTRFLLELGCEPTVILSHNANKRWQKAMNKMLDASPYGRDSEVFINCDLWHF
RSLMFTRQPDFMIGNSYGKFIQRDTLAKGKAFEVPLIRLGFPLFDRHHLHRQTTWGYEGAMN
IVTTLVNAVLEKLDSDTSQLGKTDYSFDLVR

>SN176
MHHHHHHGGMTNATGERNLALIQEVLEVFPETARKERRKHMMVSDPEMESVGKCIISNRKSQ
PGVMTVRGCAYAGSKGVVFGPIKDMAHISHGPVGCGQYSRAGRRNYYTGVSGVDSFGTLNFT
SDFQERDIVFGGDKKLSKLIEEMELLFPLTKGITIQSECPVGLIGDDISAVANASSKALDKP
VIPVRCEGFRGVSQSLGHHIANDVVRDWILNNREGQPFETTPYDVAIIGDYNIGGDAWASRI
LLEEMGLRVVAQWSGDGTLVEMENTPFVKLNLVHCYRSMNYIARHMEEKHQIPWMEYNFFGP
TKIAESLRKIADQFDDTIRANAEAVIARYEGQMAAIIAKYRPRLEGRKVLLYMGGLRPRHVI
GAYEDLGMEIIAAGYEFAHNDDYDRTLPDLKEGTLLFDDASSYELEAFVKALKPDLIGSGIK
EKYIFQKMGVPFRQMHSWDYSGPYHGYDGFAIFARDMDMTLNNPAWNELTAPWLKSAATPPP
GSTTTAYPYDVPDYATPPPGSTTTASQTIDKINSCYPLFEQDEYQELFRNKRQLEEAHDAQR
VQEVFAWTTTAEYEALNFQREALTVDPAKACQPLGAVLCSLGFANTLPYVHGSQGCVAYFRT
YFNRHFKEPIACVSDSMTEDAAVFGGNNNMNLGLQNASALYKPEIIAVSTTCMAEVIGDDLQ
AFIANAKKDGFVDSSIAVPHAHTPSFIGSHVTGWDNMFEGFAKTFTADYQGQPGKLPKLNLV
TGFETYLGNFRVLKRMMEQMAVPCSLLSDPSEVLDTPADGHYRMYSGGTTQQEMKEAPDAID
TLLLQPWQLLKSKKVVQEMWNQPATEVAIPLGLAATDELLMTVSQLSGKPIADALTLERGRL
VDMMLDSHTWLHGKKFGLYGDPDFVMGLTRFLLELGCEPTVILSHNANKRWQKAMNKMLDAS
PYGRDSEVFINCDLWHFRSLMFTRQPDFMIGNSYGKFIQRDTLAKGKAFEVPLIRLGFPLFD
RHHLHRQTTWGYEGAMNIVTTLVNAVLEKLDSDTSQLGKTDYSFDLVR

>SN196, SNY196
MHHHHHHGGMMTNATGERNLALIQEVLEVFPETARKERRKHMMVSDPEMESVGKCIISNRKS
QPGVMTVRGCAYAGSKGVVFGPIKDMAHISHGPVGCGQYSRAGRRNYYTGVSGVDSFGTLNF
TSDFQERDIVFGGDKKLSKLIEEMELLFPLTKGITIQSECPVGLIGDDISAVANASSKALDK
PVIPVRCEGFRGVSQSLGHHIANDVVRDWILNNREGQPFETTPYDVAIIGDYNIGGDAWASR
ILLEEMGLRVVAQWSGDGTLVEMENTPFVKLNLVHCYRSMNYIARHMEEKHQIPWMEYNFFG
PTKIAESLRKIADQFDDTIRANAEAVIARYEGQMAAIIAKYRPRLEGRKVLLYMGGLRPRHV
IGAYEDLGMEIIAAGYEFAHNDDYDRTLPDLKEGTLLFDDASSYELEAFVKALKPDLIGSGI
KEKYIFQKMGVPFRQMHSWDYSGPYHGYDGFAIFARDMDMTLNNPAWNELTAPWLKSGGYPY
DVPDYA

>SN197
MWSHPQFEKGGGSGGGSGGSAWSHPQFEKGMVSKGEELFTGVVPILVELDGDVNGHKFSVSG
EGEGDATYGKLTLKFICTTGKLPVPWPTLVTTLSWGVQCFARYPDHMKQHDFFKSAMPEGYV
QERTIFFKDDGNYKTRAEVKFEGDTLVNRIELKGIDFKEDGNILGHKLEYNYFSDNVYITAD

KQKNGIKANFKIRHNIEDGGVQLADHYQQNTPIGDGPVLLPDNHYLSTQSKLSKDPNEKRDH
MVLLEFVTAAGITLGMDELYKGGGGENLYFQGGGMEEAKEREKLTLVTRKSSFGLPTSCPNC
LPVYLYLKFSKTPFDLAFNLINPDFGQIPYVESGTYVAYNNEKGGVIRSLIEDGFVDLDSQV
HGIPEWVSTKAMVDSWLADAILYELWVGSDGSSAHKIYFSDLPWPLGKLLYLKQVHVAKQIL
DITKDNAERREEEIYRNANDAFSALSTRLGEQAYLFDNRPTSLDAVFLGHALFTLYALPENS
VLRNKLLEHDNLVRYTEKHKLELVDSSASSSSGTQSQSDPSSVPRRPSQWSSKPKSKPKREK
TEEEKKFRRRAKYFLVTQLVAVLVFLSLLGGSGDAEVELDEDDYE

>SN221
MMAMAVFRREGRRLLPSIAARPIAAIRSPLSSDQEEGLLGVRSISTQVVRNRGGMAINEKVL
DEILSQYPTKVKKNRKKHIIIKDPNQARQEIEANTRTIPGIITNRGCAYAGCKGVVLGPLKD
VVHITHGPIGCGYYSWLTRRNKAASSDPTKNFISYCFSTDMQESDIVFGGEKKLARMIDEVM
EIFKPNAITISATCPVGLIGDDLGAVAKAAEQKHGITVMHFNCEGYKGVSQSAGHHIANNTL
MERVIGAGELEAAPGRYPINILGEYNIGGDSWEIERILREIGYTVLSVMTGDGSYEELKNAH
VAELNLVQCHRSINYIAEMLETKYGTPWLKVNFIGIQSTIDSLRNMAIYFGDPELTRRTEEV
IAKELAEVEPVMEQYKKICQGKTAFCFVGGSRGHHYQGLFAELGMETVLAGYEFAHRDDYEG
RDVLPQIKLDADNKNIPELHVEPDQRRFKLKVPRERMEELKKKIPLSYYAGMMVDMKGGHVV
VDDLNHYETEQFIKLLKPDIFASGIKDKYVVQKMGIPAKQLHSYDYSGPYAGFKGAVKFAED
ITMSFISPTWNFITPPWKNQPILEGEIVEGGCSTCGGYPYDVPDYA

>SN222
MMAMAVFRREGRRLLPSIAARPIAAIRSPLSSDQEEGLLGVRSISTQVVRNRGGMSLDYEND
SALHQELITQVLSQYPHKAAKRRQKHLSVASDREAVGEEGETLSECDVKSNIKSIPGVMTIR
GCAYAGSKGVVWGPVKDMVHISHGPVGCGQYSWSQRRNYYVGTTGVDTFVTMQFTSDFQEKD
IVFGGDKKLEQVIDEIEELFPLNNGITIQSECPIGLIGDDIEAVSRKKAAEHETTIVPVRCE
GFRGVSQSLGHHIANDAIRDWVFDKADGKTDVEFETGPYDVNVIGDYNIGGDAWASRILLEE
IGLRVVGNWSGDATLAEVERAPRAKLNLIHCYRSMNYICRHMEERYAIPWMEYNFFGPSQIE
ASLRKIARHFGPTIEERAERVIAKYRPLVDAVIDKYWPRLQGKRVMLYVGGLRPRHVITAYE
DLGMQIVGTGYEFAHNDDYQRTGHYVKTGTLIYDDATSYELDTFIERIRPDLVGSGIKEKYP
VQKMGIPFRQMHSWDYSGPYHGYDGFAIFARDMDLAINNPVWDLYDAPWKKMTVPTAAVAAE
GGYPYDVPDYA

>SN223
MMAMAVFRREGRRLLPSIAARPIAAIRSPLSSDQEEGLLGVRSISTQVVRNRGGMTGMSREE
VESLIQEVLEVYPEKARKDRNKHLAVNDPAVTQSKKCIISNKKSQPGLMTIRGCAYAGSKGV
VWGPIKDMIHISHGPVGCGQYSRAGRRNYYIGTTGVNAFVTMNFTSDFQEKDIVFGGDKKLA
KLIDEVETLFPLNKGISVQSECPIGLIGDDIESVSKVKGAELSKTIVPVRCEGFRGVSQSLG
HHIANDAVRDWVLGKRDEDTTFASTPYDVAIIGDYNIGGDAWSSRILLEEMGLRCVAQWSGD
GSISEIELTPKVKLNLVHCYRSMNYISRHMEEKYGIPWMEYNFFGPTKTIESLRAIAAKFDE
SIQKKCEEVIAKYKPEWEAVVAKYRPRLEGKRVMLYIGGLRPRHVIGAYEDLGMEVVGTGYE
FAHNDDYDRTMKEMGDSTLLYDDVTGYEFEEFVKRIKPDLIGSGIKEKFIFQKMGIPFRQMH
SWDYSGPYHGFDGFAIFARDMDMTLNNPCWKKLQAPWEASEGAEKVAASAGGYPYDVPDYA

>SN224
MMAMAVFRREGRRLLPSIAARPIAAIRSPLSSDQEEGLLGVRSISTQVVRNRGGMALKHKSI
PDVATVKEELLKKYPTKVARKRAKQIVINDVKDGDVVPEVQANVRTTPGIITMRGCTYAGCK
GVILGPTRDIVNITHGPIGCGFYSWLTRRNQTKAPLESSENFMPYAFSTDMQDEDIIFGGEK
KLIAAIQEAYDTFHPKAIAIFATCPVGLIGDDIHAVARKMKEKLGINIFAFSCEGYKGVSQS
AGHHIANNQIFTHVVGEDDTPKLGEYKINMLGEYNIGGDAFELERVLEKCGITLVSTFSGNS
TYEHFATAHQADLNAVMCHRSINYVAEMMETKYGIPWIKVNFIGAESSAKSLRKIAQYFGDK
KLIDRVEEVIAEEMPAVHAALEDVKPFTEGKTAMLFVGGSRAHHYQDLFTEMGMKTIAAGYE

FAHRDDYEGRKVMPTIKVDADSRNIEEIEVTPDSTRFVPRKSDEDLKRLAEAGFTFKDYEGM
MPQMESDTLVIDDLNQYEADKLIELLKPDVFCAGIKEKFSVQKMGVPMKQLHSYDYGGPYAG
FKGAVNFYTEIKRLVTSKVWSDLKAPWEENPELSATYVWEGGYPYDVPDYA

>SN225
MMAMAVFRREGRRLLPSIAARPIAAIRSPLSSDQEEGLLGVRSISTQVVRNRGGMEAKVLIP
DPSKIKEELINKYPAKVAKKRSKSIVVNDPEIVPEVQANVRTVPGIITQRGCAYAGCKGVVL
GPTRDIVNIVHGPIGCSFYAWLTRRNQTRPETPEHENYITYCFSTDMQEEHVVFGGEKKLKV
AIQEAYDLFHPKAIAIFSTCPVGLIGDDVHAVAREMKEKLGDCNVFGFSCEGYRGVSQSAGH
HIANNGVFKHMVGNNNEVKPGKFKLNLLGEYNIGGDAFEIERLLEKCGITLVASFSGNSTVG
AIENAHTADLNVIMCHRSINYMGDMMETKYGIPWMKVNFVGAESTAKSLRKIAEYFGDEELK
AKVEEVIAEEVPAVKAIIDEIRPRTEGKTAMLFVGGSRAHHYQDLFSELGMTTIAAGYEFAH
RDDYEGREVLPKIKIDADSKNIEELKVTADPELYNPRKSKAELEELKAKGLEINGYEGMMKQ
MMKKTLVVDDISHYESEKLIEMYKPDIFCAGIKEKYVVQKMGVPLKQLHSYDYGGPYTGFKG
AVNFYKDIDRMVNNPVWKMIKAPWEKSEPESLEASYVASGGYPYDVPDYA

>SN226
MMAMAVFRREGRRLLPSIAARPIAAIRSPLSSDQEEGLLGVRSISTQVVRNRGGMSLSVNEG
VDVKGLVDKVLEAYPEKSRKRRAKHLNVLEAEAKDCGVKSNIKSIPGVMTIRGCAYAGSKGV
VWGPIKDMIHISHGPVGCGYYSWSGRRNYYVGDTGVDSWGTMHFTSDFQEKDIVFGGDKKLH
KVIEEINELFPLVNGISIQSECPIGLIGDDIEAVARAKSEELGKPVVPVRCEGFRGVSQSLG
HHIANDVIRDWIFEKTEPKEGFVSTPYDVTIIGDYNIGGDAWASRILLEEIGLRVIAQWSGD
GTLAELENTPKAKVNLIHCYRSMNYIARHMEEKFGIPWMEYNFFGPSQIAESLRKIAALFDD
TIKENAEKVIAKYQPMVDAVIAKFKPRLEGKKVMIYVGGLRPRHVVDAYHDLGMEIVGTGYE
FAHNDDYQRTQHYVKEGTLIYDDVTAFELEKFVEVMRPDLVASGIKEKYVFQKMGLPFRQMH
SWDYSGPYHGYDGFAIFARDMDLAINNPVWGIMKAPFGGYPYDVPDYA

>SN235
MAMAVFRREAAALLPSIAARPIAAAAAAASSDQEEGLLAAAAAAAAVVRNRGGYPYDVPDYA
GGMAINEKVLDEILSQYPTKVKKNRKKHIIIKDPNQARQEIEANTRTIPGIITNRGCAYAGC
KGVVLGPLKDVVHITHGPIGCGYYSWLTRRNKAASSDPTKNFISYCFSTDMQESDIVFGGEK
KLARMIDEVMEIFKPNAITISATCPVGLIGDDLGAVAKAAEQKHGITVMHFNCEGYKGVSQS
AGHHIANNTLMERVIGAGELEAAPGRYPINILGEYNIGGDSWEIERILREIGYTVLSVMTGD
GSYEELKNAHVAELNLVQCHRSINYIAEMLETKYGTPWLKVNFIGIQSTIDSLRNMAIYFGD
PELTRRTEEVIAKELAEVEPVMEQYKKICQGKTAFCFVGGSRGHHYQGLFAELGMETVLAGY
EFAHRDDYEGRDVLPQIKLDADNKNIPELHVEPDQRRFKLKVPRERMEELKKKIPLSYYAGM
MVDMKGGHVVVDDLNHYETEQFIKLLKPDIFASGIKDKYVVQKMGIPAKQLHSYDYSGPYAG
FKGAVKFAEDITMSFISPTWNFITPPWKNQPILEGEIVEGGCSTCGGYPYDVPDYA

>SN236
MAMAVFRREAAALLPSIAARPIAAAAAAASSDQEEGLLAAAAAAAAVVRNRGGMSLDYENDS
ALHQELITQVLSQYPHKAAKRRQKHLSVASDREAVGEEGETLSECDVKSNIKSIPGVMTIRG
CAYAGSKGVVWGPVKDMVHISHGPVGCGQYSWSQRRNYYVGTTGVDTFVTMQFTSDFQEKDI
VFGGDKKLEQVIDEIEELFPLNNGITIQSECPIGLIGDDIEAVSRKKAAEHETTIVPVRCEG
FRGVSQSLGHHIANDAIRDWVFDKADGKTDVEFETGPYDVNVIGDYNIGGDAWASRILLEEI
GLRVVGNWSGDATLAEVERAPRAKLNLIHCYRSMNYICRHMEERYAIPWMEYNFFGPSQIEA
SLRKIARHFGPTIEERAERVIAKYRPLVDAVIDKYWPRLQGKRVMLYVGGLRPRHVITAYED
LGMQIVGTGYEFAHNDDYQRTGHYVKTGTLIYDDATSYELDTFIERIRPDLVGSGIKEKYPV
QKMGIPFRQMHSWDYSGPYHGYDGFAIFARDMDLAINNPVWDLYDAPWKKMTVPTAAVAAEG
GYPYDVPDYA

>SN237
MAMAVFRREAAALLPSIAARPIAAAAAAASSDQEEGLLAAAAAAAAVVRNRGGMTGMSREEV
ESLIQEVLEVYPEKARKDRNKHLAVNDPAVTQSKKCIISNKKSQPGLMTIRGCAYAGSKGVV
WGPIKDMIHISHGPVGCGQYSRAGRRNYYIGTTGVNAFVTMNFTSDFQEKDIVFGGDKKLAK
LIDEVETLFPLNKGISVQSECPIGLIGDDIESVSKVKGAELSKTIVPVRCEGFRGVSQSLGH
HIANDAVRDWVLGKRDEDTTFASTPYDVAIIGDYNIGGDAWSSRILLEEMGLRCVAQWSGDG
SISEIELTPKVKLNLVHCYRSMNYISRHMEEKYGIPWMEYNFFGPTKTIESLRAIAAKFDES
IQKKCEEVIAKYKPEWEAVVAKYRPRLEGKRVMLYIGGLRPRHVIGAYEDLGMEVVGTGYEF
AHNDDYDRTMKEMGDSTLLYDDVTGYEFEEFVKRIKPDLIGSGIKEKFIFQKMGIPFRQMHS
WDYSGPYHGFDGFAIFARDMDMTLNNPCWKKLQAPWEASEGAEKVAASAGGYPYDVPDYA

>SN238
MAMAVFRREAAALLPSIAARPIAAAAAAASSDQEEGLLAAAAAAAAVVRNRGGMALKHKSIP
DVATVKEELLKKYPTKVARKRAKQIVINDVKDGDVVPEVQANVRTTPGIITMRGCTYAGCKG
VILGPTRDIVNITHGPIGCGFYSWLTRRNQTKAPLESSENFMPYAFSTDMQDEDIIFGGEKK
LIAAIQEAYDTFHPKAIAIFATCPVGLIGDDIHAVARKMKEKLGINIFAFSCEGYKGVSQSA
GHHIANNQIFTHVVGEDDTPKLGEYKINMLGEYNIGGDAFELERVLEKCGITLVSTFSGNST
YEHFATAHQADLNAVMCHRSINYVAEMMETKYGIPWIKVNFIGAESSAKSLRKIAQYFGDKK
LIDRVEEVIAEEMPAVHAALEDVKPFTEGKTAMLFVGGSRAHHYQDLFTEMGMKTIAAGYEF
AHRDDYEGRKVMPTIKVDADSRNIEEIEVTPDSTRFVPRKSDEDLKRLAEAGFTFKDYEGMM
PQMESDTLVIDDLNQYEADKLIELLKPDVFCAGIKEKFSVQKMGVPMKQLHSYDYGGPYAGF
KGAVNFYTEIKRLVTSKVWSDLKAPWEENPELSATYVWEGGYPYDVPDYA

>SN239
MAMAVFRREAAALLPSIAARPIAAAAAAASSDQEEGLLAAAAAAAAVVRNRGGMEAKVLIPD
PSKIKEELINKYPAKVAKKRSKSIVVNDPEIVPEVQANVRTVPGIITQRGCAYAGCKGVVLG
PTRDIVNIVHGPIGCSFYAWLTRRNQTRPETPEHENYITYCFSTDMQEEHVVFGGEKKLKVA
IQEAYDLFHPKAIAIFSTCPVGLIGDDVHAVAREMKEKLGDCNVFGFSCEGYRGVSQSAGHH
IANNGVFKHMVGNNNEVKPGKFKLNLLGEYNIGGDAFEIERLLEKCGITLVASFSGNSTVGA
IENAHTADLNVIMCHRSINYMGDMMETKYGIPWMKVNFVGAESTAKSLRKIAEYFGDEELKA
KVEEVIAEEVPAVKAIIDEIRPRTEGKTAMLFVGGSRAHHYQDLFSELGMTTIAAGYEFAHR
DDYEGREVLPKIKIDADSKNIEELKVTADPELYNPRKSKAELEELKAKGLEINGYEGMMKQM
MKKTLVVDDISHYESEKLIEMYKPDIFCAGIKEKYVVQKMGVPLKQLHSYDYGGPYTGFKGA
VNFYKDIDRMVNNPVWKMIKAPWEKSEPESLEASYVASGGYPYDVPDYA

>SN240
MAMAVFRREAAALLPSIAARPIAAAAAAASSDQEEGLLAAAAAAAAVVRNRGGMSLSVNEGV
DVKGLVDKVLEAYPEKSRKRRAKHLNVLEAEAKDCGVKSNIKSIPGVMTIRGCAYAGSKGVV
WGPIKDMIHISHGPVGCGYYSWSGRRNYYVGDTGVDSWGTMHFTSDFQEKDIVFGGDKKLHK
VIEEINELFPLVNGISIQSECPIGLIGDDIEAVARAKSEELGKPVVPVRCEGFRGVSQSLGH
HIANDVIRDWIFEKTEPKEGFVSTPYDVTIIGDYNIGGDAWASRILLEEIGLRVIAQWSGDG
TLAELENTPKAKVNLIHCYRSMNYIARHMEEKFGIPWMEYNFFGPSQIAESLRKIAALFDDT
IKENAEKVIAKYQPMVDAVIAKFKPRLEGKKVMIYVGGLRPRHVVDAYHDLGMEIVGTGYEF
AHNDDYQRTQHYVKEGTLIYDDVTAFELEKFVEVMRPDLVASGIKEKYVFQKMGLPFRQMHS
WDYSGPYHGYDGFAIFARDMDLAINNPVWGIMKAPFGGYPYDVPDYA

>SN241
MHHHHHHGGMAINEKVLDEILSQYPTKVKKNRKKHIIIKDPNQARQEIEANTRTIPGIITNR
GCAYAGCKGVVLGPLKDVVHITHGPIGCGYYSWLTRRNKAASSDPTKNFISYCFSTDMQESD
IVFGGEKKLARMIDEVMEIFKPNAITISATCPVGLIGDDLGAVAKAAEQKHGITVMHFNCEG
YKGVSQSAGHHIANNTLMERVIGAGELEAAPGRYPINILGEYNIGGDSWEIERILREIGYTV

LSVMTGDGSYEELKNAHVAELNLVQCHRSINYIAEMLETKYGTPWLKVNFIGIQSTIDSLRN
MAIYFGDPELTRRTEEVIAKELAEVEPVMEQYKKICQGKTAFCFVGGSRGHHYQGLFAELGM
ETVLAGYEFAHRDDYEGRDVLPQIKLDADNKNIPELHVEPDQRRFKLKVPRERMEELKKKIP
LSYYAGMMVDMKGGHVVVDDLNHYETEQFIKLLKPDIFASGIKDKYVVQKMGIPAKQLHSYD
YSGPYAGFKGAVKFAEDITMSFISPTWNFITPPWKNQPILEGEIVEGGCSTCGGYPYDVPDY
A

>SN242
MHHHHHHGGMSLDYENDSALHQELITQVLSQYPHKAAKRRQKHLSVASDREAVGEEGETLSE
CDVKSNIKSIPGVMTIRGCAYAGSKGVVWGPVKDMVHISHGPVGCGQYSWSQRRNYYVGTTG
VDTFVTMQFTSDFQEKDIVFGGDKKLEQVIDEIEELFPLNNGITIQSECPIGLIGDDIEAVS
RKKAAEHETTIVPVRCEGFRGVSQSLGHHIANDAIRDWVFDKADGKTDVEFETGPYDVNVIG
DYNIGGDAWASRILLEEIGLRVVGNWSGDATLAEVERAPRAKLNLIHCYRSMNYICRHMEER
YAIPWMEYNFFGPSQIEASLRKIARHFGPTIEERAERVIAKYRPLVDAVIDKYWPRLQGKRV
MLYVGGLRPRHVITAYEDLGMQIVGTGYEFAHNDDYQRTGHYVKTGTLIYDDATSYELDTFI
ERIRPDLVGSGIKEKYPVQKMGIPFRQMHSWDYSGPYHGYDGFAIFARDMDLAINNPVWDLY
DAPWKKMTVPTAAVAAEGGYPYDVPDYA

>SN243
MHHHHHHGGMTGMSREEVESLIQEVLEVYPEKARKDRNKHLAVNDPAVTQSKKCIISNKKSQ
PGLMTIRGCAYAGSKGVVWGPIKDMIHISHGPVGCGQYSRAGRRNYYIGTTGVNAFVTMNFT
SDFQEKDIVFGGDKKLAKLIDEVETLFPLNKGISVQSECPIGLIGDDIESVSKVKGAELSKT
IVPVRCEGFRGVSQSLGHHIANDAVRDWVLGKRDEDTTFASTPYDVAIIGDYNIGGDAWSSR
ILLEEMGLRCVAQWSGDGSISEIELTPKVKLNLVHCYRSMNYISRHMEEKYGIPWMEYNFFG
PTKTIESLRAIAAKFDESIQKKCEEVIAKYKPEWEAVVAKYRPRLEGKRVMLYIGGLRPRHV
IGAYEDLGMEVVGTGYEFAHNDDYDRTMKEMGDSTLLYDDVTGYEFEEFVKRIKPDLIGSGI
KEKFIFQKMGIPFRQMHSWDYSGPYHGFDGFAIFARDMDMTLNNPCWKKLQAPWEASEGAEK
VAASAGGYPYDVPDYA

>SN244
MHHHHHHGGMALKHKSIPDVATVKEELLKKYPTKVARKRAKQIVINDVKDGDVVPEVQANVR
TTPGIITMRGCTYAGCKGVILGPTRDIVNITHGPIGCGFYSWLTRRNQTKAPLESSENFMPY
AFSTDMQDEDIIFGGEKKLIAAIQEAYDTFHPKAIAIFATCPVGLIGDDIHAVARKMKEKLG
INIFAFSCEGYKGVSQSAGHHIANNQIFTHVVGEDDTPKLGEYKINMLGEYNIGGDAFELER
VLEKCGITLVSTFSGNSTYEHFATAHQADLNAVMCHRSINYVAEMMETKYGIPWIKVNFIGA
ESSAKSLRKIAQYFGDKKLIDRVEEVIAEEMPAVHAALEDVKPFTEGKTAMLFVGGSRAHHY
QDLFTEMGMKTIAAGYEFAHRDDYEGRKVMPTIKVDADSRNIEEIEVTPDSTRFVPRKSDED
LKRLAEAGFTFKDYEGMMPQMESDTLVIDDLNQYEADKLIELLKPDVFCAGIKEKFSVQKMG
VPMKQLHSYDYGGPYAGFKGAVNFYTEIKRLVTSKVWSDLKAPWEENPELSATYVWEGGYPY
DVPDYA

>SN245
MHHHHHHGGMEAKVLIPDPSKIKEELINKYPAKVAKKRSKSIVVNDPEIVPEVQANVRTVPG
IITQRGCAYAGCKGVVLGPTRDIVNIVHGPIGCSFYAWLTRRNQTRPETPEHENYITYCFST
DMQEEHVVFGGEKKLKVAIQEAYDLFHPKAIAIFSTCPVGLIGDDVHAVAREMKEKLGDCNV
FGFSCEGYRGVSQSAGHHIANNGVFKHMVGNNNEVKPGKFKLNLLGEYNIGGDAFEIERLLE
KCGITLVASFSGNSTVGAIENAHTADLNVIMCHRSINYMGDMMETKYGIPWMKVNFVGAEST
AKSLRKIAEYFGDEELKAKVEEVIAEEVPAVKAIIDEIRPRTEGKTAMLFVGGSRAHHYQDL
FSELGMTTIAAGYEFAHRDDYEGREVLPKIKIDADSKNIEELKVTADPELYNPRKSKAELEE
LKAKGLEINGYEGMMKQMMKKTLVVDDISHYESEKLIEMYKPDIFCAGIKEKYVVQKMGVPL

KQLHSYDYGGPYTGFKGAVNFYKDIDRMVNNPVWKMIKAPWEKSEPESLEASYVASGGYPYD
VPDYA

>SN246
MHHHHHHGGMSLSVNEGVDVKGLVDKVLEAYPEKSRKRRAKHLNVLEAEAKDCGVKSNIKSI
PGVMTIRGCAYAGSKGVVWGPIKDMIHISHGPVGCGYYSWSGRRNYYVGDTGVDSWGTMHFT
SDFQEKDIVFGGDKKLHKVIEEINELFPLVNGISIQSECPIGLIGDDIEAVARAKSEELGKP
VVPVRCEGFRGVSQSLGHHIANDVIRDWIFEKTEPKEGFVSTPYDVTIIGDYNIGGDAWASR
ILLEEIGLRVIAQWSGDGTLAELENTPKAKVNLIHCYRSMNYIARHMEEKFGIPWMEYNFFG
PSQIAESLRKIAALFDDTIKENAEKVIAKYQPMVDAVIAKFKPRLEGKKVMIYVGGLRPRHV
VDAYHDLGMEIVGTGYEFAHNDDYQRTQHYVKEGTLIYDDVTAFELEKFVEVMRPDLVASGI
KEKYVFQKMGLPFRQMHSWDYSGPYHGYDGFAIFARDMDLAINNPVWGIMKAPFGGYPYDVP
DYA

>SN356
MAMAVFRREGRRLLPSIAARPIAAIRSPLSSDQEEGLLGVRSISTQVVRNRGGYPYDVPDYA
GGMPQSAEKILDHAPLFREPEYRQMLAEKKLNFECPHPERLVTDQREYSKGWEYREKNLARE
ALVVNPAKACQPLGAVFAAAGFERTMSFVHGSQGCVAYYRSHLSRHFKEPASAVSSSMTEDA
AVFGGLKNMVDGLANTYALYDPKMIAVSTTCMAEVIGDDLHGFIENAKSEGAVPPEFDVPFA
HTPAFVGSHVDGYDSMVKGILEHFWKGQARTQAAGTINIIPGFDGFCVGNNRELQRLLTLMG
VSYTFIQDASDQFDTPSDGEYRMYDGGTTIKALRAALNAEATLSLQHYNSRKTLEYCREVGQ
ATAAFHYPLGINATDAFLMKVSAISGREIPETIRLERGRLVDAMADSQSWLHGKTYAIYGDP
DFVYAMARFVMETGGEPRHCLATNGTAAWQAEMTELLASSPFGKQAKVWPGKDLWALRSLLF
TEPVDLLIGNSYGKYLERDTGTPLIRLMFPIFDRHHHHRFPLMGYQGGLRLLTTILDTIFDR
LDRETMQTAVTDYSYDLTR

>SN357
MAMAVFRREGRRLLPSIAARPIAAIRSPLSSDQEEGLLGVRSISTQVVRNRGGYPYDVPDYA
GGMLLRHTPTEIKERSALNINPAKTCQPIGAMYAGLGIKGCLPHSHGSQGCCAYHRSTLTRH
YKEPVSAATSSFTEGASVFGGQANLLQAIENIFSVYEPEIIAVHTTCLSETIGDDLNQIVDK
AVKEGKVPDGKQVIFASTPSYVGSHVTGFSNMVKGMVKCLAVSSGKKNGKVNIIPGWVEPAD
MEEIKRIAGMIGVSFTMFPDTSGVLNGPLTGEYHMFPDAGTPAEDIRAAGDAIGTLALGEWC
SADAARTLDSQCKVPCRVLDMPIGLKATDRFIDALRVVAGTSVPDTVNFERGQLIDVISDYH
QYFFGKKVALVGDPDQLIALTEFLLTLDMQPVHVVTGTPGKKFEARIRELCAGKGFDVNVRA
AGDMFLLHQWIKNEPVDLIMGNTYCKYIVRDEDIPYVRFGFPIMDRVGHQYFPVTGYKGGIR
LMEKILGVLLDRADRDAPEEKFELVY

>SN358
MAMAVFRREGRRLLPSIAARPIAAIRSPLSSDQEEGLLGVRSISTQVVRNRGGYPYDVPDYA
GGMLDCTPKEIKERTGGVINPAKTCQPIGAMYAALGIHRCLPHSHGSQGCCSYHRMHLSRHF
RDPIMASSSSFTEGSSVFGGGANLKTAIKNVFAIYNPDVMAVHTTCLSETIGDDLPTIIKGA
EIPEGKVVIHANTPSYQGSHITGFSNMVKGMVNYLSTASLETKKEQVNIIPGFVNPGDMREI
KRILKVMGIKFIMFPDTSGVVDSPMTGKYEMFPKGGTTLAELRDTGNSKVTIALGSYASGDA
AYQLERKCQVQPLIIKTPIGIKASDEFLMALMNKFTVDIPPELAEERGQLVDIMTDTHFHFH
GKKVAIFGDPDIVIAMTEFLLSLGMKPIHVLTGTPATGGAIGHTVGNFEQEVGEMLKQAGVA
GRVKAAGDLFELHQWIKNEPVDLLIGNTYGKYIAKAEDIPFVRLGFPILDRSVHSYLPVVGY
KGAMRLLEMISNALLDRADRDAKDQDFELVM

>SN384
MAMAVFRREGRRLLPSIAARPIAAIRSPLSSDQEEGLLGVRSISTQVVRNRGGYPYDVPDYA
GGMLLRHTTKEVKEREGLTINPAKTCQPIGAMYAALGIHGCLPHSHGSQGCCAYHRSTLTRH

YKEPVMAATSSFTEGASVFGGQANLLSAIETIFTVYDPEVIAVHSTCLSETIGDDLQQITKK
ASDDGKIPEGKYVIYASTPSYVGSHITGYANMVTSMTEQFAVSTGEKKDQVNVIAGWMEPSD
MREIKSLASRLGVKIVLFPDTSDVLDAPQTGKHEFYPKGGITINELKSAGDSKCSLAVGCIS
AEPAAIALEKKCKVPFETVDMPIGLSATDRFIMALSKAGSVKVPDEITAERGRLVDVMVDME
QYFYGKKVALFGDPDQLIPLTEFLLDLGMIPAHIVSGTPGLRFEKRMKEILERAPGANFRNG
PQADMFLMHQWIKNEPVDLLIGNTYGKYIARDEDIPFVRFGFPILDRIGHSYFPNVGYSGSL
RLVEKILGVLMDRQDRTSLEEKFELVMLSFSS

>SN385
MAMAVFRREGRRLLPSIAARPIAAIRSPLSSDQEEGLLGVRSISTQVVRNRGGYPYDVPDYA
GGMSHPVSQSADKVIDHFTLFRQPEYKELFERKKTEFEYGHSDEEVARVSAWTKTEEYKEKN
FAREAVVINPTKACQPIGAMFAAQGFEGTLPFVHGSQGCVAYYRTHLTRHFKEPNSAVSSSM
TEDAAVFGGLNNMIDGLANAYALYKPKMIAVLTTCMAEVIGDDLSGFINNAKNKESVPADFP
VPFAHTPAFVGSHIVGYDNMIKGVLTHFWGTSENFDTPKNEKINLIPGFDGFAVGNNRELKR
IAGLFGIDLTILSDVSDNFDTPADGEYRMYDGGTPLEATKEAVHAKATISMQEYCTPQSLQF
IKEKGQQVAKYNYPMGVTGTDELLLKLAELSGKPVPAELKLERGRLVDAIADSHTHLHGKRF
AVYGDPDFCLGMSKFLMELGAEPVHILSTSGSKKWEKQVQKVLDASPFGKSGKAYGGKDLWH
LRSLLFTDKVDYIIGNSYGKYLERDTKIPLIRLTYPIFDRHHHHRYPTWGYQGALNVLVRIL
DRIFEDMDANTNIVGETDYSFDLVR

>SN388
MAMAVFRREGRRLLPSIAARPIAAIRSPLSSDQEEGLLGVRSISTQVVRNRGGYPYDVPDYA
GGMLLRHTTKEVKEREGLTINPAKTCQPIGAMYAALGIHGCLPHSHGSQGCCAYHRSTLTRH
YKEPVMAATSSFTEGASVFGGQANLLSAIETIFTVYDPEVIAVHSTCLSETIGDDLQQITKK
ASDDGKIPEGKYVIYASTPSYVGSHITGYANMVTSMTEQFAVSTGEKKDQVNVIAGWMEPSD
MREIKSLASRLGVKIVLFPDTSDVLDAPQTGKHEFYPKGGITINELKSAGDSKCSLAVGCIS
AEPAAIALEKKCKVPFETVDMPIGLSATDRFIMALSKAGSVKVPDEITAERGRLVDVMVDME
QYFYGKKVALFGDPDQLIPLTEFLLDLGMIPAHIVSGTPGLRFEKRMKEILERAPGANFRNG
PQADMFLMHQWIKNEPVDLLIGNTYGKYIARDEDIPFVRFGFPILDRIGHSYFPNVGYSGSL
RLVEKILGVLMDRQDRTSLEEKFELVMLSFSS

>PRA19
MAMAVFRREGRRLLPSIAARPIAAIRSPLSSDQEEGLLGVRSISTQVVRNRMKSVKNIQKIT
KAMKMVAASKLRAVQGAPTNATGERNLALIQEVLEVFPETARKERRKHMMVSDPKMKSVGKC
IISNRKSQPGVMTVRGCAYAGSKGVVFGPIKDMAHISHGPAGCGQYSRAERRNYYTGVSGVD
SFGTLNFTSDFQERDIVFGGDKKLSKLIEEMELLFPLTKGITIQSECPVGLIGDDISAVANA
SSKALDKPVIPVRCEGFRGVSQSLGHHIANDVVRDWILNNREGQPFETTPYDVAIIGDYNIG
GDAWASRILLEEMGLRVVAQWSGDGTLVEMENTPFVKLNLVHCYRSMNYIARHMEEKHQIPW
MEYNFFGPTKIAESLRKIADQFDDTIRANAEAVIARYEGQMAAIIAKYRPRLEGRKVLLYIG
GLRPRHVIGAYEDLGMEIIAAGYEFAHNDDYDRTLPDLKEGTLLFDDASSYELEAFVKALKP
DLIGSGIKEKYIFQKMGVPFRQMHSWDYSGPYHGYDGFAIFARDMDMTLNNPAWNELTAPWL
KSAAGYPYDVPDYAPG

>PRA30
MAMAVFRREGRRLLPSIAARPIAAIRSPLSSDQEEGLLGVRSISTQVVRNRMKSVKNIQKIT
KAMKMVAASKLRAVQGAPTNATGERNLALIQEVLEVFPETARKERRKHLAVSDPKLKSVGKC
IISNRKSQPGVLTVRGCAYAGSKGVVFGPIKDLAHISHGPAGCGQYSRAERRNYYTGVSGVD
SFGTLNFTSDFQERDIVFGGDKKLSKLIEEMELLFPLTKGITIQSECPVGLIGDDISAVANA
SSKALDKPVIPVRCEGFRGVSQSLGHHIANDVVRDWILNNREGQPFETTPYDVAIIGDYNIG
GDAWASRILLEEMGLRVVAQWSGDGTLVEMENTPFVKLNLVHCYRSMNYIARHMEEKHQIPW
MEYNFFGPTKIAESLRKIADQFDDTIRANAEAVIARYEGQMAAIIAKYRPRLEGRKVLLYIG

GLRPRHVIGAYEDLGMEIIAAGYEFAHNDDYDRTLPDLKEGTLLFDDASSYELEAFVKALKP
DLIGSGIKEKYIFQKMGVPFRQMHSWDYSGPYHGYDGFAIFARDMDMTLNNPAWNELTAPWL
KSAAGYPYDVPDYAPG

Table S3. Frequency distribution of amino acid residues in 1476 putative NifD sequences around the secondary cleavage site.

***K. oxytoca* amino acid**	**R97**	**R98**	**N99**	**Y100**	**Y101**	**T102**
**position relative to cleavage site**	**-3**	**-2**	**-1**	**1**	**2**	**3**
most common	R, 1474 (99.86%)	R, 1472 (99.73%)	N, 1444 (97.83%)	Y, 1056 (71.54%)	Y, 951 (64.43%)	V, 402 (27.24%)
2nd most common	Gap, 2 (0.14%)	Gap, 2 (0.14%)	H, 17 (1.15%)	Q, 159 (10.77%)	A, 240 (16.26%)	I, 374 (25.34%)
3rd most common		C, 1 (0.07%)	F, 7 (0.47%)	L, 114 (7.72 %)	T, 148 (10.03%)	T, 147 (9.97%)
4th most common		G, 1 (0.07%)	A, 3 (0.20%)	K, 108 (7.32%)	M, 44 (2.98%)	K, 135 (9.15%)
5th most common			X, 2 (0.14%) *	F, 27 (1.83%)	F, 38 (2.57%)	R, 113 (7.66%)
6th most common			Gap, 2 (0.14%)	M, 10 (0.68%)	G, 27 (1.83%)	N, 96 (6.50%)
7th most common			S, 1 (0.07%)	E, 1 (0.07%)	S, 14 (0.95%)	D, 68 (4.61%)
8th most common				Gap, 1 (0.07%)	V, 12 (0.81%)	S 40 (2.71%)
9th most common					N, 1 (0.07%)	E, 27 (1.83%)
10th most common					Gap, 1 (0.07%)	Q, 23 (1.56%)
11th most common						L, 20 (1.36%)
12th most common						A, 12 (0.81%)
13th most common						H, 9 (0.81%)
14th most common						M, 8 (0.61%)
15th most common						Y, 2 (0.14%)

* "X" meaning an unknown amino acid is present in the sequences *Methylocella palustris (*Q6KCQ3) and *Methylosinus trichosporium* (Q6KCQ2).

Table S4. List of sequences that were used for the visualisation of mitochondrial cleavage sites.

accession	sequence
Q9FNC9	AAKRIGAGKSGGG
Q9FXB9	LILRRTYLTSTGV
Q93WC5	LARRFCATLATAT
Q9M065	RLVRRFASAAADG
Q9LG23	CPIRRFSSAATVV
Q8GYB1	TLLCKSMEPAITA
Q9SKU6	LRRSFLFHSGKTT
Q9ZPF5	PFTTLRSLPTSLV
Q9ZU25	LRPARYASSSAVA
P54967	LQSASCYSSLSAA
Q9SY07	LFSAAAAATVDTA
Q9SMN1	LIRRYFAAEAVAV
Q8LCY2	HRLLNFSSASAIK
Q9LVS3	ISALRFLTTVSAA
O49543	SRCRYLSTAAAAT
Q8H1Y0	SSIRRHVSTDSSP
Q8LBZ7	LIPARWTSTGAEA
O49627	VGILRTYHENVID
Q8LB02	LIPARWTSTGSEA
Q9ZPX5	QLSRFFSAPPSAG
P46643	GGLRSMSSWWKSV
P39697	GSLRYMSTKLYIG
Q9FGK0	SSLRQRAGMGLPV
Q9CA19	GLRVQGIRVGNAE
P32746	GATRNCSSVPGAS
O82663	RFSRFFSTGSTDT
Q9SS48	PSVASSDKGGGPI
Q9SVM8	GSLRLMSTKLFIG
O80543	LASLSFSTEGAYG
Q9SAK4	SQCRQMSMDAQSV
P34893	KSSKLNSGKVIAV
Q9FX83	GLQSRAISYGSNK
Q9C8L4	PTFLRSVMGSSSS
Q42599	RLQSRGISYGSNK
O49354	LHQRLFSTSDTDA
Q9LRN8	FLIRRFSSDTGLM
Q9LY43	ISGRSFSTVNPNP
Q9SGQ6	CSCSRSVTTMIGN
Q9LDV4	ALSRFFSSTSEMS
Q8W485	KYQVRAIQGTSTD
O80983	LPRTRFQSSYVGS
P31166	RLFSSAAASRDSE
Q9LFQ4	GLCSMFSTSIADS
P83291	NNLVYLDQAKEET
Q5XF75	NFIRSFSSEAPPA
Q9FUT3	ARVFFFSTSTSTP
Q94B78	HQQTRSISVDAVK
Q9LRP6	FFGIHKLSSIADA
O80988	QHQTRSISVDALK
Q8LEZ4	TRESRLSESSAAI
Q8L7B5	IIEQSWGAPKVTK
Q1JPN0	AIRRSMATVSQAF
Q9SY69	IPHRTMAFSSAEE
Q8GWR0	VPLYRTFCQATGY
Q9ZUX4	RIVSRRFSSGKVL
P46637	RLNSASFTSVSAS
Q9ZP06	AVIRRSFSSGSVP
Q9M9M6	PSSRRNFSVATTQ
Q9LKA3	GLLRRGFASESVP
Q9FJE6	RNSFRNVSSVIDS
Q9C6I6	SNAPRSISISITS
Q9FJW4	RRVARPFATDAVV
Q9SV46	LRNNKPFCSQSQF
Q8VYR5	LVSRRYFASSPEE
Q9SX99	FPKSRFFSDGESA
Q9SUD8	LLNPRVYSKLVNA
Q38799	LVSARSYAAGAKE
Q3ECB8	FHRLNHFVTTSSS
Q9M7T0	GVSSRGFSKLAEG
Q93ZM7	IISSRNYAAKDIS
P29197	MSWSRNYAAKEIK
Q8S528	NRGAQRYSNLAAA
P53665	LGTIRSFSSHDDH
Q9M5K3	FSLSRGFASSGSD
O80800	SILLRRFSEEVRG
Q9M5K2	FSLTRGFASSGSD
Q8LCU7	TLCIKSFSTIMSP
Q39243	SLASAAFSSSAVM
Q94B59	SRFSKSYNADAAI
Q8LAD2	LVQSRSFGTTPPP
Q9SU63	GGILRRFGTSSAA
Q9FGI6	SKPELQSPESAAV
Q9FGJ4	SIGIFNFTSEAAA
Q9LUC2	SALASLYSTVSGQ
Q8LEE7	LPNVRLLSSDTSS
P68209	LTQSRSFASDPHP
Q9SK66	GTDNCRYSSSLAT
Q0WUC5	LFQSRFFSTPSDL
Q9LDD8	FRGLQKGFCVGIL
Q8GUM2	GYLARPFCSRPVG
F4I7I0	DSSSRHLSSSSSS
Q9LDZ0	RSFSRAFSSKPAG
P83483	LGRVAEYATSSPA

Q9FX93	PTTLRCICSHSSS
Q9LW15	FVIPRRFSSDSVE
P0C2G7	LVIWIRYSSAASS
P92983	SDQARLFSSIPTS
O23240	LQQYKCFGSSAAS
Q8H107	QRWVRPFSAETGD
Q9FLQ4	QRWVRPFSSDSGD
Q0WM29	ALRSSWLSTSPEQ
Q5M729	MQSARGFSSGSDL
Q8RWN9	MRSVRGFSSSSDL
AT1G07830.1	AASARSESTTAAA
AT1G15390.1	KAREVDPGEIGSE
AT1G47420.1	GQVRRFSEDVSHM
AT1G48420.1	LARRSMSATSVPS
AT1G51390.1	PSSLMFSPGKPVM
AT1G59900.1	AAFSRLISTDTTP
AT1G80230.1	SIFSRLIGSAAAD
AT2G17130.1	GDMKSLPEGLLES
AT2G26080.1	QHQTRSISVDALK
AT2G30720.1	RSQKMWNSTVPSD
AT2G31170.1	QLRRCFTTLSSLT
AT2G33040.1	LLGVRSISTQVVR
AT2G35120.1	VAQRGFSSVVLKD
AT2G44350.1	SNSVRWIQMQSST
AT2G44525.1	SLRRAFSLYDQIN
AT3G03600.1	RGNIFFVNTNPLF
AT3G08580.1	KGFTNFALDFLMG
AT3G10920.1	LLRIRGIQTFTLP
AT3G15000.1	CDFEHWLVVVEPP
AT3G22200.1	DMLAPFTAGWQSA
AT3G27380.1	LIPARWTSTGAEA
AT3G52200.1	FGVQNFSSTGPIS
AT3G61530.1	TTVRFMSNVPEDT
AT4G00570.1	GSRRCFSTAIPGP
AT4G22220.1	VGILRTYHENVID
AT4G33010.1	HQQTRSISVDAVK
AT4G37830.1	APKRNFSSSAGHD
AT5G03290.1	STTPITATLFPGD
AT5G10860.1	VFCSRSESTQPAR
AT5G11770.1	TSPTSYTRPGPPS
AT5G13450.1	PALRTYATASAQT
AT5G14590.1	SAVRCFASSGGSD
AT5G15090.1	FSVTTYSSTGVAI
AT5G47030.1	VTTRAFSTELPST
AT5G52520.1	MVIRPYGYAIWEA
AT5G61030.1	IGGMAYSMDEDSL
AT5G63510.1	LIRRCFAAEAALA

Table S5. Sizes for unprocessed and processed NifD proteins and the predicted size of the C-terminal degradation product arising from secondary cleavage by the MPP.

		**size [kDa]** *		
	AA at site	unprocessed	processed	possible degradation product
A. brasilense	RRNYY	60.9	56.3	44.5
A. vinelandii	RRNYY	62.3	57.7	45.8
C. tepidum	RRNQT	67.7	63.1	51.5
D. ferrireducens	RRNKA	67.4	62.8	51.4
D. vulgaris	RRNQT	67.9	63.3	51.2
S. fredii	RRNYY	64.0	59.4	46.4

* the predicted sizes include the HA-tag at the C-terminus.

Supplementary Dataset

Dataset S1. Basic DNA parts and Golden Gate L1 vectors used in this study

Supplementary References

1. Carrie C, Venne AS, Zahedi RP, & Soll J (2015) Identification of cleavage sites and substrate proteins for two mitochondrial intermediate peptidases in Arabidopsis thaliana. *J Exp Bot* 66(9):2691-2708.
2. Huang S, Taylor NL, Whelan J, & Millar AH (2009) Refining the Definition of Plant Mitochondrial Presequences through Analysis of Sorting Signals, N-Terminal Modifications, and Cleavage Motifs. *Plant Physiology* 150(3):1272-1285.
3. Schwarz RF*, et al.* (2016) ALVIS: interactive non-aggregative visualization and explorative analysis of multiple sequence alignments. *Nucleic Acids Research* 44:e77-e77.
4. Felsenstein J (2005) PHYLIP (Phylogeny Inference Package) Department of Genome Sciences. *University of Washington, Seattle, WA [Google Scholar].*
5. Kohl M, Wiese S, & Warscheid B (2011) Cytoscape: software for visualization and analysis of biological networks. *Data mining in proteomics*, (Springer), pp 291-303.
6. Settle B, Otasek D, Morris JH, & Demchak B (2018) aMatReader: Importing adjacency matrices via Cytoscape Automation. *F1000Research* 7.
7. Consortium U (2019) UniProt: a worldwide hub of protein knowledge. *Nucleic acids research* 47(D1):D506-D515.
8. Allen RS*, et al.* (2017) Expression of 16 Nitrogenase Proteins within the Plant Mitochondrial Matrix. *Frontiers in Plant Science* 8(287).
9. Giglione C & Meinnel T (2001) Organellar peptide deformylases: universality of the N-terminal methionine cleavage mechanism. *Trends in plant science* 6(12):566-572.
10. Lister R*, et al.* (2007) Functional definition of outer membrane proteins involved in preprotein import into mitochondria. *Plant Cell* 19(11):3739-3759.
